# Unique Kinase Catalytic Mechanism of AceK with a Single Magnesium Ion

**DOI:** 10.1371/journal.pone.0072048

**Published:** 2013-08-19

**Authors:** Quanjie Li, Jimin Zheng, Hongwei Tan, Xichen Li, Guangju Chen, Zongchao Jia

**Affiliations:** 1 College of Chemistry, Beijing Normal University, Beijing, People’s Republic of China; 2 Department of Biomedical and Molecular Sciences, Queen’s University, Kingston, Ontario, Canada; Instituto de Tecnologica Química e Biológica, UNL, Portugal

## Abstract

Isocitrate dehydrogenase kinase/phosphatase (AceK) is the founding member of the protein phosphorylation system in prokaryotes. Based on the novel and unique structural characteristics of AceK recently uncovered, we sought to understand its kinase reaction mechanism, along with other features involved in the phosphotransfer process. Herein we report density functional theory QM calculations of the mechanism of the phosphotransfer reaction catalysed by AceK. The transition states located by the QM calculations indicate that the phosphorylation reaction, catalysed by AceK, follows a dissociative mechanism with Asp457 serving as the catalytic base to accept the proton delivered by the substrate. Our results also revealed that AceK prefers a single Mg^2+^-containing active site in the phosphotransfer reaction. The catalytic roles of conserved residues in the active site are discussed.

## Introduction

AceK is the first example of reversible protein phosphorylation in prokaryotes [1]. It is a bifunctional enzyme which uniquely presents both kinase and phosphatase activities. AceK represents a more extreme case of kinase diversity compared with the eukaryotic protein kinases (ePKs) superfamily [2]. With the opposing activities at the same highly adaptable active site, AceK regulates the action of isocitrate dehydrogenase (ICDH) and acts as the metabolic switch between the Krebs cycle and the glyoxylate bypass in response to nutrient availability [3].

X-ray crystal structures of AceK and its complex with its substrate ICDH [4] revealed that in the active site there is only one Mg^2+^ ion, which is coordinated by ATP, Asp475 and Asn462. The finding of a single Mg^2+^ ion in the active site represents one of the most striking differences between AceK and ePKs, the latter of which always contain two Mg^2+^ ions intimately involved in the catalysis. One of the Mg^2+^ ions (MgI) is coordinated by the β- and γ-phosphates of ATP, while the second one (MgII) usually binds to α- and β- phosphates of ATP. In ePKs, a histidine residue (or sometimes tyrosine) is responsible for orienting two catalytic aspartate residues to coordinate the ATP molecule and Mg^2+^ ion for catalysis. AceK lacks this histidine residue, yet a flexible glycine residue neighbours the catalytic Asp457, allowing for additional freedom for it to shift in and out of the ATP pocket. The absence of this histidine may also be a reason for the existence of only one Mg^2+^ in AceK, in contrast to two Mg^2+^ ions present in ePKs [2]. The two Mg^2+^ ions in ePKs are thought to play important roles in the phosphotransfer reaction by masking the negative charges of ATP and orienting the γ-phosphate to better interact with substrates. In cyclic-AMP-dependent protein kinase (PKA), one of the best characterized members of ePKs, one Mg^2+^ (MgI) has been generally identified as a catalytic activator, while another (MgII) acts as an inhibitor [5], [6]. The single Mg^2+^ ion in AceK is coordinated by α- and β- phosphates, which has a similar coordination with MgII in PKA. The MgI in ePKs, which is coordinated by the β- and γ- phosphates of ATP was not observed in the active site of AceK since the space for this Mg^2+^ ion is occupied by the ATP [2]. Despite the extensive biochemical and structural investigations [2], it remains elusive whether the kinase catalytic mechanism of AceK with only one Mg^2+^ ion resembles that of ePKs.

Although several classical kinase motifs appear to be absent or modified in AceK, the conserved residues in AceK show many structural similarities to those in ePKs, particularly in the ATP binding region. While the protein kinase superfamily is large and structurally diverse, almost all of the known protein kinases share a highly conserved catalytic core [7], [8]. The same triad is present in AceK, involving residues Asp457, Asn462 and Asp475, in the exact spatial positioning as those in ePKs [2]. In accordance with their critical role in ATP binding and catalysis, mutations in this signature motif completely abolished kinase activity of AceK [4]. In light of the sequence diversity, the close structural similarity of AceK to ePKs was unexpected, but provided a good basis for understanding the kinase function of AceK.

Numerous experimental and theoretical studies have been done to explore the γ-phosphoryl transfer mechanism catalysed by ePKs. According to the most extensively studied PKA which serves as a prototype, there are two most favorable reaction pathways. The first one is that the transfer of the γ-phosphoryl and substrate proton occurs only between the substrate and ATP, which is also known as the associative mechanism [9]–[13]. The second proposed mechanism follows a dissociative path in which the substrate proton is accepted by the catalytic residue Asp166 and the γ-phosphoryl group passes from ATP to the substrate [14]–[20]. Recently work has largely discarded the associative path. In each mechanism, both Mg^2+^ ions in the active site play important roles in the reaction. Given that there is only one Mg^2+^ in the active site of AceK, which pathway is structurally feasible and energetically favorable is an open question and a thorough examination of catalytic mechanism represents a timely study.

In this work, we investigated the phosphotransfer reaction catalyzed by AceK with the hybrid density functional theory (DFT) method B3LYP [20]. The DFT calculations allowed us to explore how the phosphorylation reaction could proceed with the assistance of only one Mg^2+^ ion by analyzing electronic details of the active site in the reaction. Both the associative and dissociative mechanisms were investigated and the results compared. Furthermore, the catalytic roles of individual residues involved in the active site during the phosphorylation reaction were also examined. Our study of the kinase mechanism of AceK offers a new understanding of prokaryotic phosphorylation and the switch between the Krebs cycle and the glyoxylate bypass.

## Models and Methods

### Computational Model

The crystal structure of AceK in complex with substrate ICDH (PDB code: 3LCB) [4] was used for model building. In this structure, the hydroxyl group of Ser113 (residue to be phosphorylated in ICDH) and the γ-phosphate of ATP are not in near-attack position, since Ser113 is buried inside the ICDH structure and is ∼13.2 Å away from ATP. Comparing the AceK-ICDH complex structure with the two PKA-ligand complexed structures(PDB codes: 1L3R and 1ATP) [21], [22], the active-site residues have a similar structure “primed” for the catalyzing process. Therefore, it is rational to reposition the hydroxyl group of Ser113 in the AceK active site based on the relevant ePKs structures. The Ser113 was moved to the position where the hydroxyl group of Ser113 and the γ-phosphate of ATP are close enough to trigger the phosphotransfer reaction. The steric conflict was then released by geometry optimizations.

The enzymatic phosphotransfer process was further examined by considering a cluster model of the AceK active site. The cluster model includes the methyl-triphosphate arm of ATP, Ser113 of ICDH, the single Mg^2+^ ion with its first shell ligands, including three aspartates (Asp457, Asp475, and Asp477), two lysines (Lys336, Lys461) and an asparagine (Asn462). In addition, four important second-shell residues, Gly320, Met321, Val322 and Met323, were also included in the reaction model. Asp475 and Asp477 were protonated to neutralize the condensed negative charges. To complement the octagonal coordination of Mg^2+^ ion, two water molecules were further added. The resulting model possesses a net charge of -1.

### Computational Details

**Figure 1:**
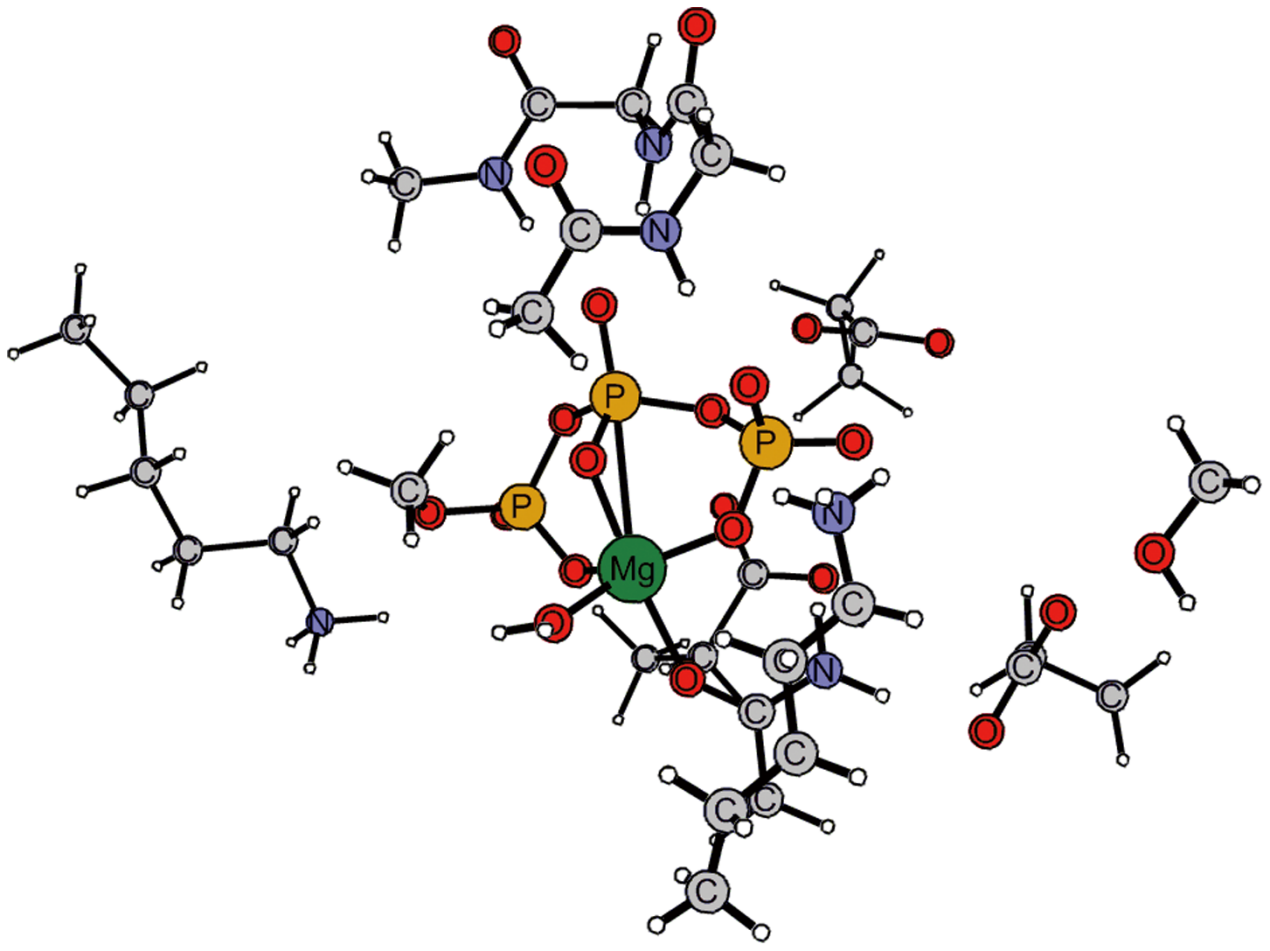
Energy-minimized structure of the active site of AceK in complex with substrate fragment. The cluster model of the active site includes the methyl-triphosphate arm of ATP, the serine113 residue of ICDH, the Mg^2+^ ion and its first shell ligands, including three aspartates (Asp457, Asp475, and Asp477), two lysines (Lys336, Lys461) and an asparagine (Asn462). In addition, four important second-shell residues, Gly320, Met321, Val322 and Met323, are included.

Before extracting the catalytic model, the crystal structure was subjected to energy minimization, in which main chain atoms were excluded and side chains fixed to their experimentally determined position. 5000 steps minimization were carried out by using Amber 9.0 package with Amber99 force field [23]. Since the charge parameters of Mg-ATP complex and AMP are not available in the standard Amber99 force field, they were determined by RESP (Restrained ElectroStatic Potential) fitting from the QM calculations. The system was neutralized by 27 Na^+^ ions. During the optimization, all heavy atoms were constrained. The minimized structure is shown in Figure 1.

Following the initial MM energy minimization, geometry optimizations as well as transition state search were carried out by using the DFT functional B3LYP [20], [24], [25] with 6-31G(d) basis sets. Based on the located stationary point geometries, high accurate energies were further evaluated by the B3LYP* function [26] with larger and nearly saturated basis sets (6-311+G for Mg and cc-pVTZ without f-functions for the rest). The B3LYP* functional is a minor modification of the original B3LYP functional with 15% exact exchange (rather than 20%).

The polarization effects of the protein environment that are not explicitly included in the quantum chemical model were estimated by using the CPCM continuum solvation model. A dielectric constant of 4.0 and a probe radius of 1.4 Å were used. Zero-point energy (ZPE) effects correction was performed at the same level as the geometry optimizations. As will be discussed below, some atoms were fixed during optimization, which gave rise to a few small imaginary frequencies, typically on the order of 5i-37i cm^−1^. These imaginary frequencies attributed to the geometric parameters of the fixed atoms do not significantly contribute to the ZPE and thus can be tolerated. The energies reported herein were corrected for both solvation and zero-point vibrational effects.

All QM calculations were performed using the Gaussian09 program [27]. van del Waals effect was calculated by B3LYP-D using Grimme’s D2 method implemented in XYZ-Viewer.

## Results and Discussion

### Phosphotransfer Step: QM Calculations of the Cluster Model

**Figure 2:**
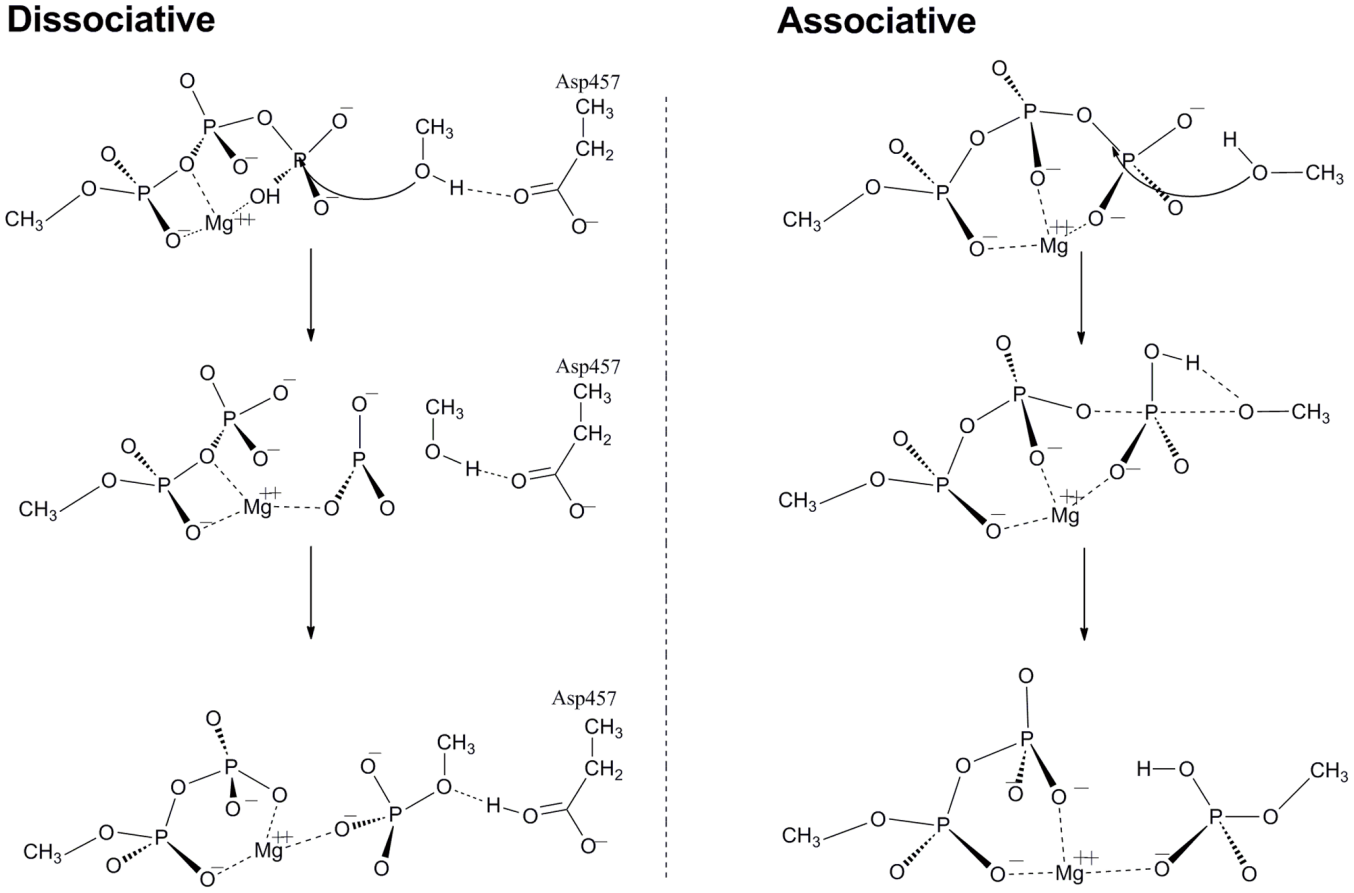
Reaction scheme for the phosphoryl transfer step. The left panel is the dissociative reaction pathway, whereby the proton of Ser113 passes to an oxygen of Asp457 during the nucleophilic displacement. The right panel is the associative mechanism which consists of the phosphoryl- and proton-transfers between the substrate and the ATP.

**Figure 3:**
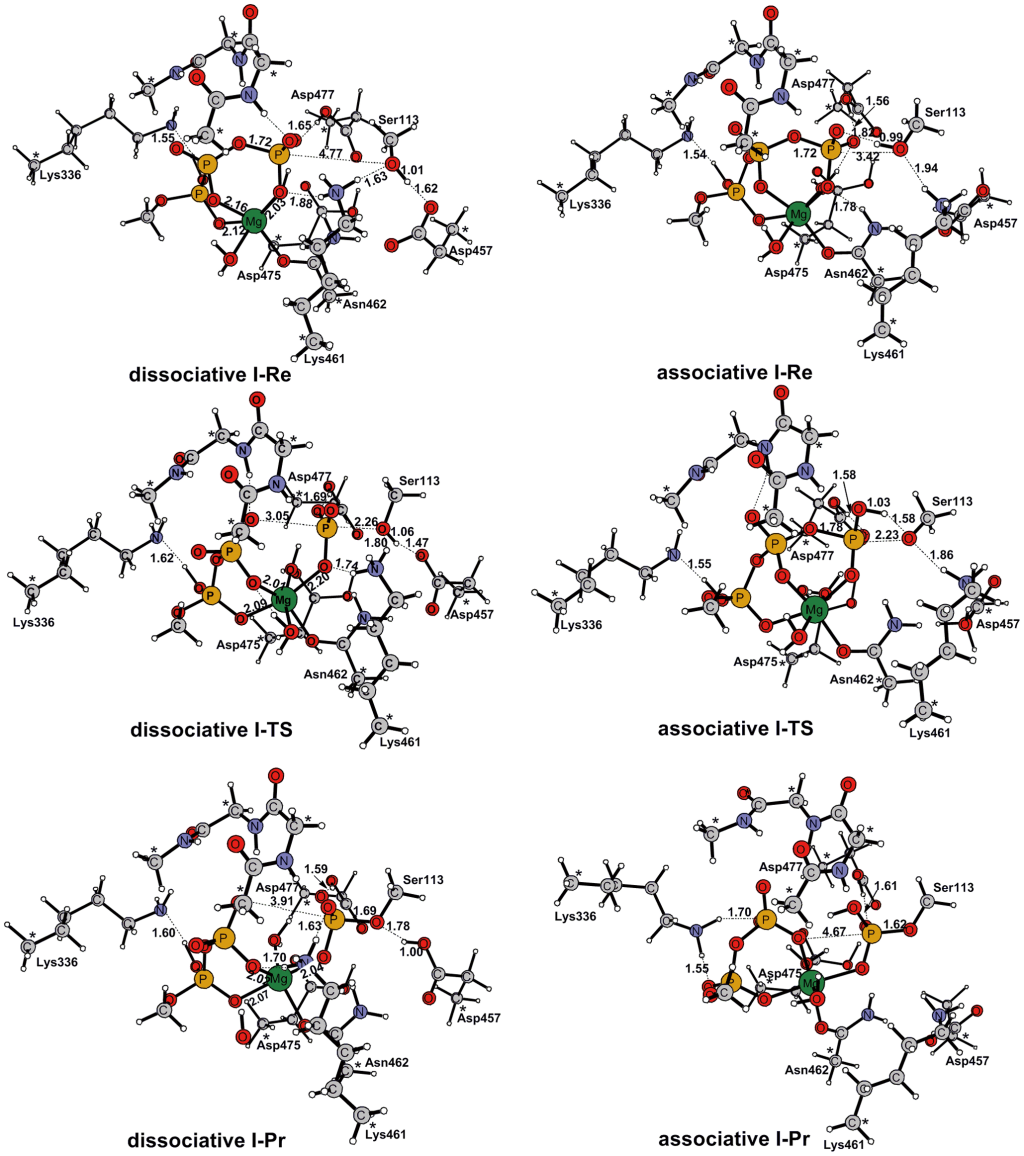
Optimized structures of Re, TS and Pr for the dissociative I and associative I [1 Mg^2+^|2 H_2_O|Asp475-H^+^|Asp477-H^+^] models. Left column: Reactant, Transition state and Product structures located along the Asp457-assisted phosphotransfer reaction (dissociative I). Right column: Reactant, Transition state and Product structures located along the phosphotransfer reaction with direct proton transfer to the γ-phosphoryl group of ATP (associative I).

The Ser113 residue in substrate ICDH is placed in a near-attack position with the γ-phosphate of ATP, the hydroxyl group of Ser113 could form a hydrogen bond with either an oxygen atom of the Asp457 carboxylate side chain or one of the terminal oxygen atoms of the P_γ_ATP. Therefore both configurations have been used as the reactors to explore the potential energy surface (PES) corresponding to the phosphotransfer reaction. As the result, two reaction pathways were identified on the PES. The first one is a dissociative path (Figure 2, **dissociative**), whereas the second one is associative (Figure 2, **associative**). The geometries of the critical structures are shown in Figure 3. The reactant state with the lowest energy is treated as the resting state for the corresponding transition states. The energetics are given in Table 1.

**Table 1:** summary of the calculated energetics for the various models (kcal/mol)^a,b^.

	Dssociative					Associative		
	dissociative I	dissociative II	dissociative III	dissociative IV	dissociative V	associative I c	associative II	associative III
**Re**	0.0	0.0	0.0	0.0	0.0	2.6	0.0	0.0
**TS**	24.7	25.0	30.6	29.0	15.4	32.6	38.7	43.5
**Pr**	−5.4	−6.7	−7.1	8.7	−2.1	−18.2	−18.6	4.0

a Relative energies, with respect to the most stable prereactive complex, of the critical structures for studied models.

b The composition of each model: **dissociative I** [1 Mg^2+^|2 H_2_O|Asp457-H^+^|Asp477-H^+^], **dissociative II** [1 Mg^2+^|1 H_2_O], **dissociative III** [2 Mg^2+^|1 H_2_O| Asp477-H^+^], **dissociative IV**[1 Mg^2+^|1 H_2_O|Asp457-H^+^|Asp477-H^+^], **dissociative V** [1 Mg^2+^|2 H_2_O|Asp457-H^+^|Asp477-removed], **associative I** [1 Mg^2+^|2 H_2_O|Asp457-H^+^|Asp477-H^+^], **associative II** [2 Mg^2+^|1 H_2_O], **associative III** [2 Mg^2+^|4 H_2_O].

c The energies of associative I are respect to dissociative I. The composition of associative I and dissociative I are the same.

### Dissociative Mechanism

Dissociative mechanism is identified as the most energetically favorable path for the phosphotransfer reaction. In the optimized structure of the reactant complex (**dissociative** **I-Re** in Figure 3), the O atom of the nucleophilic hydroxyl group of the Ser113 side chain is 4.77 Å away from the P atom of γ-phosphate and the substrate hydroxyl group interacts with the carboxylate group of the essential Asp457 through a short hydrogen bond (H_γ_Ser_113_-O_δ_1Asp457 = 1.62Å). In addition, the amino group of the Lys461 side chain interacts directly with two key moieties in the active site: the γ-phosphoryl group (1.88 Å) and the substrate hydroxyl group (1.63 Å). Further, the side chain of the protonated Asp477 also forms a hydrogen bond with the γ-phosphate oxygen atom and the distance is 1.65 Å. The tight network of interactions in the active site ensures near-attack conformations of the residues which are important in the reaction process. In **dissociative** **I-Re**, the Mg^2+^ ion is located at the center of the reaction system and is coordinated by Asn462, α-, β- and γ- phosphoryl groups of ATP, and two water molecules. The relative orientation of the nucleophilic hydroxyl group and the terminal phosphoryl group as well as the hydrogen bond contact with the carboxylate group observed in **dissociative** **I-Re** are favorable for a nucleophilic displacement with proton transfer from the hydroxyl to the carboxylate group.

In the transition state (**dissociative** **I-TS**), the distance of O_γ_Ser113-P_γ_ATP is dramatically decreased from 4.77 Å in **dissociative** **I-Re** to 2.26 Å, while the O_3β_ATP-P_γ_ATP distance is elongated from 1.72 Å to 3.05 Å. The P_γ_O_3_ group occupies a slightly asymmetrical position and is pushed closer to the entering substrate hydroxyl group than the leaving ADP group. Remarkably, at the transition state, proton transfer is at its initial stage, which is still attached to the bridging oxygen with a distance of 1.06 Å (1.01 Å in **dissociative** **I-Re**). However, the hydrogen-bond distance between Asp457 and Ser113 decreased from 1.62 Å in **dissociative** **I-Re** to 1.47 Å in **dissociative I-TS,** which is important for the proton transfer after the transition state. Hence, we can consider that **dissociative** **I-TS** has a high degree of dissociative characteristics, exemplified by the fact that the proton can be transferred to the Asp457 side chain in a concerted fashion with the nucleophilic substitution. The distance of Lys461 to the γ-phosphoryl group of the ATP and the substrate hydroxyl group are 1.74 Å and 1.80 Å, respectively. The hydrogen-bond between the protonated Asp477 and the γ-phosphoryl group remains largely unchanged in moving to the reactant state (Figure 3).

The movement of the Mg^2+^ ion is an important structural feature that accompanies the reaction process of dissociative mechanism. In the located transition state, the Mg^2+^ moves toward the β-phosphoryl group of the ATP, since a distance reduction between the Mg^2+^ and O_1β_ (from 2.16 Å to 2.01 Å) were observed. The γ-phosphoryl group continued to interact with Mg^2+^ and the distance between Mg^2+^ and O_1γ_ increased to 2.20 Å (compared to 2.03 Å in the reactant state). This movement activates the cleavage of the O_β_-P_γ_ bond by stabilizing the leaving ADP group. It also triggers the formation of a highly electrophilic metaphosphate PO_3_ group in the transition state by masking the negative charge accumulated in β-phosphate.

Frequency calculations confirmed that the transition vector at **dissociative** **I-TS** (ν = 109.74 icm^−1^) has a contribution from movement of Ser113 hydroxyl hydrogen, which is dominated by a torsional movement of the carboxylate side chain of Asp457. Energetically, **dissociative** **I-TS** has a barrier of 24.7 kcal mol^−1^ with respect to **dissociative** **I-Re**.

In the product complexes obtained for the phosphotransfer reaction (**dissociative** **I-Pr**), the γ-phosphate completely transferred to the Ser113 side chain (O_γ_Ser113-P_γ_ATP = 1.69 Å). The optimized O_3β_ATP^…^P_γ_ATP distance of 3.91 Å is longer than the corresponding van der Waals distance (3.3 Å), which suggests a complete O-P bond breakage. In addition, after passing TS, the proton transfer occurs as the hydroxyl group of Ser113 donates its proton to the carboxylate group of the essential Asp457; the protonated Asp457 side chain interacts closely with the O_γ_Ser113 atom through a short hydrogen-bond with a distance of 1.78 Å. The transferred γ-phosphoryl group continues to interact with Lys461 in the product state. The distance to the oxygen of the γ-phosphoryl group is 1.63 Å, which is shorter than the values observed in the reactant and transition states. This describes an increased strength of the interaction between Lys461 and the γ-phosphoryl group. Notably, the amino group of the Lys461 side chain no longer interacts with the hydroxyl group of Ser113 anymore. Instead, it forms a short hydrogen bond with the β-phosphoryl group (HζLys461-O_1γ_ATP = 1.70 Å). In **dissociative** **I-Pr**, the protonated Asp477 also moves closer to ATP and hydrogen bonds to the γ-phosphate with a distance of 1.59 Å. The coordination of the Mg^2+^ ion is slightly distorted and becomes 5-fold coordinated due to its loss of interaction with one of the water molecules. Nevertheless, the Mg^2+^ still maintains its coordination to the O_α1_, O_β1_ and O_γ1_ of ATP with the final distances of 2.07, 2.02 and 2.04 Å, respectively. Energetically, **dissociative** **I-Pr** is 5.4 kcal mol^−1^ more stable than **dissociative** **I-Re**.

### Associative Mechanism

The second mechanism investigated in this study starts at the pre-reactive complex **associative** **I-Re** shown in Figure 3. In the reactant, the nucleophilic serine side chain interacts with one of the oxygen atoms of the γ-phosphoryl group of ATP through a hydrogen bond (H_γ_Ser113-O_1γ_ATP = 1.82 Å). Other interactions in **associative** **I-Re** and **dissociative** **I-Re** are similar although the reactive P_γ_-O_γ_Ser113 distance for nucleophilic displacement in **associative** **I-Re** is quite short (3.42 Å). Energetically, **associative** **I-Re** is slightly less favored than **dissociative** **I-Re** by 2.6 kcal mol^−1^ (Table 1).

This pre-reactive complex can evolve through a transition state for phosphotransfer with simultaneous protonation of the forming phosphorylated group. Geometrically, the O_γ_Ser113-P_γ_ATP distance at **associative** **I-TS** (2.23 Å) is similar to that at **dissociative** **I-TS** (2.26 Å), whereas the breaking O_3β_ATP-P_γ_ATP distance at **associative** **I-TS** (1.78 Å) is much shorter than that at **dissociative** **I-TS** (3.05 Å). Hence, the **associative I-TS** has a lower dissociative character compared with **dissociative I-TS**. In addition, the hydrogen atom of the nucleophilic hydroxyl group is virtually transferred to the O_1γ_ oxygen atom of the γ-phosphoryl group (H_γ_Ser113-O_1γ_ATP = 1.03 Å). The transition vector at **associative** **I-TS** (ν = 275.83 icm^−1^) has a large contribution from the movement of the transferred hydrogen atom that accompanies the inversion of configuration at Pγ during the nucleophilic substitution. Energetically, **associative** **I-TS** is 32.6 kcal mol^−1^ higher than the pre-reactive complex **dissociative** **I-Re**. Such a high energy barrier associated with **associative** **I-TS** may be related to its higher associative character which, in turn, determines a strained conformation of the reacting atoms. This strained conformation in **associative** **I-TS** is well illustrated by several critical bond angles (H_γ_Ser113-O_1γ_ATP-P_γ_ATP = 95°, O_1γ_ATP-P_γ_ATP-O_γ_Ser113 = 74°, P_γ_ATP-O_γ_Ser113-H_γ_Ser113 = 60°). In contrast, the more dissociative character of **dissociative** **I-TS** and the absence of the four-membered ring for the hydrogen-shift process can explain the large energy difference between the critical **dissociative** **I-TS** and **associative** **I-TS** structures for the phosphotransfer event. **Associative** **I-TS** is connected to the **associative** **I-Pr** in which the γ-phosphoryl group is completely transferred to the Ser113 side chain (P_γ_ATP-O_γ_Ser113 = 1.62Å and P_γ_ATPO_3β_-ATP = 4.67Å) without dramatically distorting other interactions present in the previous **associative** **I-Re** and **associative** **I-TS** structures. Energetically, **associative** **I-Pr** is 15.6 kcal mol^−1^ more stable than **associative** **I-Re**.

A Fukui function [28], [29] for Pγ in dissociative and associative reaction was calculated (Text S1 and Table S1), and the results indicate that the P_γ_ site is more susceptible in the dissociative I model for nucleophilic attack, which also suggests that the phosphotransfer reaction catalysed by AceK follows a dissociative mechanism.

### Single Magnesium Ion Represents the only Viable Pathway in AceK

It is noted that all crystal structures of PKA ternary complexes contain two Mg^2+^ ions. PKA is usually considered as a prototype of the catalytic core for the entire protein kinase family. As a result, the requirement of two Mg^2+^ ions for catalytic activity is well established [21], [22]. However, AceK structure revealed that there is only one Mg^2+^ ion that acts as the structural anchor to facilitate the proper orientation of the ATP with respect to the substrate hydroxyl group. To investigate how the AceK could accomplish the catalysis with the assistant of only one Mg^2+^ ion, we constructed four other models which differ in the number of Mg^2+^ ions and the protonation state of the aspartates based on the previous reaction model.

**Figure 4:**
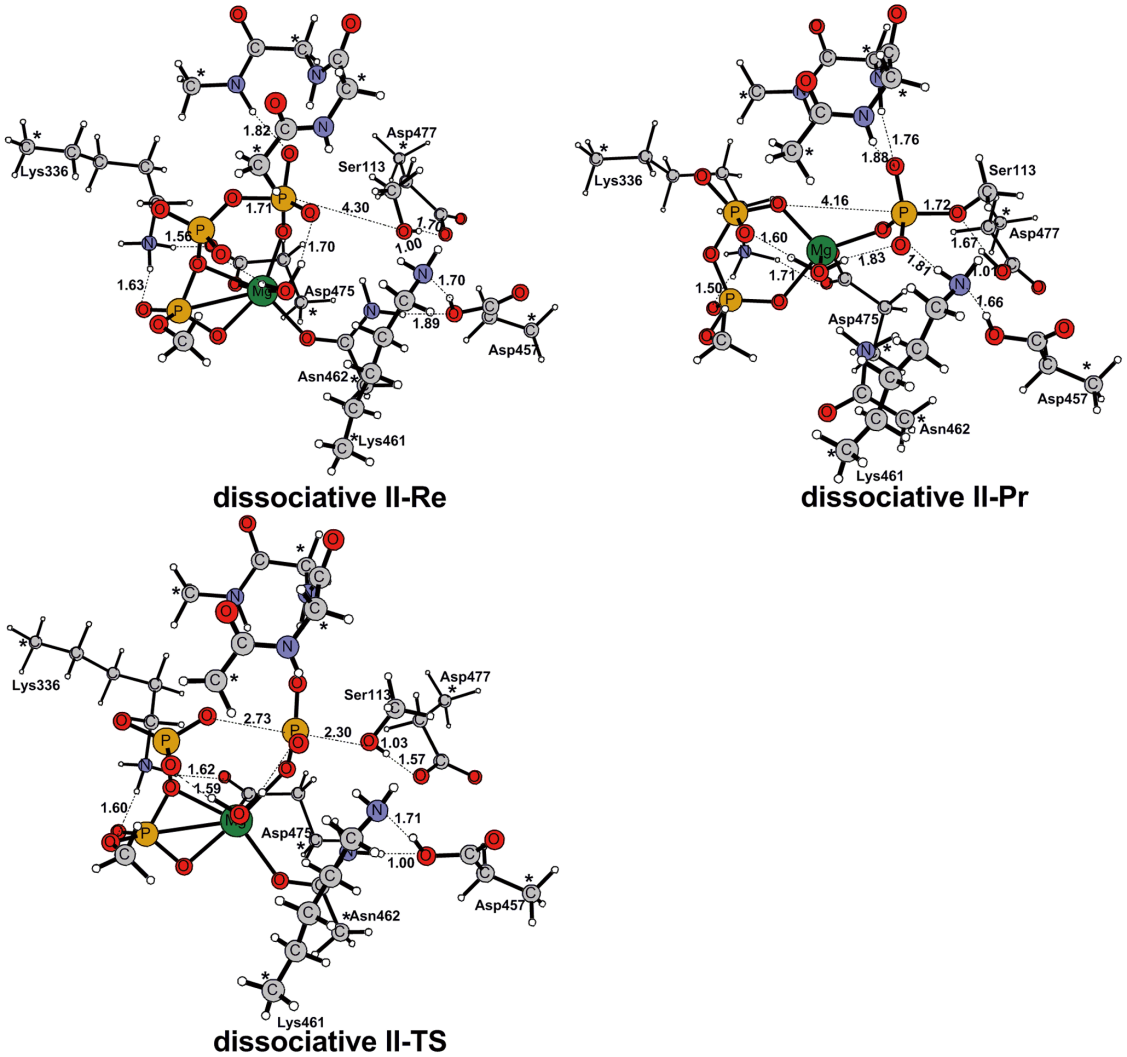
Optimized structures of Re, TS and Pr for the dissociative II [1 Mg^2+^|1 H_2_O] model. There is only one Mg^2+^ion in this model and Asp475 and Asp477 are not protonated. Dissociative II model has a total charge of -3. Our calculation indicated that the Asp477 rather than Asp457 would serve as the catalytic base to accept the proton delivered by the Ser113 in this model. This process is similar to that found in dissociative I model.

Two negatively charged residues Asp475, Asp477 were protonated in **dissociative** **I** model to neutralize the active site. We tested the influence of these two protons on the reaction with **dissociative** **II** model, in which there is only one Mg^2+^ion and the proton on Asp475 and Asp477 was removed. Our calculation indicated that the Asp477 rather than Asp457 would serve as the catalytic base to accept the proton delivered by the Ser113 in this model. This process is similar to that found in **dissociative** **I** model. The optimized geometries of critical structures studied in **dissociative** **II** model are shown in Figure 4.

**Figure 5:**
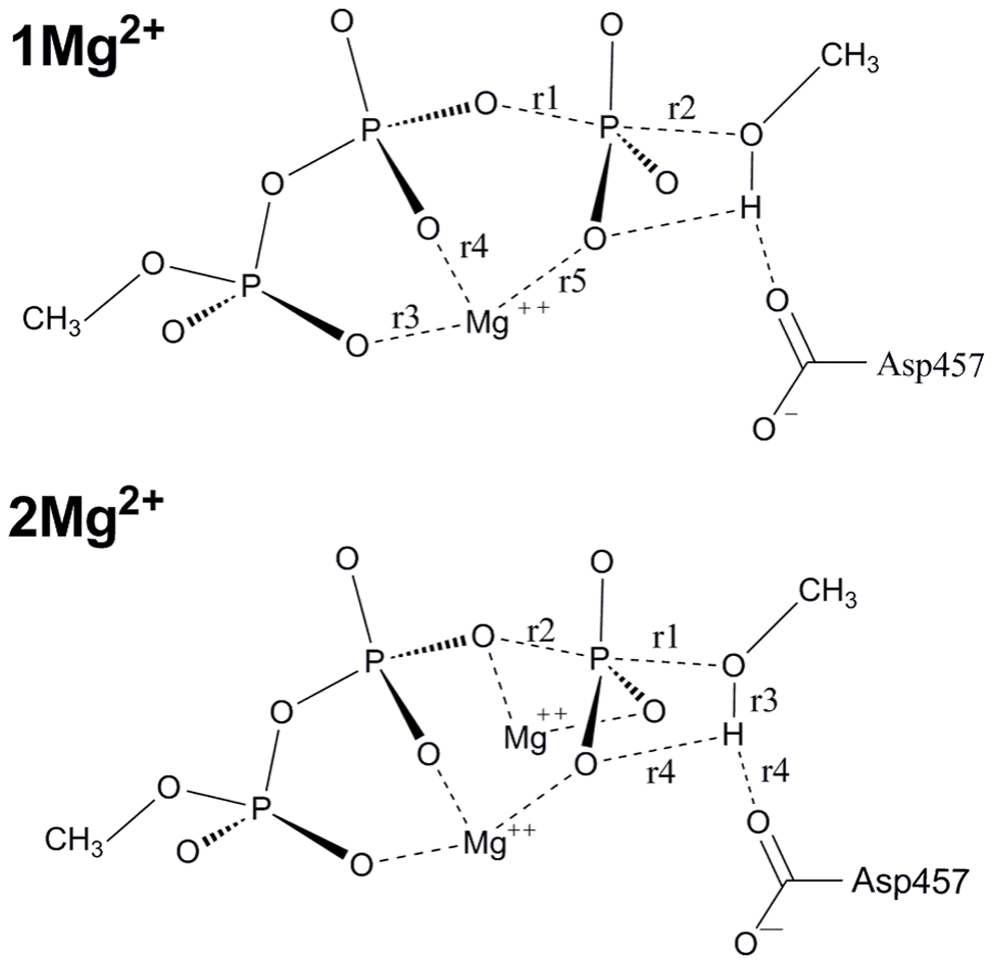
Schematic diagram of 1 Mg^2+^ and 2 Mg^2+^ models. 1****Mg^2+^ model contains only one Mg^2+^ ion, here it specially refers to dissociative I and associative I models. The 2****Mg^2+^ models contain two Mg^2+^ ions, which include the associative II, associative III and dissociative III models.

Since ePKs prefer two Mg^2+^ ion-containing active site, we also investigated the reaction with two Mg^2+^ in AceK. The second Mg^2+^ was manually added in the active site in a similar orientation to that observed in ePKs. In this case, the reaction could also follow both the associative and dissociative mechanisms, which resulted in the **associative** **II**, **associative** **III** and **dissociative** **III** models. In **associative** **II** model, the added Mg^2+^ ion was coordinated by the β- and γ-phosphates of ATP. Based on **associative** **II** model, three more water molecules were added to fulfill the octahedral coordination for second Mg^2+^ to produce **associative** **III** model. In **dissociative** **III** model, effect of charge is examined by protonating Asp477. The detailed structure information of the three models are provided in Figure S1, S2, and S3. In Table 2 and Figure 5, the relevant bond length and associated structure parameters for the various stationary points along the reaction pathways are given to compare the resultant reaction mechanisms.

**Table 2:** Important distance (Å) for the various stationary points along the reaction pathways^a^.

	associative II			associative III			dissociative III		
	Re	TS	P r	Re	TS	P r	Re	TS	P r
**r1**	3.66	2.22	1.64	3.79	2.16	1.65	3.80	2.31	1.68
**r2**	1.67	1.76	4.15	1.64	1.85	3.54	1.68	2.07	4.18
**r3**	0.99	1.53	3.03	1.00	1.63	3.12	0.99	1.02	1.75
**r4** a	1.81	1.04	0.97	1.64	1.00	0.99	1.81	1.57	1.00

a see Figure 5. 2****Mg^2+^.

The potential energy profiles are also summarized in Table 1. Despite different models and different partition schemes, the calculations provide a consistent picture. Models in which there are two Mg^2+^ ions in the active sites need to cross higher energy barriers than those containing only one, which means that one Mg^2+^ ion in AceK is preferred and implies a novel single Mg^2+^-dependent kinase mechanism.

Since there is only one Mg^2+^ ion and one more aspartate (Asp477) in AceK, the concentration of the negative charge in the active site is a unique character of AceK when compared with ePKs. However, our calculations show that the participation of this single Mg^2+^ ion efficiently stabilize the transition state. As is seen in Figure 3, in both the reactant and the transition states, the Mg^2+^ ion is located at the center of the reaction system, 6-fold coordinated by Asn462, α-, β- and γ-phosphoryl groups of ATP, and two water molecules. In the final product, Mg^2+^ becomes 5-fold coordinated due to a loss of one water molecule. The same phenomenon could also be found in **dissociative** **II** model (Figure 4). These results strongly suggest that the water molecules coordinating Mg^2+^ play essential roles in releasing the phosphorylated Ser113. Removing one of the water molecules, which does not coordinate Mg^2+^ in the product, would result in a 4.3 kcal mol^−1^ higher TS (**dissociative** **IV** model, Figure S4), suggesting that the 6-fold coordination of Mg^2+^ is essential for the phosphorylation reaction. Moreover, water molecules are expected to play an important role in stabilization of the transition state and timing of the phosphotransfer reaction. In agreement with this observation, Hirano *et al.* studied the catalytic mechanism of PKA with assistance of single Mg^2+^ ion and found that only when Mg^2+^ is 6-fold coordinated, could the release of the phosphorylated substrate occur [11]. In the models in which the second Mg^2+^ ion is added, the protein is unable to maintain the full octahedral coordination for the two Mg^2+^ ions throughout the reaction. Furthermore, examination of the reaction pathway geometries reveals that there is no sufficient space to contain the phosphorylated serine residue.

### Catalytic Roles of the Critical Active-site Residues

#### Asp457

The carboxylate group of Asp457 is a candidate to accept the hydroxyl hydrogen atom from the nucleophilic serine side chain. After residue Ser113 was repositioned near the γ-phosphoryl group of ATP, there may be a hydrogen bond between the P-site serine side chains and the invariant aspartate Asp457. Substitution of the Asp457 by alanine completely abolished kinase activity, which indicates that Asp457 is an invariant residue involved in ATP binding [4]. Our DFT study of the reaction mechanisms using the cluster models of the AceK active site also reveals that the Asp457 side chain serves as a catalytic base to accept substrate proton later during the phosphorylation process. In **dissociative** **I** model (Figure 3), Asp457 forms a hydrogen bond to the substrate hydroxyl group with a distance of 1.62 Å in the reactant. The strength of this interaction increases in the transition state (H_γ_Ser113-O_δ_1Asp457 = 1.52Å). This change contributes to the stabilization of transition state and initiates the abstraction of the substrate proton. Most interestingly, the serine hydroxyl hydrogen atom has not been transferred to the Asp457 side chain at **dissociative** **I-TS**. This suggests that Asp457 does not abstract the substrate proton in the early stage of reaction process but may play a role in maintaining a favorable orientation of the substrate with respect to the ATP. After passing through **dissociative** **I-TS**, along with the bond formation between the γ-phosphoryl group and the Ser113 side chain, the acidity of the Ser113 hydroxyl group is increasing, and finally the proton will be delivered from the hydroxyl to the carboxylate group of Asp457. As shown in Table 3 and Figure 5, the Wiberg bond index matrix represents strength of bonding interaction between atom pairs. As shown, the P_γ_-OATP bond (r1) is completely broken at the TS for the dissociative mechanism (Asp457 acts as the base), and partially broken for the associative mechanism (ATP acts as the base). The reason is probably that Asp457 serves as a better base. For both mechanisms, the P_γ_-O_Ser_ bond is only partially formed at the TS.

**Table 3:** Wiberg bond index matrix^a^.

structure	state	r1	r2	r3	r4	r5
**Asp45 (Dissociative I)**	Re	0.5080	0.0001	0.1557	0.1404	0.1760
	TS	0.0348	0.1589	0.1607	0.1779	0.1263
	Pr	0.0028	0.5604	0.1334	0.1396	0.1398
**ATP (Associative I)**	Re	0.4985	0.0069	0.1615	0.1362	0.1789
	TS	0.4501	0.2375	0.1551	0.1505	0.1730
	Pr	0.0003	0.6761	0.1706	0.1673	0.1579

a see Figure 5. 1****Mg^2+^.

These observations are consistent with a concerted catalytic base assignment for Asp457. A similar role has been suggested for Asp166 in PKA [14]–[16] and Asp1131 in insulin receptor tyrosine kinase (IRK) [19].

#### Lys461

Lys461 is another important residue in the AceK active site, which is located in close proximity to the hydroxyl group of Ser113. Lys461 interacts directly with one of the γ-phosphate oxygen atom before and after the phosphotransfer reaction. As seen in Figure 3, in reactant, Lys461 interacts with ATP and Ser113 through two hydrogen bonds. The hydrogen bond formed between Lys461 and γ-phosphate is sufficiently stable to be identified in the reactant, transition state and final product. The second hydrogen bond between Lys461 and Ser113, though finally broken in the product, lasts through reactant and transition state. This observation suggests that Lys461 plays a significant role in binding of the reactants and keeping them in close contact conformation. Another interesting finding is that Lys461 moves closer to the ATP at the transition state and away from Ser113 along the reaction path. As seen in Figure 3, the hydrogen bond distance between Lys461 and the γ-phosphate decrease from 1.88 Å in **dissociative** **I-Re** to 1.74 Å in **dissociative** **I-TS** and further to 1.63 Å in **dissociative** **I-Pr**_._ The positively charged Lys461 appears to provide electrostatic stabilization to this emerging negative charge of the phosphate group during the P_γ_ATP-O_3β_ATP cleavage by being part of a hydrogen bonding network involving the γ-phosphate of ATP and the hydroxyl group of Ser113. Although Lys461 does not directly participate in the phosphorylation reaction, the presence of hydrogen bond interactions between its side chain with both the γ-phosphate and the hydroxyl group of Ser113 implies its involvement in the catalytic process. The catalytic role is similar to Lys168 in PKA [15], [17], [30]-[32] and Arg1136 in the insulin receptor tyrosine kinase [19].

#### Lys336

Proton transfer from Lys336 to α-phosphoric acids is another interesting event taking place in the reaction. As seen in Figure 3, the protonated Lys336 is found to be unstable such that its proton transfers to the α-phosphoric acid and leads to subsequent protonation of the phosphate oxygen. By comparing the high level single point energies of two states (proton in Lys336 or in α-phosphoric acids), we confirm that protonation of the phosphate oxygen is favorable. The same phenomenon in ePKs has also been reported by Hirano et al [11]. Although the proton is transferred to the α-phosphate, it still interacts with the amino group of Lys336 through a short hydrogen bond. Shown in Figure 3, the distance between Lys336 and α-phosphate is measured at 1.55 Å, 1.62 Å and 1.60 Å in **dissociative** **I-Re**, **dissociative** **I-TS** and **dissociative** **I-Pr**, respectively, which is slightly changed during the phosphotransfer reaction. Since Lys336 does not participate in the phosphorylation reaction directly, we propose that the main role of Lys336 is holding ATP in proper conformation and stabilizing the transition state through electrostatic interactions. This finding is consistent with mutation studies showing that the replacement of Lys336 with Ala leads to decrease of the AceK kinase activity [4].

#### Asp477

Asp477 is another important residue identified, which is located at ATP binding site. Mutagenesis studies indicated that its mutations to Ala and Asn show decreased phosphatase activity but retained or increased kinase activity [4]. To study the influence of Asp477 on the kinase activity, we calculated the energy barrier by removing it from the **dissociative** **I** model. The resulting energy barrier is 9.3 kcal/mol lower than that of **dissociative** **I** model, which suggests that the invariant residue Asp477 acts as an inhibitor during the phosphorylation reaction (**dissociative** **V**, Figure S5). Notably, in **dissociative** **I** model, Asp477 is protonated. We failed to locate the TS after removing the proton from the model. This means that protonation of Asp477 is indispensable during the phosphorylation reaction, which is equivalent to Asn since both these residues form hydrogen bonds with ATP. As seen in Figure 3, the hydrogen bond between the protonated Asp477 and γ-phosphate oxygen atom is measured 1.65Å, 1.69 Å and 1.59 Å in the reactant, transition state and product of **dissociative** **I** model, respectively. The strong hydrogen bond interaction between protonated Asp477 and the γ-phosphate provides additional structural stabilization, which is favorable for the reaction.

The loop encompassing residues 320–323 constitutes a highly conserved motif within a kinase catalytic core. Owing to its extended “U” shape spatially aligned with ATP triphosphate tail, the loop is capable of tightly enfolding the nucleotide by means of both hydrogen bonding and hydrophobic interactions. As shown in Figure 3, it forms two hydrogen bonds through its amide hydrogen with the β- and γ-phosphoryl group of ATP throughout the phosphotransfer reaction. These structures provide direct evidence that the loop is required not only for binding of MgATP but also for correctly orienting the γ-phosphate of ATP for nucleophilic attack and stabilizing the transition state.

### Conclusions

In summary, through systematically investigating various models of the AceK system, its catalytic reaction of phosphotransfer process is discovered to be a dissociative mechanism. Our calculation shows that the phosphotransfer reaction catalyzed by AceK prefers only one Mg^2+^ ion as the second Mg^2+^ ion will unfavorably increase the reaction barrier.

Analysis of the structural change along with our calculated reaction pathway suggests that the carboxylate group of the invariant Asp457 participates in substrate binding, orients the hydroxyl group of the nucleophilic Ser113, and accepts its proton later during the phosphotransfer process through a dissociative mechanism. This theoretical picture of the reaction mechanism, in which Asp457 behaves as an acid/base catalyst, is in agreement with our own experimental results and those from literature concerning the phosphotransfer reaction of protein kinases. Furthermore, the reaction pathway suggested by our computation study is similar to that found in the cyclic AMP-dependent serine/threonine and even tyrosine kinases, indicating that the eukaryotic kinase and AceK might share a similar mechanism despite the key difference in the number of Mg^2+^ ions in the active site.

The catalytic roles of other active-site residues have also been identified. Lys461 interacts with the γ-phosphate of ATP throughout the reaction, helps maintain substrate and ATP in the near-attack reactive conformation and provides electrostatic stabilization during phosphate transfer by taking part in a hydrogen bonding network.

## Supporting Information

Figure S1 **Optimized structures of Re, TS and Pr for the associative II [2 Mg^2+^|1 H_2_O] model.** In associative II model, the second Mg^2+^ was manually added in the active site akin to ePKs. Associative II model has a total charge of -1 and share the same reaction pathway with associative I model. The mechanism start at a configuration in which the serine side chain forms a hydrogen bond with the oxygen atom of the γ-phosphoryl group of ATP(H_γ_Ser113-O_1γ_ATP = 1.81 Å). In the transition state structures, the proton has been already transferred from Ser113 to O_γ_ATP (H_γ_Ser113-O_1γ_ATP = 1.04 Å), while the γ-phosphoryl group is still bonded to the ATP molecule (O_3β_ATP-P_γ_ATP = 1.76 Å).
(TIF)

Figure S2 **Optimized structures of Re, TS and Pr for the associative III [2 Mg^2+^|4 H_2_O] model.** Based on associative II model, three more water molecules were added to fulfill the octahedral coordination for second Mg^2+^ to produce associative III model. This model also has a total charge of -1 and share the same reaction pathway with associative I model. The mechanism start at a configuration in which the serine side chain forms a hydrogen bond with the oxygen atom of the γ-phosphoryl group of ATP(H_γ_Ser113-O_1γ_ATP = 1.64 Å). In the transition state structures, the proton has been already transferred from Ser113 to O_γ_ATP (H_γ_Ser113-O_1γ_ATP = 1.00 Å), while the γ-phosphoryl group is still bonded to the ATP molecule (O_3β_ATP-P_γ_ATP = 1.85 Å).
(TIF)

Figure S3 **Optimized structures of Re, TS and Pr for the dissociative III [2 Mg^2+^|1 H_2_O| Asp477-H^+^] model.** In dissociative III, the negative charged residue Asp477 was protonated in comparison with associative II model. Dissociative III is the only neutral model and processes a dissociative path, just like the dissociative I model. The proton is still attached to the bridging oxygen O_γ_Ser113 (H_γ_Ser113-O_γ_Ser113 = 1.02 Å) and the distance of O_3β_ATP-P_γ_ATP is elongated from 1.68 to 2.07 Å in the transition state. It is a concerted mechanism involving a late proton transfer to Asp457.
(TIF)

Figure S4 **Optimized structures of Re, TS and Pr for the dissociative IV [1 Mg^2+^|1 H_2_O|Asp457-H^+^|Asp477-H^+^] model.** Based on dissociative I model, one of the water molecules which does not coordinate Mg^2+^ in the product was removed from the active site. The resulting model, dissociative IV, share the same reaction pathway with dissociative I model. However, dissociative IV model results in a 4.3 kcal mol^−1^ higher TS than dissociative I model. This suggests that the 6-fold coordination of Mg^2+^ is essential for the phosphorylation reaction. Moreover, water molecules are expected to play an important role in stabilization of the transition state and timing of the phosphotransfer reaction.
(TIFF)

Figure S5 **Optimized structures of Re, TS and Pr for the dissociative V [1 Mg2+|2 H2O|Asp457-H+|Asp477-removed] model.** To study the influence of Asp477 on the kinase activity, we calculated the energy barrier by removing it out of the dissociative I model. The resulting model dissociative V share the same reaction pathway with dissociative I model. However, the energy barrier is 9.3 kcal/mol lower than that of dissociative I model. This suggests that the invariant residue Asp477 acts as an inhibitor during the phosphorylation reaction.
(TIF)

Table S1 **The conceptual DFT indices of P_γ_.**
(TIF)

Text S1 **Fukui function of Pγ in dissociative and associative reaction.**
(PDF)

Text S2 **Cartesian coordinates for all structures.**
(PDF)
