# Supplementary figures and images for: Unique Kinase Catalytic Mechanism of AceK with a Single Magnesium Ion
Source: PLoS One. 2013 Aug 19;8(8):e72048. doi: 10.1371/journal.pone.0072048 (PMC3747045; doi:10.1371/journal.pone.0072048)

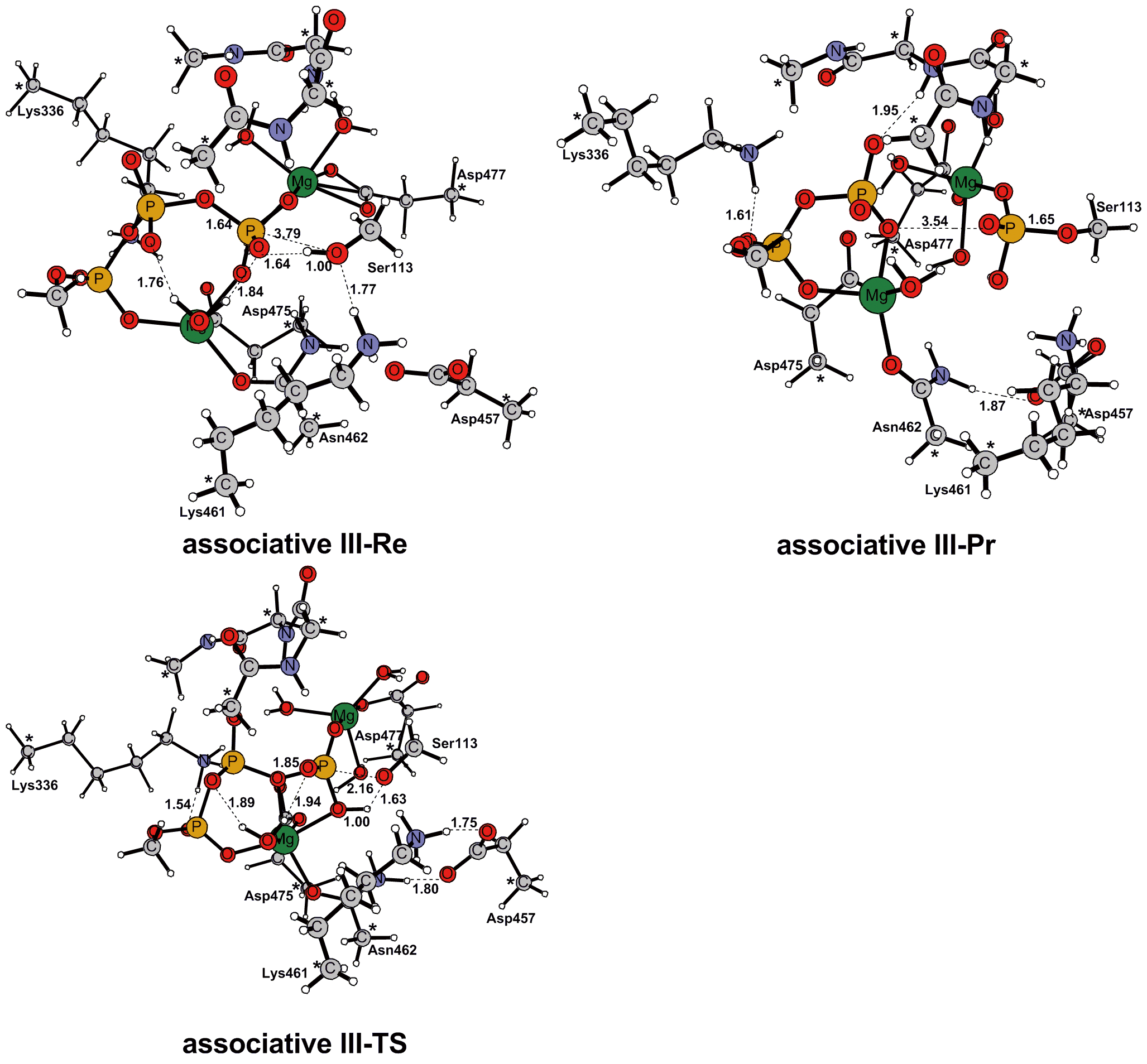

Supplement: Figure S2 — Optimized structures of Re, TS and Pr for the associative III [2 Mg2+|4 H2O] model. Based on associative II model, three more water molecules were added to fulfill the octahedral coordination for second Mg2+ to produce associative III model. This model also has a total charge of -1 and share the same reaction pathway with associative I model. The mechanism start at a configuration in which the serine side chain forms a hydrogen bond with the oxygen atom of the γ-phosphoryl group of ATP(HγSer113-O1γATP = 1.64 Å). In the transition state structures, the proton has been already transferred from Ser113 to OγATP (HγSer113-O1γATP = 1.00 Å), while the γ-phosphoryl group is still bonded to the ATP molecule (O3βATP-PγATP = 1.85 Å). (TIF) [file pone.0072048.s002.tif]

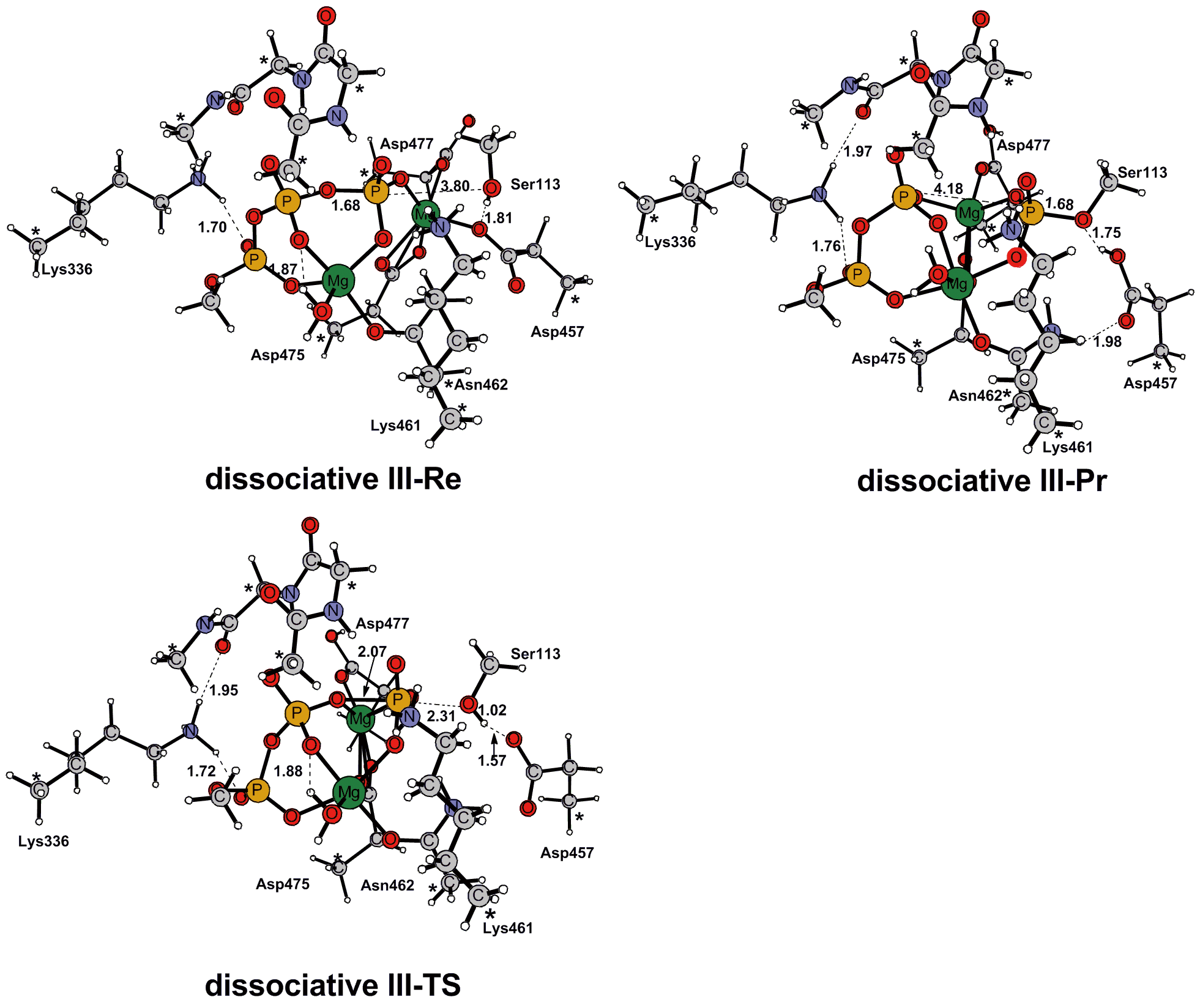

Supplement: Figure S3 — Optimized structures of Re, TS and Pr for the dissociative III [2 Mg2+|1 H2O| Asp477-H+] model. In dissociative III, the negative charged residue Asp477 was protonated in comparison with associative II model. Dissociative III is the only neutral model and processes a dissociative path, just like the dissociative I model. The proton is still attached to the bridging oxygen OγSer113 (HγSer113-OγSer113 = 1.02 Å) and the distance of O3βATP-PγATP is elongated from 1.68 to 2.07 Å in the transition state. It is a concerted mechanism involving a late proton transfer to Asp457. (TIF) [file pone.0072048.s003.tif]

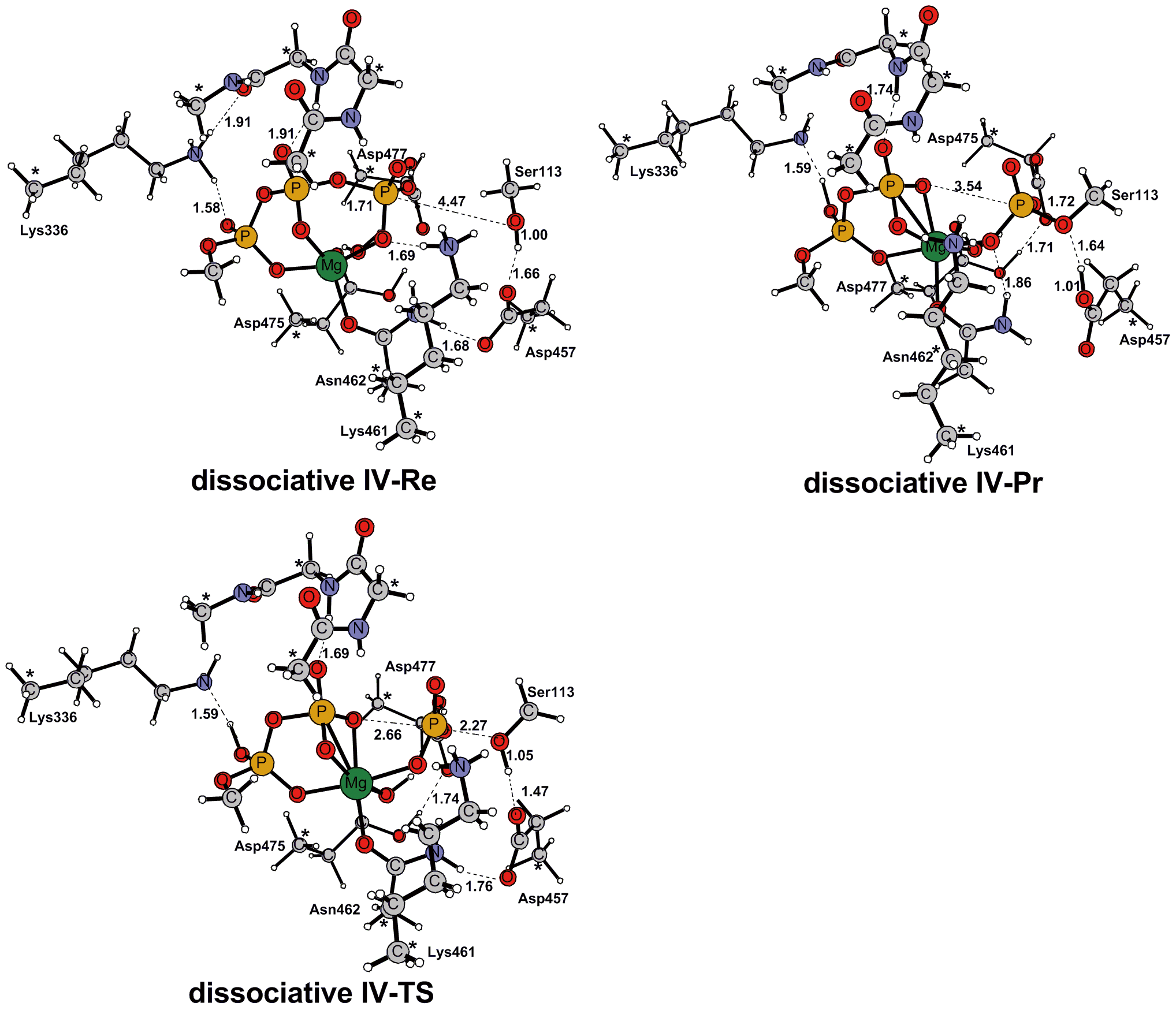

Supplement: Figure S4 — Optimized structures of Re, TS and Pr for the dissociative IV [1 Mg2+|1 H2O|Asp457-H+|Asp477-H+] model. Based on dissociative I model, one of the water molecules which does not coordinate Mg2+ in the product was removed from the active site. The resulting model, dissociative IV, share the same reaction pathway with dissociative I model. However, dissociative IV model results in a 4.3 kcal mol−1 higher TS than dissociative I model. This suggests that the 6-fold coordination of Mg2+ is essential for the phosphorylation reaction. Moreover, water molecules are expected to play an important role in stabilization of the transition state and timing of the phosphotransfer reaction. (TIFF) [file pone.0072048.s004.tiff]
