# Supplementary material for: Unique Kinase Catalytic Mechanism of AceK with a Single Magnesium Ion
Source: PLoS One. 2013 Aug 19;8(8):e72048. doi: 10.1371/journal.pone.0072048 (PMC3747045; doi:10.1371/journal.pone.0072048)
Supplement: Text S1 — Fukui function of Pγ in dissociative and associative reaction. (PDF) [file pone.0072048.s007.pdf]

Conceptual density functional theory (DFT) has played a very important role in effectively handling the understanding of chemical reactivity and site selectivity of molecular systems. The commonly used indices consist of global reactivity descriptors, such as chemical potential, global hardness, global softness, electronegativity, and electrophilicity and so on, and local reactivity descriptors including the Fukui function, local softness, and local philicity and so forth. The global descriptors have been highly successful in predicting global chemical reactivity trends, while the local ones are extensively employed to probe the local reactivity and site selectivity.

For an *N*-electron system with total energy *E*, electronegativity and hardness have been defined as the following first- and second-order derivatives:

$$\chi = -\mu = -\left(\frac{\partial E}{\partial N}\right)_{v(\vec{r})} \qquad (equation1)$$

$$\eta = (1/2)\left(\frac{\partial^2 E}{\partial N^2}\right)_{v(\vec{r})} = (1/2)\left(\frac{\partial \mu}{\partial N}\right)_{v(\vec{r})} \qquad (equation2)$$

where μ and $v(\vec{r})$ are chemical and external potentials, respectively. In the finite difference approximations, the above quantities can be rewritten as follows:

$$\chi = 1/2(I + A) \qquad (equation3)$$

$$\eta = 1/2(I - A) \qquad (equation4)$$

where *I* is the ionization energy and *A* is the electron affinity. The global softness *S* can be naturally defined as:

$$S = \left(\frac{\partial N}{\partial \mu}\right)_{v(\vec{r})} = \frac{1}{2\eta} \qquad (equation5)$$

On the basis of a previous idea by Maynard *et al.*, Parr *et al.* have introduced the concept of electrophilicity which reflects the stabilization in energy when the system acquires an additional electronic charge from the environment. The electrophilicity index is defined as:

$$\omega = \frac{\mu^2}{2\eta} \qquad (equation6)$$

In order to define the reactivity/selectivity of a specific site in a molecule, local quantities have to be employed. One of the widely used local descriptors is Fukui function (FF) or the frontier function, which forms the background of local reactivity/selectivity theories in the spirit of Fukui's frontier orbital

theory and is defined as the derivative of the electron density $\rho(\vec{r})$ with respect to the total number of electrons *N* in the system, at constant external potential $v(\vec{r})$ acting on an electron due to all the nuclei in the system:

$$f(\vec{r}) = \left(\frac{\partial \rho(\vec{r})}{\partial N}\right)_{v(\vec{r})} = \left(\frac{\delta \mu}{\delta v(\vec{r})}\right)_N \qquad (equation 7)$$

For a finite system, such as an atom or molecule, discontinuities in the derivative of $\rho(\vec{r})$ versus *N* in eqn. (7) provide three types of Fukui functions depending on the direction of the electron transfer. In finite difference approximations they are given by:

$$f^+(\vec{r}) = \rho_{N+1}(\vec{r}) - \rho_N(\vec{r}) \text{ for nucleophilic attack,} \qquad (equation 8)$$

$$f^-(\vec{r}) = \rho_N(\vec{r}) - \rho_{N-1}(\vec{r}) \text{ for electrophilic attack,} \qquad (equation 9)$$

$$f^0(\vec{r}) = [\rho_{N+1}(\vec{r}) - \rho_{N-1}(\vec{r})]/2 \text{ for radical attack,} \qquad (equation 10)$$

The condensed Fukui function is similarly found by taking the finite difference approximations from Mulliken/NBO population analysis of atoms in molecules:

$$f_k^+ = q_k(N+1) - q_k(N) \text{ for nucleophilic attack,} \qquad (equation 11)$$

$$f_k^- = q_k(N) - q_k(N-1) \text{ for electrophilic attack,} \qquad (equation 12)$$

$$f_k^0 = (q_k(N+1) - q_k(N-1))/2 \text{ for radical attack,} \qquad (equation 13)$$

where q_k is the gross charge of atom *k* in a molecule.

The local softness of a species is proportional to the Fukui function and defined as follows:

$$s(\vec{r}) = \left(\frac{\partial \rho(\vec{r})}{\partial \mu}\right)_{v(\vec{r})} = \left(\frac{\partial \rho(\vec{r})}{\partial N}\right)_{v(\vec{r})} \left(\frac{\partial N}{\partial \mu}\right)_{v(\vec{r})} = f(\vec{r})S \qquad (equation 14)$$

Therefore, three different types of local softness associated with the corresponding Fukui function (eqn. 11, 12, and 13) can be defined as follows:

$$s^\alpha(\vec{r}) = f^\alpha(\vec{r})S \qquad (equation 15)$$

Related condensed-to-atom quantities are given as:

$$s_k^\alpha(\vec{r}) = f_k^\alpha(\vec{r})S \qquad (equation 16)$$

Chattaraj *et al.* have introduced the concept of local philicity, which is supposed to contain

information about almost all of the known global and local descriptors of chemical reactivity and selectivity. This local philicity index is related to Fukui function as follows:

$$\omega(\vec{r}) = \omega f(\vec{r}) \qquad (equation17)$$

Thus, three different types of $\omega(\vec{r})$ can be readily defined as

$$\omega^{\alpha}(\vec{r}) = \omega f^{\alpha}(\vec{r}) \qquad (equation18)$$

where again α = +, -, 0 refer to nucleophilic, electrophilic, and radical attacks, respectively. Related condensed-to-atom variants for the atomic site *k* can be written as

$$\omega_k^{\alpha}(\vec{r}) = \omega f_k^{\alpha}(\vec{r}) \qquad (equation19)$$

The conceptual DFT indices were calculated according to the following equations and shown in Table S1. As shown the table, the fk^+ (**P_γ**) in the Asp-Re is twice as large as that in the ATP-Re，indicating the P_γ site is more susceptible in the Asp-Re for nucleophilic attack. The difference becomes much more apparent in the TS, reflecting the already broke P_γ–O_{ATP} bond.
