# Supplementary material for: Unique Kinase Catalytic Mechanism of AceK with a Single Magnesium Ion
Source: PLoS One. 2013 Aug 19;8(8):e72048. doi: 10.1371/journal.pone.0072048 (PMC3747045; doi:10.1371/journal.pone.0072048)
Supplement: Text S2 — Cartesian coordinates for all structures. (PDF) [file pone.0072048.s008.pdf]

dissociative I-Re

C1	-63.97900003	-10.52500003	-15.92200006
H2	-64.96518651	-10.44888135	-16.38488509
H3	-63.42602621	-9.59902485	-16.07476781
C4	-63.16530357	-11.68867642	-16.45748697
O5	-61.98883443	-11.87181979	-16.12767653
N6	-63.81962648	-12.52497206	-17.31413792
H7	-64.70375611	-12.22338478	-17.74215217
C8	-63.11800104	-13.63199983	-17.92900122
H9	-62.71308189	-14.31321029	-17.17527516
C10	-61.90944522	-13.23630449	-18.81631691
O11	-61.00947094	-14.03878753	-19.04253743
N12	-61.93415043	-11.97330582	-19.34013499
H13	-62.75551457	-11.39713099	-19.19155631
C14	-60.88000010	-11.46799999	-20.20299988
H15	-60.00493359	-12.10740252	-20.04526949
C16	-60.51293148	-9.99476933	-19.93527878
O17	-60.20245974	-9.23957960	-20.86531667
N18	-60.49820202	-9.59602523	-18.64413671
H19	-60.88748511	-10.20439304	-17.93119726
C20	-60.17200002	-8.21800001	-18.30200000
H21	-59.19236547	-7.95919619	-18.71929296
C22	-59.06100002	-2.38000003	-17.01000002
H23	-59.39256983	-1.45609205	-17.50071608
C24	-59.41674113	-3.61463811	-17.85375206
H25	-59.11284940	-4.52486873	-17.31772387
H26	-60.50812742	-3.67075162	-17.95047756
C27	-58.76794156	-3.61075909	-19.25055777
H28	-59.06506154	-2.69260924	-19.78052042
H29	-57.67625756	-3.55257358	-19.13508013
C30	-59.10878197	-4.83062547	-20.13530389
H31	-58.54102060	-4.75810853	-21.07453082
H32	-58.77790250	-5.75521443	-19.64366784
C33	-60.60724428	-4.92063454	-20.44850281
H34	-61.17723359	-5.06613907	-19.52957553
H35	-60.95127234	-3.96688077	-20.86977352
N36	-61.04986605	-5.98204252	-21.38166559
H37	-60.83935646	-6.92542644	-21.03417986
H38	-62.57544872	-5.73426973	-21.48658469
H39	-60.57919051	-5.89718391	-22.28240467
C40	-71.94099893	-10.93600004	-25.10799898
H41	-70.98242731	-10.41474747	-25.21981911
C42	-72.56746497	-10.64325737	-23.73949931
H43	-72.73272291	-9.56966723	-23.59863372

H44	-73.54866101	-11.13561277	-23.67536223
C45	-71.70203557	-11.17884545	-22.58313465
O46	-71.43829776	-10.38560368	-21.62513140
O47	-71.32781586	-12.37792043	-22.68172716
C48	-73.16099997	-7.76700001	-16.52299997
H49	-73.19409280	-6.87304927	-15.88842113
C50	-71.74974574	-8.35968061	-16.58583348
H51	-71.05238402	-7.60710341	-16.97964552
H52	-71.39806121	-8.59404088	-15.57104784
C53	-71.67903096	-9.61754866	-17.46155732
H54	-72.27991512	-10.41912371	-17.00600631
H55	-72.14453126	-9.40585476	-18.43453111
C56	-70.24427064	-10.10414727	-17.70632327
H57	-69.64849286	-9.27125003	-18.10059664
H58	-69.76784002	-10.39765458	-16.76023509
C59	-70.21023702	-11.25612620	-18.71121734
H60	-70.55730969	-12.19813297	-18.27387823
H61	-70.82967082	-11.03212818	-19.58774421
N62	-68.84068438	-11.48593996	-19.25979593
H63	-68.14264098	-11.87349052	-18.61037964
H64	-68.91119339	-12.13622552	-20.10612102
H65	-68.40051278	-10.58517618	-19.53302641
C66	-71.18424589	-7.09542100	-20.63304200
H67	-71.82938622	-7.90368157	-20.98781394
H68	-71.35668174	-6.19722298	-21.23740637
C69	-69.72159565	-7.47420554	-20.74080425
O70	-68.85061742	-6.75644774	-20.17855154
N71	-69.41967424	-8.54343670	-21.46807210
H72	-68.43427900	-8.75466118	-21.61722058
H73	-70.15531113	-9.21762192	-21.74280204
C74	-67.38499999	-5.18800000	-22.72699997
H75	-67.71312523	-5.79949732	-21.89122040
C76	-68.00730395	-5.66471578	-24.04826898
H77	-69.09987517	-5.70138865	-24.00634634
H78	-67.72800379	-4.98825076	-24.86793088
C79	-67.45603117	-7.03355591	-24.37001316
O80	-66.26349497	-7.28844970	-24.23688534
O81	-68.35614934	-7.93005597	-24.76083128
C82	-64.66400002	-8.97600007	-26.19599990
H83	-64.26866539	-8.99468570	-27.21879487
C84	-65.41894610	-10.29297320	-25.91561454
H85	-66.29037807	-10.38019517	-26.57233115
H86	-64.75521692	-11.14232978	-26.11393216
C87	-65.90876042	-10.41153977	-24.48225139

O88	-64.95910443	-10.77948156	-23.64482122
O89	-67.07387405	-10.19507035	-24.14280376
C90	-68.20913430	-13.23072676	-22.29231058
H91	-68.38103069	-14.05778465	-22.99632181
H92	-67.25880334	-13.40169968	-21.77639675
O93	-69.23956741	-13.18033651	-21.31062379
H94	-70.08878891	-12.87816859	-21.77286837
O95	-67.01971046	-9.33173507	-19.33406883
P96	-65.83344304	-10.31185940	-19.59571563
O97	-65.50717394	-10.36892957	-21.09444280
O98	-66.00065217	-11.62264699	-18.85309148
O99	-64.43322907	-9.57746857	-18.91733056
P100	-64.18013079	-8.09676277	-18.34244091
O101	-65.50695825	-7.45919284	-17.94802616
O102	-63.00417461	-8.01849624	-17.43547697
O103	-63.77581188	-7.25327957	-19.76358043
P104	-64.28401545	-5.76005342	-20.07209788
O105	-63.60246755	-5.42441882	-21.43616213
O106	-65.78306989	-5.60611121	-20.01155541
O107	-63.54522396	-4.76517858	-19.03450671
C108	-63.95091429	-4.62979431	-17.66148034
H109	-63.46779989	-3.72053686	-17.29500303
H110	-63.61929872	-5.49477773	-17.08242142
O111	-67.81179828	-6.41343286	-17.69593838
H112	-66.94214380	-6.57760782	-17.27131500
Mg113	-66.95368373	-7.31231886	-19.54166155
H114	-65.03647660	-4.52255187	-17.59102827
H115	-67.84767567	-5.46646514	-17.90370534
H116	-59.53788888	-2.42458958	-16.02407573
H117	-57.97743126	-2.30171253	-16.85398663
H118	-73.87996812	-8.49003393	-16.11671713
H119	-73.51261036	-7.48006648	-17.52197394
H120	-71.41856434	-6.84601615	-19.59415464
H121	-72.59518374	-10.61415931	-25.92867395
H122	-71.75289410	-12.00873829	-25.20618334
H123	-66.29538447	-5.21039789	-22.78272900
H124	-67.68771327	-4.15450764	-22.51714976
H125	-63.82209807	-8.86360497	-25.50563916
H126	-65.31084744	-8.10958991	-26.07556181
H127	-63.83092419	-14.18433354	-18.54976264
H128	-64.10807453	-10.67130507	-14.84108871
H129	-61.15135726	-11.52514373	-21.26372707
H130	-60.93242105	-7.52732170	-18.67275601
H131	-60.14539654	-8.12949224	-17.21495588

H132	-68.13067227	-12.29561772	-22.86181610
O133	-66.44073673	-7.95571271	-21.53455354
H134	-66.03990096	-8.87607010	-21.52286964
H135	-66.09034791	-7.49707881	-22.31942912
H136	-65.25871158	-10.74050606	-22.68259695
H137	-67.91641528	-8.82948321	-24.73506966

dissociative I-TS

C1	-63.97900000	-10.52500000	-15.92200000
H2	-64.97291000	-10.43065800	-16.36866200
H3	-63.39667000	-9.64649800	-16.20973100
C4	-63.25226600	-11.78666400	-16.34340200
O5	-62.18839400	-12.12845300	-15.82498900
N6	-63.84800700	-12.53324200	-17.32707600
H7	-64.59900800	-12.12774900	-17.87941500
C8	-63.11800100	-13.63200000	-17.92900100
H9	-62.70890600	-14.28100900	-17.15243000
C10	-61.91505700	-13.22565200	-18.82237700
O11	-61.04962100	-14.05821600	-19.08929400
N12	-61.90488600	-11.94327500	-19.28577400
H13	-62.72009700	-11.32819100	-19.17124000
C14	-60.88000000	-11.46800000	-20.20300000
H15	-60.00799600	-12.12124400	-20.09291100
C16	-60.47013500	-10.00318200	-19.96077700
O17	-60.13709600	-9.27795200	-20.90371600
N18	-60.42864200	-9.60620500	-18.66460900
H19	-60.90940300	-10.18864000	-17.98912600
C20	-60.17200000	-8.21800000	-18.30200000
H21	-59.50121200	-7.79637800	-19.05326100
C22	-59.06100000	-2.38000000	-17.01000000
H23	-59.40402200	-1.42722300	-17.43332900
C24	-59.53372000	-3.56734400	-17.86128500
H25	-59.20467700	-4.50620700	-17.39443800
H26	-60.63058000	-3.59576100	-17.85693000
C27	-59.01670900	-3.51751700	-19.30887200
H28	-59.36435800	-2.58655500	-19.78315700
H29	-57.91899900	-3.45491600	-19.28922400
C30	-59.42624200	-4.71497600	-20.18887300
H31	-58.90867500	-4.63641400	-21.15669300
H32	-59.07895100	-5.64965100	-19.72915300
C33	-60.93750600	-4.79923300	-20.44151700
H34	-61.48174600	-4.96376600	-19.50827500
H35	-61.29473400	-3.84010800	-20.84014600
N36	-61.38400200	-5.85106000	-21.37933000
H37	-61.19065500	-6.79015100	-21.01912300

H38	-62.98910600	-5.62860100	-21.48169600
H39	-60.88789300	-5.77760500	-22.26718300
C40	-71.94099900	-10.93600000	-25.10799900
H41	-72.22761700	-9.93470500	-24.76650400
C42	-72.07220700	-11.96169000	-23.96484000
H43	-73.10916900	-11.99726900	-23.61262200
H44	-71.79519500	-12.95424900	-24.33843300
C45	-71.16112900	-11.61682200	-22.77381400
O46	-71.66115300	-10.95316900	-21.82229000
O47	-69.95671300	-12.00959400	-22.87510200
C48	-73.16100000	-7.76700000	-16.52300000
H49	-73.16985100	-6.94384100	-15.79884200
C50	-71.89821300	-8.61761500	-16.39107900
H51	-71.01106100	-7.99137000	-16.55482400
H52	-71.81263800	-9.00380100	-15.36546700
C53	-71.86889000	-9.78595700	-17.38372000
H54	-72.67209500	-10.49928900	-17.14723800
H55	-72.08120900	-9.40871000	-18.39363400
C56	-70.50728000	-10.48650300	-17.40188900
H57	-69.73462100	-9.73137500	-17.59670700
H58	-70.28620400	-10.91180900	-16.41174400
C59	-70.41709600	-11.57501700	-18.46838400
H60	-70.95951800	-12.48446600	-18.19088300
H61	-70.80107200	-11.23794700	-19.43730900
N62	-68.98436300	-11.94073100	-18.69691400
H63	-68.53948300	-12.42393100	-17.91335800
H64	-68.83125400	-12.49645900	-19.56945500
H65	-68.40543200	-11.08409300	-18.89972600
C66	-71.18424600	-7.09542100	-20.63304200
H67	-72.00234700	-7.72611400	-20.99046500
H68	-71.10945300	-6.20457500	-21.26497900
C69	-69.84950300	-7.81892400	-20.64712200
O70	-68.82360800	-7.22229300	-20.23344200
N71	-69.81540100	-9.06661000	-21.09709300
H72	-68.91135600	-9.53538600	-21.09828000
H73	-70.62590800	-9.62606800	-21.41680400
C74	-67.38500000	-5.18800000	-22.72700000
H75	-67.64672200	-5.76101600	-21.83852400
C76	-68.15154600	-5.68036000	-23.96279400
H77	-69.23560300	-5.67454900	-23.81224500
H78	-67.93746900	-5.03647200	-24.82769600
C79	-67.69038300	-7.07525300	-24.31306400
O80	-66.50322700	-7.38353000	-24.28543600
O81	-68.65834600	-7.93095100	-24.62321600

C82	-64.66400000	-8.97600000	-26.19600000
H83	-64.34838900	-8.68000200	-27.20371100
C84	-65.83412300	-9.97278500	-26.28829700
H85	-66.70139500	-9.51983000	-26.77692900
H86	-65.52969900	-10.84590400	-26.88045700
C87	-66.27509400	-10.47332900	-24.92841000
O88	-65.31301500	-11.13465900	-24.29714400
O89	-67.39142100	-10.29834900	-24.44781300
C90	-67.63587800	-13.64840800	-21.85939600
H91	-68.47757600	-14.29972200	-22.11420600
H92	-66.96345300	-14.14322200	-21.15653400
O93	-68.13006400	-12.45734300	-21.22529600
H94	-68.88625800	-12.08536600	-21.86678000
O95	-67.34052800	-10.13708500	-19.90619900
P96	-66.40540600	-11.27509700	-20.36737700
O97	-65.67674600	-11.06983200	-21.66332000
O98	-66.13291600	-12.36151100	-19.38079900
O99	-63.91304700	-9.89948100	-19.26721800
P100	-64.10221400	-8.55228500	-18.59601000
O101	-65.61140100	-8.27030000	-18.29447100
O102	-63.15859800	-8.06962400	-17.52308600
O103	-63.84303400	-7.38805200	-19.89605500
P104	-64.57996900	-5.99947500	-20.04241200
O105	-64.00328600	-5.39812100	-21.38089900
O106	-66.09073600	-6.08468500	-19.99502600
O107	-64.02258700	-4.96704200	-18.92868600
C108	-64.40330000	-5.09763500	-17.54423900
H109	-64.00334900	-4.21316600	-17.04091000
H110	-63.97515300	-6.01230700	-17.12482400
O111	-68.03052300	-7.96358100	-17.64928000
H112	-67.07170600	-8.10998300	-17.43396400
Mg113	-66.93191900	-7.98389600	-19.77715100
H114	-65.49430600	-5.10578000	-17.45283100
H115	-68.16791100	-7.00636200	-17.57290000
H116	-59.44509000	-2.44741100	-15.98560100
H117	-57.96544300	-2.34141100	-16.95461300
H118	-74.06580200	-8.36552600	-16.35645300
H119	-73.23797000	-7.32874100	-17.52576500
H120	-71.39475100	-6.75687300	-19.61351300
H121	-72.58358600	-11.20506100	-25.95494400
H122	-70.90489800	-10.88812100	-25.45972000
H123	-66.30696600	-5.26156300	-22.87987300
H124	-67.62716000	-4.13774600	-22.52425800
H125	-63.80928200	-9.43828300	-25.69332500

H126	-64.95795200	-8.08600500	-25.64185800
H127	-63.81374000	-14.20998300	-18.54552300
H128	-64.07361800	-10.53376900	-14.82948600
H129	-61.21067000	-11.52108500	-21.24688600
H130	-61.10866600	-7.65596100	-18.24019500
H131	-59.67946400	-8.18897100	-17.32375000
H132	-67.08920700	-13.39035400	-22.77142000
O133	-66.32905500	-8.39508500	-21.71879900
H134	-65.95043000	-9.28753000	-21.88607500
H135	-66.08856100	-7.81752100	-22.46568600
H136	-65.55739300	-11.26663300	-23.33832600
H137	-68.24859600	-8.84388200	-24.68822400

dissociative I-Pr

C1	-63.97900000	-10.52500002	-15.92200000
H2	-64.87730015	-10.35030071	-16.50838865
H3	-63.36388959	-9.62771932	-15.98633043
C4	-63.21514170	-11.74385416	-16.40166483
O5	-62.11168945	-12.04063957	-15.94142799
N6	-63.84152571	-12.52059928	-17.34164168
H7	-64.62700267	-12.14991586	-17.87981282
C8	-63.11800099	-13.63199995	-17.92900106
H9	-62.68203193	-14.25753260	-17.14730916
C10	-61.94386569	-13.22830490	-18.85880684
O11	-61.10404294	-14.06758154	-19.17869349
N12	-61.91539982	-11.93482674	-19.29253986
H13	-62.68426448	-11.29154575	-19.09560802
C14	-60.88000011	-11.46799992	-20.20299988
H15	-60.00692143	-12.11546219	-20.07016532
C16	-60.49106586	-9.99388187	-19.96722685
O17	-60.23199569	-9.25010227	-20.91764235
N18	-60.38258607	-9.61263450	-18.66775778
H19	-60.82237066	-10.21457135	-17.98040864
C20	-60.17200000	-8.21800002	-18.30200004
H21	-59.72650343	-7.71707175	-19.16171750
C22	-59.06099999	-2.38000002	-17.01000001
H23	-59.50555826	-1.45726416	-17.40419120
C24	-59.49677491	-3.60060346	-17.83424213
H25	-59.06917803	-4.51226193	-17.39343961
H26	-60.58584542	-3.71097085	-17.76027172
C27	-59.07821038	-3.50930022	-19.31142100
H28	-59.51093553	-2.59745973	-19.75143597
H29	-57.98715723	-3.38086977	-19.36281564
C30	-59.47451750	-4.72024461	-20.17952570
H31	-59.03975446	-4.59015469	-21.18211490

H32	-59.02928287	-5.63637420	-19.76885565
C33	-60.99357349	-4.89753026	-20.32048623
H34	-61.44768040	-5.16811184	-19.36335733
H35	-61.44399560	-3.94054851	-20.61585603
N36	-61.44537054	-5.90959264	-21.29806990
H37	-61.24224653	-6.86463659	-20.98983736
H38	-63.02251120	-5.64951497	-21.38524931
H39	-60.97301454	-5.78498524	-22.19300895
C40	-71.94099902	-10.93599997	-25.10799897
H41	-72.47277524	-10.17511269	-24.52820660
C42	-71.70643074	-12.19446003	-24.25400620
H43	-72.66839136	-12.60340392	-23.92493879
H44	-71.17819405	-12.96034998	-24.82823795
C45	-70.90520629	-11.83662806	-23.01583341
O46	-71.38344582	-11.18641836	-22.09487839
O47	-69.65249345	-12.26736182	-23.08161724
C48	-73.16099984	-7.76700013	-16.52300008
H49	-73.66085136	-6.91024789	-16.05543184
C50	-71.66583152	-7.80319540	-16.17147681
H51	-71.18625053	-6.86939206	-16.49432729
H52	-71.54618434	-7.84370648	-15.07941668
C53	-70.93419297	-8.99113947	-16.81210889
H54	-71.39193669	-9.93017117	-16.46393294
H55	-71.09490178	-8.96438996	-17.89957649
C56	-69.41890864	-9.01927613	-16.54133032
H57	-68.96604920	-8.06678764	-16.84844959
H58	-69.23356310	-9.12862502	-15.46193207
C59	-68.77441218	-10.17417844	-17.30909662
H60	-69.15328546	-11.14348929	-16.96475867
H61	-68.98053523	-10.09434290	-18.37627514
N62	-67.28480960	-10.22574495	-17.20576743
H63	-66.97453462	-10.40828922	-16.24975797
H64	-66.89224288	-10.98062138	-17.86478536
H65	-66.79024067	-9.34739170	-17.54254539
C66	-71.18424601	-7.09542097	-20.63304207
H67	-72.00912596	-7.71301649	-20.99741023
H68	-71.06372360	-6.22224813	-21.28144801
C69	-69.87172314	-7.86630981	-20.57531491
O70	-68.79473799	-7.27807463	-20.28338150
N71	-69.92014341	-9.17104392	-20.80925912
H72	-69.02726241	-9.68494267	-20.72684038
H73	-70.73555607	-9.67117904	-21.15012107
C74	-67.38499999	-5.18799992	-22.72700000
H75	-67.81360504	-5.68304074	-21.85558732

C76	-67.99312659	-5.72583580	-24.03040691
H77	-69.08448544	-5.64416993	-24.05002094
H78	-67.60530201	-5.16441294	-24.89209853
C79	-67.58440174	-7.16728650	-24.21512759
O80	-66.42483458	-7.53602555	-24.05118359
O81	-68.56728769	-7.99555106	-24.55292417
C82	-64.66400006	-8.97600009	-26.19599989
H83	-64.39668149	-8.76040018	-27.23742202
C84	-65.79992307	-10.01779028	-26.15590412
H85	-66.69147703	-9.64379095	-26.66864455
H86	-65.47694156	-10.92626882	-26.68315556
C87	-66.22759333	-10.44869032	-24.75888073
O88	-65.24676250	-10.94587209	-24.04144449
O89	-67.39378761	-10.37242164	-24.35758545
C90	-67.56340383	-13.60484931	-21.08808407
H91	-68.44007342	-14.11701203	-21.49376341
H92	-67.32818753	-13.99239238	-20.09255708
O93	-67.88465952	-12.20998905	-21.00962071
H94	-69.09369129	-11.99073059	-22.30179062
O95	-67.42704823	-9.89349363	-20.16472934
P96	-66.65509435	-11.22834599	-20.38913983
O97	-65.58253264	-11.11375893	-21.47763302
O98	-66.24504213	-11.88032569	-19.06118562
O99	-63.61786163	-9.38950207	-18.75904163
P100	-64.23817202	-8.14613953	-18.17863615
O101	-65.84020443	-8.15537563	-18.28549062
O102	-63.75210683	-7.55484246	-16.88336011
O103	-63.84619948	-6.89601825	-19.35689839
P104	-64.68043528	-5.70303041	-19.94465530
O105	-64.02246883	-5.34084747	-21.32749586
O106	-66.17138763	-5.97766989	-20.03495356
O107	-64.37504784	-4.39718270	-19.05041910
C108	-64.60320823	-4.44118836	-17.62168191
H109	-64.25049760	-3.48129142	-17.23627798
H110	-64.04903682	-5.27128378	-17.17120630
O111	-67.58805279	-6.14611709	-17.37395301
H112	-66.83829850	-6.77686738	-17.45470816
Mg113	-66.76815881	-7.96012343	-20.06603720
H114	-65.66901578	-4.56209474	-17.40622919
H115	-67.59999575	-5.76001285	-18.26629764
H116	-59.36965262	-2.47773075	-15.96270271
H117	-57.97031842	-2.25619194	-17.02761761
H118	-73.67199952	-8.67729688	-16.18401661
H119	-73.31100497	-7.68974726	-17.60728812

H120	-71.42157233	-6.72807350	-19.62872214
H121	-72.53826665	-11.17687383	-25.99438750
H122	-70.98970771	-10.50773460	-25.44182865
H123	-66.30500214	-5.33575883	-22.71064761
H124	-67.58471321	-4.11386787	-22.63078383
H125	-63.77539166	-9.36037589	-25.68641106
H126	-64.96563626	-8.04920299	-25.70734036
H127	-63.82001246	-14.23453819	-18.51383873
H128	-64.24855417	-10.67813571	-14.86770481
H129	-61.19431863	-11.53531289	-21.25083732
H130	-61.11826970	-7.73571832	-18.02779660
H131	-59.48264503	-8.15538802	-17.45198508
H132	-66.70792285	-13.76960806	-21.75383643
O133	-65.46641432	-8.37025643	-21.59452358
H134	-65.32794648	-9.35511887	-21.61079370
H135	-65.66087863	-8.07635881	-22.50711932
H136	-65.52194103	-11.10812469	-23.06860391
H137	-68.18802200	-8.92847613	-24.57150157

dissociative II-Re

C1	-63.97900000	-10.52500000	-15.92200000
H2	-64.90819245	-10.35424705	-16.34259536
H3	-63.24341681	-9.81207563	-16.30172970
C4	-63.39831569	-11.93654944	-16.16091549
O5	-62.61717960	-12.52814436	-15.41297480
N6	-63.82662672	-12.49921433	-17.34810309
H7	-64.43672759	-11.91891544	-17.92364618
C8	-63.11800000	-13.63200000	-17.92900000
H9	-62.64941315	-14.17967114	-17.10983497
C10	-61.98493092	-13.26577859	-18.92213094
O11	-61.18364769	-14.14178703	-19.26230191
N12	-61.95379022	-11.98305387	-19.36217732
H13	-62.70226745	-11.31792699	-19.14685561
C14	-60.88000000	-11.46800000	-20.20300000
H15	-60.18511185	-12.28278441	-20.40300824
C16	-60.07877165	-10.31788570	-19.54696791
O17	-58.84190323	-10.34330008	-19.53285804
N18	-60.80987026	-9.31114594	-19.01943469
H19	-61.83763799	-9.27686643	-19.15371782
C20	-60.17200000	-8.21800000	-18.30200000
H21	-59.64380641	-7.53342647	-19.00078145
C22	-59.06100100	-2.38000000	-17.01000100
H23	-59.60562750	-1.42686678	-17.04549854
C24	-59.95963430	-3.54871327	-17.43274369
H25	-59.40065901	-4.49081944	-17.34836241

H26	-60.79550992	-3.63540527	-16.72810848
C27	-60.49480902	-3.41364415	-18.86652424
H28	-61.02825858	-2.45259989	-18.96117268
H29	-59.64340090	-3.33563910	-19.55962976
C30	-61.39482289	-4.57876868	-19.32501895
H31	-61.62675418	-4.46617921	-20.39221286
H32	-60.83733858	-5.51761749	-19.22675112
C33	-62.71338118	-4.74184010	-18.56376477
H34	-63.24149564	-5.63371097	-18.90337277
H35	-62.58425354	-4.85589176	-17.48434605
N36	-63.63043176	-3.59193925	-18.82906903
H37	-64.01586689	-3.69037008	-19.84179747
H38	-64.45687530	-3.56515015	-18.15617426
H39	-63.14557327	-2.69927471	-18.72826039
C40	-71.94100000	-10.93600000	-25.10800000
H41	-71.68175560	-11.95258354	-25.44046831
C42	-70.98310235	-10.45864986	-24.04188422
H43	-69.94986218	-10.36254519	-24.43234620
H44	-71.26221504	-9.46305786	-23.67715222
C45	-70.90875624	-11.40975368	-22.86787413
O46	-70.63531501	-10.75732619	-21.72548439
O47	-71.05797151	-12.61777445	-22.93209795
C48	-73.16100000	-7.76700000	-16.52300000
H49	-73.52264093	-6.78789762	-16.81925900
C50	-72.09159572	-8.28017124	-17.50252217
H51	-72.53986668	-8.31228840	-18.50347169
H52	-71.27203846	-7.55261598	-17.55574928
C53	-71.48916992	-9.66513308	-17.20815700
H54	-70.96447171	-9.65070963	-16.24226956
H55	-72.29550612	-10.41269104	-17.12263574
C56	-70.52549030	-10.04530939	-18.34256363
H57	-71.06582730	-9.95145308	-19.29215987
H58	-69.70538049	-9.31756001	-18.35933206
C59	-69.92583787	-11.45341477	-18.30471614
H60	-69.20890222	-11.54222555	-17.47437308
H61	-70.71590767	-12.20085961	-18.14023689
N62	-69.30685754	-11.74909271	-19.61574648
H63	-70.23805332	-11.34772517	-20.98488952
H64	-68.52674375	-11.11421806	-19.80768789
H65	-68.90717781	-12.68583688	-19.62696478
C66	-71.18399900	-7.09500000	-20.63300000
H67	-71.69274505	-8.05586221	-20.82639993
H68	-71.35128392	-6.44881013	-21.50472596
C69	-69.64103508	-7.32670198	-20.52886918

O70	-68.94075542	-6.51245396	-19.89108283
N71	-69.14292231	-8.37902836	-21.17376630
H72	-68.15139644	-8.62789882	-21.16474330
H73	-69.73905961	-9.09764272	-21.57670743
C74	-67.38499900	-5.18800100	-22.72700000
H75	-68.03757368	-5.34234650	-21.86368075
C76	-65.94754262	-5.60686725	-22.38371951
H77	-65.90787656	-6.67446891	-22.13573725
H78	-65.27188653	-5.42317560	-23.22555600
C79	-65.45175948	-4.84381763	-21.16153037
O80	-64.42742312	-4.10553890	-21.28799348
O81	-66.11573498	-4.95712596	-20.08771822
C82	-64.66400000	-8.97600000	-26.19599900
H83	-63.63703190	-8.80207142	-25.82731475
C84	-65.58710174	-9.47090674	-25.09531927
H85	-65.18691885	-10.39191623	-24.64830776
H86	-65.62030103	-8.74452009	-24.27210140
C87	-67.05104287	-9.75590312	-25.54276263
O88	-67.34157616	-9.60677119	-26.74589389
O89	-67.83580942	-10.13249046	-24.60344202
C90	-66.13467009	-10.96153104	-21.69220508
H91	-65.99584011	-11.96166847	-22.14210361
H92	-66.01551907	-11.03636669	-20.60862222
O93	-67.42677393	-10.44195009	-21.95611924
H94	-67.51688509	-10.31405020	-22.94728813
O95	-65.59923803	-7.77502814	-19.71716323
P96	-64.82309535	-8.86206432	-18.92146744
O97	-63.59192185	-9.49411474	-19.57893799
O98	-65.79792892	-9.89182438	-18.30735249
O99	-64.09307868	-7.96298865	-17.65971930
P100	-64.60039627	-7.09764831	-16.38512708
O101	-65.74997535	-7.77671230	-15.66405298
O102	-63.40271748	-6.55069622	-15.67207892
O103	-65.33541767	-5.82329239	-17.26275585
P104	-66.35030669	-4.60381898	-16.82863216
O105	-65.63993485	-3.28979493	-17.06794353
O106	-67.62124150	-4.98015479	-17.58380823
O107	-66.61169629	-4.66932783	-15.22314689
C108	-67.60605510	-5.53254364	-14.66299285
H109	-67.84411158	-5.12667761	-13.67141729
H110	-67.20397659	-6.54505521	-14.57721779
O111	-67.60995280	-8.05000295	-17.64016715
H112	-67.09298576	-8.85422212	-17.96204371
Mg113	-66.97774621	-6.45549219	-18.95124392

H114	-68.50792703	-5.53537909	-15.28421197
H115	-67.12578738	-7.89769508	-16.78234904
H116	-58.68897038	-2.51123233	-15.98614262
H117	-58.18953858	-2.28580802	-17.67268678
H118	-72.74942314	-7.68613817	-15.50509362
H119	-74.01362003	-8.45730751	-16.47695832
H120	-71.52930417	-6.62609297	-19.75047084
H121	-71.88508408	-10.28197805	-26.00560676
H122	-72.98244142	-10.95239926	-24.77722377
H123	-67.43254658	-4.13808386	-23.01460450
H124	-67.77391025	-5.79171979	-23.56367187
H125	-65.02856083	-8.04122793	-26.63844686
H126	-64.60559273	-9.70469898	-27.02377383
H127	-63.81681141	-14.30281852	-18.44412706
H128	-63.97917869	-10.38794679	-14.80095925
H129	-61.30114356	-11.10779360	-21.15189420
H130	-60.92633452	-7.67017894	-17.75460458
H131	-59.41291258	-8.61148957	-17.61597062
H132	-65.33335185	-10.30499419	-22.05635045

dissociative II-TS

C1	-63.97900000	-10.52500000	-15.92200000
H2	-65.04743600	-10.47514300	-16.13993000
H3	-63.53635800	-9.74829600	-16.55854900
C4	-63.37080200	-11.87800700	-16.22178600
O5	-62.49671400	-12.40652700	-15.53237600
N6	-63.84842200	-12.50782000	-17.36148200
H7	-64.52334400	-11.99080000	-17.92337700
C8	-63.11800000	-13.63200000	-17.92900000
H9	-62.66369800	-14.18500400	-17.10569400
C10	-61.95743400	-13.25906100	-18.88642100
O11	-61.10126500	-14.10534000	-19.15563700
N12	-61.96949800	-11.99598000	-19.39044600
H13	-62.76626800	-11.37876100	-19.23061900
C14	-60.88000000	-11.46800000	-20.20300000
H15	-60.17212200	-12.27720100	-20.37866600
C16	-60.10129900	-10.30991000	-19.54906800
O17	-58.86590400	-10.31035400	-19.55711800
N18	-60.83240700	-9.31185200	-19.00006300
H19	-61.84866100	-9.27281300	-19.09543100
C20	-60.17200000	-8.21800000	-18.30200000
H21	-59.67061500	-7.53447100	-19.00200900
C22	-59.06100100	-2.38000000	-17.01000100
H23	-59.61014300	-1.43061000	-16.95005900
C24	-59.99467200	-3.53697600	-17.38624300

H25	-59.42493000	-4.47640500	-17.40160800
H26	-60.75527900	-3.66368300	-16.60673300
C27	-60.67212400	-3.34437700	-18.75066200
H28	-61.22175300	-2.38780000	-18.74587700
H29	-59.89282400	-3.22285600	-19.51924700
C30	-61.60375300	-4.49878100	-19.16719000
H31	-61.90144900	-4.37115500	-20.21639100
H32	-61.04201000	-5.43965000	-19.11684900
C33	-62.87243800	-4.67415400	-18.32862400
H34	-63.39368200	-5.58931200	-18.61468200
H35	-62.69180000	-4.77366700	-17.25494800
N36	-63.82886500	-3.54783200	-18.56330200
H37	-64.22442900	-3.64000500	-19.55732900
H38	-64.64965800	-3.54028300	-17.86725300
H39	-63.36680300	-2.64207500	-18.46832800
C40	-71.94100000	-10.93600000	-25.10800000
H41	-71.61440800	-11.93093100	-25.42649100
C42	-70.91957100	-10.32629200	-24.14171600
H43	-69.95955800	-10.12418200	-24.64433900
H44	-71.26678900	-9.35615900	-23.76811900
C45	-70.61064000	-11.22028500	-22.94808200
O46	-70.26266600	-10.49962100	-21.87869400
O47	-70.67360400	-12.43853800	-22.96675400
C48	-73.16100000	-7.76700000	-16.52300000
H49	-73.57571000	-6.79128100	-16.80529600
C50	-72.11240600	-8.22834200	-17.53612500
H51	-72.58662100	-8.26715400	-18.52495600
H52	-71.32058500	-7.47227700	-17.60426400
C53	-71.45138300	-9.59376300	-17.27645800
H54	-70.96347300	-9.59529300	-16.29173700
H55	-72.22146100	-10.38319200	-17.25355800
C56	-70.41964300	-9.87710300	-18.38130200
H57	-70.90361600	-9.72282600	-19.35275900
H58	-69.61751100	-9.13541700	-18.29299400
C59	-69.80121900	-11.27584800	-18.41173200
H60	-69.18142800	-11.44077600	-17.51779000
H61	-70.59420800	-12.03803700	-18.41147800
N62	-69.01786800	-11.44590800	-19.65928900
H63	-69.83127600	-11.02521700	-21.09919800
H64	-68.16581500	-10.87191800	-19.61152000
H65	-68.67707900	-12.40497200	-19.72057900
C66	-71.18399900	-7.09500000	-20.63300000
H67	-71.63284900	-8.07396900	-20.82157500
H68	-71.34268900	-6.47302800	-21.52305500

C69	-69.66286500	-7.21210700	-20.37860900
O70	-69.11573800	-6.52275500	-19.49720600
N71	-68.95667400	-8.03307900	-21.16729900
H72	-67.95985500	-8.13519200	-20.96621400
H73	-69.37439400	-8.72666300	-21.77958600
C74	-67.38499900	-5.18800100	-22.72700000
H75	-68.08333200	-5.29944700	-21.89322100
C76	-65.97939500	-5.60986500	-22.28156500
H77	-65.96867200	-6.68381200	-22.06676800
H78	-65.24298700	-5.40143800	-23.06532900
C79	-65.57378600	-4.87787600	-21.00230500
O80	-64.62149100	-4.04638600	-21.07225000
O81	-66.24541000	-5.11404200	-19.95447800
C82	-64.66400000	-8.97600000	-26.19599900
H83	-63.59463000	-8.73140600	-26.13329500
C84	-65.28245100	-9.13548000	-24.79917300
H85	-64.76130600	-9.92560100	-24.24098800
H86	-65.12472500	-8.22195500	-24.21022500
C87	-66.80710200	-9.44984800	-24.76580700
O88	-67.43412800	-9.49560400	-25.84281800
O89	-67.30535200	-9.62922000	-23.59882600
C90	-65.81741500	-11.64631300	-21.59720500
H91	-66.32508200	-12.07874200	-22.46907400
H92	-66.00532400	-12.27720700	-20.71975400
O93	-66.32452000	-10.35245400	-21.33737900
H94	-66.58186100	-9.92897200	-22.23532800
O95	-66.08723900	-8.03491800	-19.88245000
P96	-65.38413300	-9.33763900	-19.50584600
O97	-63.98084400	-9.69975200	-19.91024300
O98	-66.15253100	-10.27370600	-18.58129600
O99	-64.06932600	-7.83860600	-17.64339600
P100	-64.48356000	-7.10053100	-16.35902400
O101	-65.57322300	-7.82411200	-15.53621200
O102	-63.41097800	-6.34109300	-15.61351000
O103	-65.51276400	-5.81533200	-17.14012900
P104	-66.49139300	-4.63326800	-16.61191100
O105	-65.82624600	-3.28285200	-16.82065700
O106	-67.79398900	-4.98319100	-17.34173300
O107	-66.71081500	-4.73673700	-14.99820400
C108	-67.54907800	-5.74883600	-14.43439700
H109	-67.78455100	-5.42245000	-13.41204600
H110	-67.01317100	-6.70223800	-14.42334300
O111	-67.46426300	-8.09877700	-17.31802700
H112	-67.12804900	-8.92965200	-17.72706400

Mg113	-67.02982600	-6.48282600	-18.63420900
H114	-68.47864900	-5.84336000	-15.00775200
H115	-66.80201700	-7.98642200	-16.53668100
H116	-58.58341100	-2.54816600	-16.03641200
H117	-58.26400200	-2.25129200	-17.75564700
H118	-72.73014800	-7.66307100	-15.51875800
H119	-73.99679700	-8.47822800	-16.45128900
H120	-71.64760600	-6.61014700	-19.77650200
H121	-72.05717100	-10.30536800	-25.99720400
H122	-72.92559100	-11.04679600	-24.63488100
H123	-67.39921400	-4.13464700	-23.04104700
H124	-67.73867500	-5.80225600	-23.56339400
H125	-65.17381700	-8.18268000	-26.75358500
H126	-64.77722800	-9.89681200	-26.78073900
H127	-63.80395900	-14.29863500	-18.46621600
H128	-63.79898200	-10.26752200	-14.87748400
H129	-61.28062900	-11.12221000	-21.16608800
H130	-60.93332500	-7.67610500	-17.73504200
H131	-59.41346800	-8.61675000	-17.62134800
H132	-64.73288100	-11.62930200	-21.77517700

dissociative II-Pr

C1	-63.97900000	-10.52500000	-15.92200000
H2	-64.84763117	-10.22467320	-16.61574401
H3	-63.21346270	-9.78284031	-16.13538477
C4	-63.49549836	-11.92039271	-16.20162329
O5	-62.78287132	-12.50095188	-15.37829944
N6	-63.86054580	-12.49628975	-17.39518039
H7	-64.34131455	-11.91092031	-18.09034351
C8	-63.11800000	-13.63200000	-17.92900000
H9	-62.73702923	-14.22539803	-17.09727049
C10	-61.88361616	-13.23469980	-18.79298462
O11	-60.92846933	-14.01014581	-18.88976105
N12	-61.96169925	-12.02157733	-19.40419557
H13	-62.87855297	-11.55870045	-19.47904750
C14	-60.88000000	-11.46800000	-20.20300000
H15	-60.14667123	-12.25419374	-20.39067387
C16	-60.10572070	-10.29008296	-19.58228507
O17	-58.88630387	-10.19646234	-19.76207986
N18	-60.82088412	-9.37732824	-18.89209129
H19	-61.83407079	-9.41277419	-18.86723289
C20	-60.17200000	-8.21800000	-18.30200000
H21	-59.74328421	-7.57173392	-19.06593386
C22	-59.06100100	-2.38000000	-17.01000100
H23	-59.77534377	-1.54923686	-16.94218646

C24	-59.62109115	-3.52480084	-17.87323695
H25	-58.89753198	-4.35246995	-17.88431111
H26	-60.52805012	-3.93378769	-17.41052281
C27	-59.90357649	-3.10523388	-19.32765044
H28	-60.60664351	-2.25614082	-19.32923615
H29	-58.97091381	-2.72028694	-19.77086182
C30	-60.44938738	-4.24588775	-20.21745660
H31	-60.27667837	-4.00583501	-21.27819097
H32	-59.86975997	-5.15658522	-20.01734195
C33	-61.92998105	-4.58106023	-20.01057028
H34	-62.19330474	-5.55496237	-20.42223314
H35	-62.18756129	-4.61731485	-18.95050980
N36	-62.84677246	-3.58383723	-20.66515383
H37	-63.29099771	-3.94786137	-21.56413179
H38	-63.67493119	-3.33782974	-19.98070279
H39	-62.36444326	-2.70800022	-20.87104117
C40	-71.94100000	-10.93600000	-25.10800000
H41	-71.63519011	-11.94233888	-25.39068327
C42	-70.96856767	-10.37318271	-24.05041923
H43	-69.96856632	-10.23216589	-24.48289365
H44	-71.28132875	-9.38218544	-23.70131244
C45	-70.79361806	-11.28009467	-22.83793507
O46	-70.27254988	-10.62581698	-21.80830571
O47	-71.08667732	-12.46872502	-22.83671776
C48	-73.16100000	-7.76700000	-16.52300000
H49	-73.55408849	-6.77996691	-16.84173021
C50	-72.33860482	-8.39622709	-17.62857662
H51	-72.95838567	-8.49718830	-18.53120995
H52	-71.53956400	-7.69479203	-17.88029317
C53	-71.71899100	-9.76626195	-17.31070635
H54	-71.11097064	-9.69857708	-16.39643497
H55	-72.51280831	-10.50436623	-17.10547972
C56	-70.84964016	-10.22174416	-18.49068668
H57	-71.45183035	-10.19474182	-19.40823168
H58	-70.04363026	-9.49219927	-18.63336978
C59	-70.21572744	-11.60961176	-18.39358589
H60	-69.62341793	-11.69471601	-17.46757818
H61	-70.99884939	-12.38111956	-18.34864572
N62	-69.39402553	-11.84169486	-19.59735305
H63	-70.01625845	-11.23960821	-21.00950166
H64	-68.51152527	-11.29046226	-19.54519267
H65	-69.11690013	-12.82048212	-19.65387191
C66	-71.18399900	-7.09500000	-20.63300000
H67	-71.13582212	-8.12449202	-20.91729554

H68	-71.10800769	-6.45949574	-21.51763120
C69	-70.11014471	-6.66870282	-19.62080739
O70	-70.32168193	-5.78827482	-18.78124514
N71	-68.94098302	-7.32998862	-19.74479020
H72	-68.15274870	-7.17438401	-19.10439033
H73	-68.83152988	-8.10741783	-20.38361044
C74	-67.38499900	-5.18800100	-22.72700000
H75	-67.52843127	-5.32048325	-21.65120747
C76	-66.11006268	-5.89752816	-23.21020974
H77	-66.21881563	-6.97390769	-23.04291213
H78	-65.97704310	-5.72109392	-24.28603994
C79	-64.84158708	-5.41792103	-22.48056117
O80	-64.28082643	-4.36246472	-22.89152675
O81	-64.42510387	-6.10696156	-21.50103997
C82	-64.66400000	-8.97600000	-26.19599900
H83	-63.59432147	-8.83710161	-26.05754616
C84	-65.30133147	-9.50889446	-24.89306927
H85	-64.83989780	-10.45835349	-24.58861602
H86	-65.12048164	-8.83104150	-24.04930346
C87	-66.80532487	-9.73655043	-25.02357271
O88	-67.41694539	-9.55517295	-26.06392469
O89	-67.44330913	-10.16409130	-23.93218479
C90	-66.15616084	-12.41205655	-21.83621963
H91	-66.37998029	-12.73973230	-22.85876476
H92	-66.97402515	-12.74192593	-21.17972354
O93	-66.03711612	-11.00126665	-21.85074643
H94	-66.85510940	-10.31398060	-23.12997113
O95	-65.35042400	-8.82300404	-20.85157934
P96	-65.69515339	-10.23242917	-20.34616821
O97	-64.54387001	-11.05301503	-19.75559007
O98	-66.99495240	-10.30199268	-19.52578070
O99	-63.54266827	-7.10978493	-18.63927105
P100	-63.66790384	-6.58170937	-17.17169751
O101	-64.88535163	-7.20084481	-16.46018204
O102	-62.35374925	-6.48328264	-16.43581224
O103	-64.10291908	-4.90482498	-17.40978248
P104	-65.26940557	-4.25136134	-18.29817561
O105	-64.71683887	-2.98683021	-18.95323420
O106	-65.91410935	-5.29240095	-19.20864654
O107	-66.38101259	-3.71361746	-17.22904564
C108	-67.21174676	-4.69513395	-16.59308298
H109	-67.68033539	-4.19404484	-15.73653724
H110	-66.61935763	-5.54681216	-16.24045541
O111	-66.53636561	-7.84701338	-18.37334740

H112	-66.68366044	-8.81186019	-18.51781764
Mg113	-65.12049520	-7.09223204	-19.82475152
H114	-67.99388277	-5.04216744	-17.27781867
H115	-65.96786555	-7.67874187	-17.53978199
H116	-58.85398666	-2.72304337	-15.98926138
H117	-58.12531439	-1.98194646	-17.42933067
H118	-72.57013123	-7.59295490	-15.61916053
H119	-74.03274445	-8.37958707	-16.23749394
H120	-72.17967833	-6.87105212	-20.16846228
H121	-71.97239675	-10.29678301	-25.97857302
H122	-72.95834133	-11.01207170	-24.68360451
H123	-67.31121350	-4.10012487	-22.91530355
H124	-68.26905889	-5.56351017	-23.25087683
H125	-65.11571846	-8.01320027	-26.46082620
H126	-64.84519140	-9.66627360	-27.01942960
H127	-63.78441084	-14.25517712	-18.53913455
H128	-64.32211992	-10.40274452	-14.91613568
H129	-61.28887277	-11.13264689	-21.16645464
H130	-60.92369058	-7.65280735	-17.71746575
H131	-59.36659837	-8.54420318	-17.63522203
H132	-65.22272082	-12.87191657	-21.49206727

dissociative III-Re

C1	-63.97900011	-10.52500002	-15.92200011
H2	-64.85146070	-10.30054686	-16.53679203
H3	-63.32130948	-9.65695802	-15.91427679
C4	-63.19327691	-11.73184485	-16.40296822
O5	-62.08335757	-12.01018058	-15.94990944
N6	-63.82069998	-12.51094544	-17.33845780
H7	-64.58758749	-12.11818078	-17.88806662
C8	-63.11800033	-13.63199993	-17.92900029
H9	-62.67969096	-14.25808252	-17.14856403
C10	-61.95225856	-13.26010180	-18.88626574
O11	-61.16718018	-14.11805162	-19.27600805
N12	-61.87960963	-11.95003470	-19.26609925
H13	-62.58824090	-11.28666230	-18.96121527
C14	-60.87999998	-11.46799992	-20.20299989
H15	-59.98489720	-12.09113513	-20.09841608
C16	-60.54126421	-9.97570703	-19.94446702
O17	-60.52763405	-9.14473065	-20.87071984
N18	-60.22574677	-9.63146111	-18.67450302
H19	-60.53033691	-10.27893663	-17.95424707
C20	-60.17200006	-8.21800000	-18.30199997
H21	-59.38759551	-7.71892464	-18.87770076
C22	-59.06100003	-2.38000008	-17.01000004

H23	-59.51788423	-1.48559799	-17.45059878
C24	-59.44791633	-3.64932449	-17.79618385
H25	-58.99778422	-4.52655809	-17.30988768
H26	-60.53571375	-3.78979744	-17.73530034
C27	-59.00716314	-3.60970872	-19.27233292
H28	-59.50044329	-2.76598162	-19.77659976
H29	-57.93047685	-3.39684180	-19.31210864
C30	-59.25519768	-4.89963374	-20.09128076
H31	-58.78721617	-4.77266953	-21.07862211
H32	-58.74566174	-5.74537873	-19.61249594
C33	-60.73991821	-5.21415325	-20.28085760
H34	-61.23283738	-5.48983678	-19.34891494
H35	-61.27575133	-4.34952620	-20.68132640
N36	-61.02320706	-6.34179949	-21.24420142
H37	-60.81772340	-7.31050830	-20.89250549
H38	-62.05916750	-6.27260960	-21.48083572
H39	-60.49405320	-6.22323561	-22.11206500
C40	-71.94102674	-10.93608162	-25.10818179
H41	-72.54928625	-11.66055703	-24.55825229
C42	-70.46283359	-11.08358021	-24.75547786
H43	-70.07854869	-12.07333446	-25.03580993
H44	-69.84820368	-10.35808896	-25.30631157
C45	-70.17469058	-10.87368894	-23.27299462
O46	-68.93383201	-11.01092891	-22.89731036
O47	-71.09928367	-10.58730968	-22.49673145
C48	-73.16099980	-7.76700014	-16.52300005
H49	-73.24150453	-6.85824868	-15.91577326
C50	-71.74871247	-8.36308278	-16.44597720
H51	-71.01926642	-7.62002060	-16.79772632
H52	-71.49119429	-8.56930961	-15.39770681
C53	-71.58968683	-9.64750557	-17.27441940
H54	-72.24547377	-10.43201835	-16.86954569
H55	-71.93696664	-9.45722491	-18.29995081
C56	-70.13750329	-10.14997499	-17.33731008
H57	-69.49896866	-9.33872880	-17.70768583
H58	-69.77596762	-10.39948955	-16.32984153
C59	-69.99381131	-11.35831911	-18.26466088
H60	-70.42374368	-12.26711423	-17.83223904
H61	-70.49679555	-11.17307777	-19.21720709
N62	-68.56695035	-11.64741745	-18.61616574
H63	-68.52111217	-12.29436199	-19.45913238
H64	-68.05015775	-10.78393587	-18.92749197
H65	-68.01891988	-12.05151677	-17.85392638
C66	-71.18413485	-7.09504133	-20.63294193

H67	-71.86112750	-7.75986770	-21.17485196
H68	-71.03537251	-6.17712428	-21.21102785
C69	-69.84696534	-7.73599421	-20.36953129
O70	-68.92301030	-7.07308141	-19.82107466
N71	-69.71359338	-9.00929250	-20.70694889
H72	-68.77254561	-9.40510239	-20.62549888
H73	-70.39358386	-9.52520191	-21.28464286
C74	-67.38511359	-5.18787328	-22.72674847
H75	-67.88510211	-5.41331842	-21.78240241
C76	-67.82053051	-6.15628640	-23.82988668
H77	-68.89971010	-6.06800195	-24.02562520
H78	-67.33645574	-5.92157004	-24.78574257
C79	-67.55979439	-7.62733227	-23.54913464
O80	-67.28774788	-8.04989082	-22.38560915
O81	-67.64490188	-8.46563315	-24.51453249
C82	-64.66386440	-8.97570415	-26.19573778
H83	-64.58231165	-8.03417046	-26.74683898
C84	-65.90042002	-9.75217694	-26.68552100
H85	-66.79159786	-9.15479278	-26.47231223
H86	-65.84207772	-9.92849185	-27.76698068
C87	-66.05626849	-11.06057690	-25.94006827
O88	-66.35456993	-11.14730441	-24.75858150
O89	-65.82590538	-12.20079286	-26.60704123
C90	-67.64127290	-13.91396721	-21.34882975
H91	-66.67219976	-13.40832104	-21.38576093
H92	-67.86730991	-14.35572822	-22.32714035
O93	-68.68794250	-13.01525249	-20.95524650
H94	-68.75229636	-12.29988775	-21.63359516
O95	-67.17707872	-9.60320968	-19.85414925
P96	-65.93714450	-10.47473408	-20.33001877
O97	-66.05920614	-10.83496822	-21.81533312
O98	-65.68508310	-11.60096278	-19.35847095
O99	-64.67481917	-9.37109380	-20.31786189
P100	-64.11725722	-8.63699390	-18.97426458
O101	-65.34193283	-8.21468003	-18.15955229
O102	-62.98982255	-9.38643829	-18.35665108
O103	-63.49063543	-7.24948835	-19.61601511
P104	-64.30825131	-5.96430239	-20.24952102
O105	-63.65035989	-5.67465169	-21.57107432
O106	-65.80504428	-6.19365197	-20.11370169
O107	-63.86346843	-4.76420632	-19.23811481
C108	-64.43003965	-4.69842656	-17.92861836
H109	-63.89276864	-3.91269232	-17.39104803
H110	-64.31177764	-5.64953885	-17.39725920

O111	-67.54721411	-7.21074653	-17.37851549
H112	-66.64021531	-7.42419958	-17.07005994
Mg113	-66.98425979	-7.65299703	-19.42177394
H114	-65.49225807	-4.43631159	-17.98348452
H115	-67.71546867	-6.27418765	-17.19454145
H116	-59.39379157	-2.44887919	-15.96866334
H117	-57.97413349	-2.23334661	-17.00676647
H118	-73.91481185	-8.47713057	-16.16122268
H119	-73.42430850	-7.50350088	-17.55503156
H120	-71.64486857	-6.80506685	-19.68191430
H121	-72.10342978	-11.09337300	-26.18124181
H122	-72.30748106	-9.93869620	-24.84743926
H123	-66.30958511	-5.24752259	-22.54827775
H124	-67.63344031	-4.15942888	-23.01702706
H125	-63.73820220	-9.54206204	-26.34605013
H126	-64.75814960	-8.73960194	-25.13305754
H127	-63.83127321	-14.23389536	-18.49990093
H128	-64.30543068	-10.71880847	-14.89098804
H129	-61.21718204	-11.52819667	-21.24427306
H130	-61.14092174	-7.73887262	-18.46690049
H131	-59.91734276	-8.15170417	-17.24149289
H132	-67.60837561	-14.71471125	-20.60451282
Mg133	-67.21634977	-10.04905558	-23.18476534
H134	-65.58916480	-12.00884517	-27.52930631

dissociative III-TS

C1	-63.97900000	-10.52500000	-15.92200000
H2	-64.88922800	-10.33708500	-16.49533300
H3	-63.35170700	-9.63711500	-15.99069000
C4	-63.18513600	-11.72082100	-16.41282900
O5	-62.06088800	-11.97099600	-15.98211800
N6	-63.81583200	-12.51527300	-17.32886800
H7	-64.63551200	-12.17850200	-17.83127600
C8	-63.11800000	-13.63200000	-17.92900000
H9	-62.68150200	-14.26884500	-17.15578000
C10	-61.95221700	-13.26285600	-18.88626900
O11	-61.17416000	-14.12725700	-19.27579400
N12	-61.87722400	-11.95507900	-19.26553000
H13	-62.57148600	-11.27954200	-18.94978800
C14	-60.88000000	-11.46800000	-20.20300000
H15	-59.97589800	-12.07696600	-20.09314700
C16	-60.56693400	-9.96983300	-19.94684100
O17	-60.59488600	-9.13306700	-20.86574200
N18	-60.22356500	-9.62881500	-18.68106400
H19	-60.49567500	-10.28613800	-17.95691800

C20	-60.17200000	-8.21800000	-18.30200000
H21	-59.42956200	-7.70360700	-18.91707900
C22	-59.06100000	-2.38000000	-17.01000000
H23	-59.52142200	-1.48579100	-17.44712300
C24	-59.44406400	-3.64503100	-17.79955100
H25	-58.99902900	-4.52546800	-17.31436700
H26	-60.53242200	-3.78076700	-17.74271100
C27	-58.99761600	-3.60302700	-19.27299100
H28	-59.46314100	-2.73826600	-19.76774200
H29	-57.91448600	-3.42653400	-19.31084700
C30	-59.29748500	-4.87437700	-20.10135700
H31	-58.86031600	-4.74772400	-21.10253700
H32	-58.79678000	-5.73921300	-19.64837000
C33	-60.79617400	-5.13468900	-20.22983600
H34	-61.26427800	-5.38842400	-19.27956500
H35	-61.31694400	-4.25122600	-20.60892600
N36	-61.17818400	-6.24696200	-21.17812500
H37	-60.96157200	-7.21984300	-20.85529000
H38	-62.22903500	-6.13668000	-21.31450000
H39	-60.72335800	-6.13005000	-22.08736700
C40	-71.94101400	-10.93606200	-25.10817700
H41	-72.60354300	-10.16622200	-24.70164000
C42	-71.59786200	-11.96020600	-24.02013200
H43	-72.51627600	-12.48289300	-23.71602300
H44	-70.90248800	-12.71890900	-24.39269800
C45	-70.99493200	-11.32571600	-22.75419000
O46	-69.95406200	-11.87700700	-22.28831700
O47	-71.59162500	-10.31679900	-22.27876400
C48	-73.16100000	-7.76700000	-16.52300000
H49	-73.39946500	-6.74681600	-16.20145400
C50	-71.69384000	-8.10418300	-16.26053900
H51	-71.05281200	-7.38778500	-16.79263900
H52	-71.46947900	-7.98020300	-15.19159400
C53	-71.31445200	-9.52517300	-16.69872500
H54	-71.88882400	-10.26185700	-16.11879000
H55	-71.59023700	-9.66808200	-17.75241700
C56	-69.81025600	-9.77107800	-16.54633600
H57	-69.27933500	-9.00301600	-17.12196000
H58	-69.51209800	-9.63645800	-15.49591400
C59	-69.36016800	-11.14578100	-17.03579400
H60	-69.64828400	-11.95494200	-16.35838300
H61	-69.73145700	-11.37218400	-18.03787900
N62	-67.86642100	-11.14575600	-17.16999600
H63	-67.38253800	-10.95870500	-16.28861800

H64	-67.46077900	-11.99078200	-17.60934100
H65	-67.60626300	-10.41273600	-17.88519500
C66	-71.18413500	-7.09504200	-20.63294200
H67	-71.85862500	-7.68646900	-21.25690500
H68	-70.90664000	-6.17270300	-21.15276600
C69	-69.94354200	-7.86026000	-20.27040600
O70	-68.98753800	-7.24342300	-19.70754200
N71	-69.93267100	-9.15183000	-20.53632700
H72	-69.07729800	-9.65760500	-20.30890000
H73	-70.65269500	-9.62397400	-21.15689700
C74	-67.38511400	-5.18787300	-22.72674800
H75	-67.33845800	-5.12981000	-21.63693700
C76	-67.80880500	-6.59552100	-23.18930400
H77	-68.79360200	-6.83751600	-22.77288300
H78	-67.88665600	-6.64129800	-24.27884100
C79	-66.85864700	-7.68741400	-22.75303100
O80	-66.68516400	-7.97146700	-21.51668500
O81	-66.21070200	-8.36740500	-23.60736700
C82	-64.66386400	-8.97570400	-26.19573800
H83	-65.41190300	-8.90564700	-26.99083000
C84	-64.75937600	-10.36896400	-25.53443900
H85	-65.74099800	-10.48573100	-25.07104200
H86	-64.64249700	-11.16192200	-26.28413400
C87	-63.70118900	-10.52094600	-24.46659500
O88	-63.85628100	-10.23802400	-23.28283600
O89	-62.48901100	-10.93418200	-24.84117700
C90	-68.37720700	-13.61856900	-20.39241700
H91	-67.95970400	-13.78715000	-21.39031500
H92	-69.33461700	-14.14682800	-20.31277700
O93	-68.56999100	-12.22016000	-20.15367500
H94	-69.14653000	-11.90937700	-20.94022000
O95	-67.42302400	-9.87362500	-19.48906200
P96	-66.58527700	-11.04245200	-20.07800500
O97	-66.51084900	-11.16606400	-21.59848700
O98	-66.09039100	-12.06263300	-19.08488700
O99	-64.85544000	-9.90962400	-20.23178500
P100	-64.27270200	-8.92960600	-19.11642700
O101	-65.43867300	-8.44114000	-18.24427400
O102	-63.04134300	-9.45770000	-18.45433100
O103	-63.85405200	-7.61863400	-20.05483100
P104	-64.38970400	-6.06440900	-19.94620700
O105	-63.78389500	-5.42320300	-21.16980900
O106	-65.87858500	-6.02913200	-19.63679900
O107	-63.56637500	-5.46552600	-18.66260000

C108	-64.08501000	-5.51025400	-17.33167300
H109	-63.41369400	-4.90621400	-16.71597800
H110	-64.10655900	-6.53605600	-16.95073600
O111	-67.45415300	-7.11740200	-17.35775000
H112	-66.50956300	-7.31185200	-17.18421000
Mg113	-67.05273700	-7.75651200	-19.44160000
H114	-65.09061600	-5.07960600	-17.29468600
H115	-67.52443600	-6.15371600	-17.45422100
H116	-59.39279600	-2.45281400	-15.96878600
H117	-57.97488700	-2.22815500	-17.00649600
H118	-73.83107100	-8.44942100	-15.98537200
H119	-73.40044300	-7.84529200	-17.59064100
H120	-71.70776800	-6.80029700	-19.71572500
H121	-72.43628800	-11.41109400	-25.96384100
H122	-71.03719200	-10.43666700	-25.47908300
H123	-66.39551200	-4.92488400	-23.11454100
H124	-68.10676400	-4.44975500	-23.09204800
H125	-63.67711100	-8.80878600	-26.64361200
H126	-64.87211800	-8.19667000	-25.46269200
H127	-63.83859300	-14.22335500	-18.50099000
H128	-64.23447000	-10.68820300	-14.86643300
H129	-61.21617100	-11.53700800	-21.24384400
H130	-61.15711100	-7.75212800	-18.41440300
H131	-59.86218600	-8.15150500	-17.25641200
H132	-67.67867500	-13.99449300	-19.64208900
Mg133	-65.41254100	-9.59849200	-22.13197300
H134	-62.48005500	-11.15191900	-25.78868000

dissociative III-Pr

C1	-63.97900003	-10.52000004	-15.92200006
H2	-64.77997629	-10.24530880	-16.61005776
H3	-63.30264333	-9.67086168	-15.82820478
C4	-63.18341967	-11.71750053	-16.41317081
O5	-62.05816975	-11.97267970	-15.98580696
N6	-63.81795377	-12.50664198	-17.33003823
H7	-64.67916093	-12.19432796	-17.78290269
C8	-63.12300014	-13.62699991	-17.92900005
H9	-62.65133994	-14.24000445	-17.15652081
C10	-61.99363415	-13.25401512	-18.92662182
O11	-61.25544105	-14.12825311	-19.37193729
N12	-61.89091101	-11.93912449	-19.26681013
H13	-62.54638413	-11.24457038	-18.90839300
C14	-60.88500001	-11.46300001	-20.20300004
H15	-59.98586435	-12.07886930	-20.09037271
C16	-60.56293129	-9.96261194	-19.94639936

O17	-60.60818185	-9.12094594	-20.85669657
N18	-60.18649717	-9.62627741	-18.68451066
H19	-60.46943722	-10.27744696	-17.95878358
C20	-60.17700003	-8.21300001	-18.30199999
H21	-59.51804001	-7.66502036	-18.97957026
C22	-59.06100001	-2.38000003	-17.01000002
H23	-59.65621764	-1.50519124	-17.29868710
C24	-59.53635406	-3.64766704	-17.75096947
H25	-58.93784154	-4.50676174	-17.41579197
H26	-60.57304422	-3.86498512	-17.46053752
C27	-59.43558130	-3.53160136	-19.28457125
H28	-60.08297382	-2.71272634	-19.63070833
H29	-58.41032825	-3.23665172	-19.54640976
C30	-59.76805018	-4.81076710	-20.09142919
H31	-59.51417726	-4.62177259	-21.14505429
H32	-59.12301781	-5.63462980	-19.76068911
C33	-61.23975619	-5.21987467	-20.00352321
H34	-61.52369106	-5.57155147	-19.01142794
H35	-61.89883601	-4.38383798	-20.25010747
N36	-61.63784878	-6.32222618	-20.95676039
H37	-61.29909417	-7.28216637	-20.71629738
H38	-62.69691941	-6.30915973	-20.99631181
H39	-61.30085159	-6.12465672	-21.90263874
C40	-71.92601388	-10.94606207	-25.09317689
H41	-72.77178531	-10.94082322	-24.40018501
C42	-70.85155313	-11.92657144	-24.62538241
H43	-71.23912764	-12.95546560	-24.63539267
H44	-69.97909768	-11.92777081	-25.28705731
C45	-70.38566245	-11.64677752	-23.20825108
O46	-69.15777026	-12.11207914	-22.98518687
O47	-71.07428750	-11.07816241	-22.37426451
C48	-73.16099993	-7.77200004	-16.52300002
H49	-73.54195905	-6.85899459	-16.05147986
C50	-71.87162686	-8.25022724	-15.84794570
H51	-71.11815032	-7.45275523	-15.91263869
H52	-72.05126965	-8.41580756	-14.77628387
C53	-71.29646475	-9.52604282	-16.48261544
H54	-71.97723712	-10.37158445	-16.30840895
H55	-71.24476231	-9.38974557	-17.57136776
C56	-69.88583054	-9.85257036	-15.96657858
H57	-69.28177650	-8.94001287	-16.03638708
H58	-69.91812488	-10.13445524	-14.90437491
C59	-69.19098474	-10.93752039	-16.79456167
H60	-69.53353821	-11.94809038	-16.55332577

H61	-69.33945729	-10.76343390	-17.86018149
N62	-67.70040897	-10.89901541	-16.61035697
H63	-67.40961653	-11.27393815	-15.70498381
H64	-67.17165790	-11.42385966	-17.41272132
H65	-67.39630201	-9.91070555	-16.66310513
C66	-71.17913499	-7.07004202	-20.62794197
H67	-71.98730294	-7.61396075	-21.12467921
H68	-70.74577677	-6.34814997	-21.32856370
C69	-70.09035340	-7.96823337	-20.12179902
O70	-69.12533768	-7.45858356	-19.49696087
N71	-70.20559098	-9.27293036	-20.34893303
H72	-69.38107211	-9.84859449	-20.11436902
H73	-70.87908973	-9.66473333	-20.99876120
C74	-67.38011400	-5.19287286	-22.72674804
H75	-67.57238136	-5.16251861	-21.65049053
C76	-67.61820539	-6.61060521	-23.28852681
H77	-68.65660451	-6.90829337	-23.09284347
H78	-67.46813281	-6.61737268	-24.37239359
C79	-66.72309192	-7.69139831	-22.70020551
O80	-66.89603958	-8.11434320	-21.50930623
O81	-65.82249627	-8.23040452	-23.40874501
C82	-64.66386397	-8.97070402	-26.19573792
H83	-65.43319489	-8.72573066	-26.93443482
C84	-65.02336731	-10.30238523	-25.50351195
H85	-65.96180987	-10.18614822	-24.96111192
H86	-65.14930168	-11.10272829	-26.24530337
C87	-63.95271454	-10.71270565	-24.51311080
O88	-64.00534911	-10.54438706	-23.30559655
O89	-62.84294779	-11.27341331	-25.01656352
C90	-67.72457637	-13.73601891	-20.63542931
H91	-66.89296540	-13.84079908	-21.34129516
H92	-68.59506168	-14.27762966	-21.01420613
O93	-68.11050607	-12.35846202	-20.50277213
H94	-68.87697571	-12.00160411	-22.03248357
O95	-67.80465161	-10.07379776	-19.55274917
P96	-66.94131611	-11.31136498	-19.89340075
O97	-65.95546863	-11.10766490	-21.05396096
O98	-66.39080285	-12.00566306	-18.63515658
O99	-63.84420680	-8.63845086	-20.74449613
P100	-63.98031633	-8.60804593	-19.20230966
O101	-65.44436810	-8.93416599	-18.79097271
O102	-62.93490481	-9.34017787	-18.41546759
O103	-63.82540786	-6.97320656	-18.80302121
P104	-64.79358910	-5.83055411	-19.48792103

O105	-64.24900445	-5.48752677	-20.85124748
O106	-66.25298840	-6.23902414	-19.29092034
O107	-64.46777329	-4.56738943	-18.51030040
C108	-64.95713406	-4.57617570	-17.17190014
H109	-64.61875090	-3.64823107	-16.70340643
H110	-64.55341183	-5.42848738	-16.61080179
O111	-67.09336973	-8.12480907	-17.11126891
H112	-66.18286149	-8.34645743	-17.48477247
Mg113	-67.17362221	-8.08189507	-19.47525407
H114	-66.05266693	-4.60780067	-17.15880093
H115	-67.06683499	-7.19173252	-16.84795216
H116	-59.15134398	-2.50461523	-15.92542047
H117	-58.01079982	-2.15989699	-17.23742797
H118	-73.95081662	-8.53117776	-16.46379839
H119	-72.99131795	-7.55354497	-17.58458174
H120	-71.59282307	-6.49378150	-19.79357891
H121	-72.29173054	-11.21946744	-26.08844862
H122	-71.53227149	-9.92496024	-25.14555904
H123	-66.34373939	-4.87784481	-22.87984774
H124	-68.04562247	-4.48238839	-23.22921922
H125	-63.70170830	-9.02815901	-26.71923928
H126	-64.62876613	-8.16938121	-25.45669739
H127	-63.84885506	-14.24439439	-18.46600532
H128	-64.39146734	-10.74732187	-14.92838324
H129	-61.22065254	-11.53083186	-21.24363242
H130	-61.19150269	-7.80661195	-18.31785036
H131	-59.77493870	-8.13252752	-17.28808163
H132	-67.42852171	-14.13498547	-19.66177376
Mg133	-65.14538603	-9.52152934	-21.87635438
H134	-62.91891512	-11.36793120	-25.98081013

dissociative IV-Re

C1	-63.97900001	-10.52500005	-15.92200001
H2	-64.90025227	-10.33304161	-16.47448164
H3	-63.35168056	-9.63602401	-15.97564021
C4	-63.19049254	-11.70998889	-16.44194433
O5	-62.04716382	-11.95016402	-16.04273442
N6	-63.82857048	-12.51767227	-17.33882137
H7	-64.66195064	-12.20246582	-17.84183934
C8	-63.11800007	-13.63199984	-17.92900006
H9	-62.70976977	-14.28848535	-17.15632402
C10	-61.92010523	-13.25509453	-18.84573772
O11	-61.08638008	-14.10091453	-19.16064884
N12	-61.88998658	-11.96588719	-19.28734276
H13	-62.62487564	-11.31136517	-19.01608978

C14	-60.88000008	-11.46799998	-20.20299990
H15	-59.98425759	-12.08970435	-20.09171061
C16	-60.54101886	-9.97993401	-19.93337143
O17	-60.45896714	-9.15541387	-20.86003239
N18	-60.28197892	-9.63284573	-18.64885245
H19	-60.65237434	-10.25595056	-17.93644193
C20	-60.17200002	-8.21800001	-18.30200004
H21	-59.40736800	-7.75285857	-18.92817945
C22	-59.06100001	-2.38000005	-17.01000003
H23	-59.51930242	-1.48539816	-17.44915634
C24	-59.46666954	-3.65031783	-17.78296757
H25	-59.01462423	-4.52777859	-17.29885997
H26	-60.55356246	-3.78482668	-17.70576951
C27	-59.04590604	-3.61960011	-19.26443351
H28	-59.54150666	-2.77490296	-19.76531193
H29	-57.96806024	-3.41278284	-19.31915314
C30	-59.31549653	-4.91128169	-20.07088336
H31	-58.85132398	-4.79867475	-21.06196695
H32	-58.81223582	-5.75883805	-19.58869090
C33	-60.80441663	-5.21294522	-20.25217527
H34	-61.30071851	-5.47863533	-19.31754895
H35	-61.33450549	-4.34311299	-20.65026507
N36	-61.10579714	-6.33060223	-21.21366049
H37	-60.89067847	-7.29802495	-20.87648024
H38	-62.16863418	-6.23772297	-21.41702490
H39	-60.60196650	-6.20667220	-22.09500290
C40	-71.94099998	-10.93600004	-25.10800002
H41	-72.52267365	-10.02116057	-24.96764210
C42	-72.12801037	-11.86637097	-23.90239498
H43	-73.17853789	-12.18945865	-23.84133447
H44	-71.52916305	-12.77853479	-24.00508182
C45	-71.77495087	-11.18925421	-22.56491390
O46	-72.03374166	-9.95905103	-22.45862841
O47	-71.25073199	-11.89468932	-21.64841797
C48	-73.16099997	-7.76700003	-16.52299999
H49	-73.11563576	-6.84151185	-15.93675479
C50	-71.78200063	-8.43575960	-16.61666657
H51	-71.06828197	-7.73645431	-17.07396364
H52	-71.40185620	-8.63877652	-15.60507848
C53	-71.79521097	-9.73866929	-17.43081671
H54	-72.46629803	-10.46576214	-16.94872023
H55	-72.22247063	-9.54021498	-18.42360768
C56	-70.39863727	-10.35597480	-17.61368890
H57	-69.73396539	-9.61179986	-18.06853693

H58	-69.96467961	-10.60852910	-16.63481592
C59	-70.44583170	-11.59299370	-18.51210666
H60	-70.94742289	-12.43394456	-18.02053882
H61	-70.96748574	-11.39399767	-19.45456393
N62	-69.08017290	-12.05798632	-18.92012619
H63	-68.53567520	-12.46788986	-18.15783608
H64	-69.17616089	-12.78358985	-19.69368098
H65	-68.48471776	-11.26013678	-19.28299985
C66	-71.18399983	-7.09500006	-20.63299999
H67	-71.95262831	-7.67585713	-21.14733422
H68	-70.95629033	-6.19247262	-21.21070021
C69	-69.90988355	-7.89764430	-20.45644913
O70	-68.91119904	-7.39315588	-19.85291552
N71	-69.92605907	-9.12135230	-20.94837993
H72	-69.07469352	-9.68092892	-20.88947173
H73	-70.75368146	-9.47157291	-21.48411841
C74	-67.38500000	-5.18799992	-22.72699996
H75	-67.84417804	-5.86694484	-22.00743565
C76	-67.93520905	-5.42260982	-24.14416163
H77	-69.02274057	-5.31594892	-24.19804406
H78	-67.49021862	-4.70578426	-24.84779291
C79	-67.52581547	-6.81299391	-24.56549081
O80	-66.37904565	-7.21259511	-24.37775488
O81	-68.48590945	-7.56960621	-25.07717966
C82	-64.66400003	-8.97600001	-26.19599999
H83	-64.21438673	-8.74187227	-27.16943422
C84	-65.77390864	-10.02956885	-26.37330876
H85	-66.57249897	-9.64898064	-27.01767720
H86	-65.35898905	-10.93348960	-26.83488042
C87	-66.38069039	-10.40748715	-25.03635725
O88	-65.57713968	-11.14912096	-24.30460253
O89	-67.49463578	-10.02657721	-24.66735296
C90	-68.58748285	-14.07849767	-21.87838457
H91	-68.97670226	-14.62330317	-22.75096575
H92	-67.83767709	-14.70395582	-21.38468678
O93	-69.63134733	-13.82944739	-20.93433280
H94	-70.30182422	-13.22364853	-21.36458803
O95	-67.39963296	-10.11056210	-19.87696076
P96	-66.19957377	-11.02365876	-20.37163293
O97	-66.39619394	-11.30655613	-21.86264946
O98	-65.91892716	-12.14661282	-19.40272771
O99	-64.86367391	-9.95864181	-20.39742117
P100	-64.34338280	-8.98873726	-19.20884003
O101	-65.58268096	-8.44624127	-18.49606775

O102	-63.18634339	-9.57511661	-18.46647668
O103	-63.72237888	-7.72115058	-20.08934895
P104	-64.42322654	-6.25321581	-20.30267237
O105	-63.63547296	-5.64506893	-21.44400732
O106	-65.93109373	-6.39504000	-20.36437372
O107	-64.00597700	-5.41258783	-18.96742228
C108	-64.88780253	-5.35806534	-17.83426888
H109	-64.37434891	-4.75705170	-17.07822395
H110	-65.09267138	-6.35946320	-17.44860491
Mg111	-67.00355194	-8.10317566	-19.99773410
H112	-65.83056991	-4.87562509	-18.11030803
H113	-59.37976510	-2.43989139	-15.96352558
H114	-57.97294909	-2.23899217	-17.02304136
H115	-73.89386947	-8.42977140	-16.04527259
H116	-73.54507549	-7.51377289	-17.51892758
H117	-71.54803772	-6.76864554	-19.65232948
H118	-72.25732312	-11.42201487	-26.04033285
H119	-70.88945252	-10.64527369	-25.21561777
H120	-66.30715380	-5.33972054	-22.69853091
H121	-67.59999741	-4.16326950	-22.39959522
H122	-63.87575940	-9.36671320	-25.54436535
H123	-65.05580906	-8.06298317	-25.75324069
H124	-63.82784648	-14.19910390	-18.53810242
H125	-64.22464457	-10.71243231	-14.86748332
H126	-61.20355727	-11.51881202	-21.24922371
H127	-61.13587790	-7.71139230	-18.43177023
H128	-59.86789408	-8.13789467	-17.25507225
H129	-68.09815953	-13.15551306	-22.21034825
O130	-66.99984709	-8.52362607	-22.03170383
H131	-66.93694420	-9.48564104	-22.23929652
H132	-66.51708828	-8.04543683	-22.73276356
H133	-65.91382908	-11.26214844	-23.34598605
H134	-68.14770500	-8.51603605	-25.10035673

dissociative IV-TS

C1	-63.97900000	-10.52500000	-15.92200000
H2	-64.95076800	-10.38293700	-16.39661600
H3	-63.33113700	-9.71761100	-16.27371300
C4	-63.31513700	-11.85820300	-16.25299100
O5	-62.37035300	-12.29255400	-15.59548800
N6	-63.84785200	-12.53267700	-17.32336500
H7	-64.39456200	-11.95567500	-17.95807600
C8	-63.11800000	-13.63200000	-17.92900000
H9	-62.74666200	-14.29593300	-17.14670500
C10	-61.88347200	-13.25288700	-18.79979000

O11	-61.03331800	-14.09866500	-19.06644600
N12	-61.84947000	-11.96313600	-19.24214300
H13	-62.67932300	-11.36794100	-19.12384900
C14	-60.88000000	-11.46800000	-20.20300000
H15	-59.98018100	-12.08967900	-20.13435300
C16	-60.51570700	-9.98944800	-19.95791900
O17	-60.28377300	-9.21615400	-20.89139700
N18	-60.40590100	-9.61146500	-18.65557900
H19	-60.82255500	-10.22882500	-17.96866000
C20	-60.17200000	-8.21800000	-18.30200000
H21	-59.40409900	-7.81201400	-18.96375500
C22	-59.06100000	-2.38000000	-17.01000000
H23	-59.57652700	-1.47165600	-17.34616900
C24	-59.53077000	-3.60972400	-17.80362200
H25	-59.01772900	-4.50510100	-17.42426800
H26	-60.60070300	-3.76981600	-17.61787200
C27	-59.27446000	-3.48574300	-19.31519700
H28	-59.82228500	-2.61313300	-19.70226200
H29	-58.20853900	-3.26627600	-19.47239800
C30	-59.64404000	-4.72521400	-20.15483300
H31	-59.29438800	-4.56424900	-21.18535100
H32	-59.09854800	-5.60313300	-19.78221500
C33	-61.14842900	-5.03291700	-20.19786000
H34	-61.52079200	-5.31041700	-19.20652200
H35	-61.69844300	-4.12823600	-20.48783100
N36	-61.56219600	-6.10485900	-21.12618800
H37	-61.20259600	-7.02717500	-20.85860500
H38	-63.15066800	-6.02871800	-21.13772900
H39	-61.20827700	-5.92281600	-22.06523400
C40	-71.94100000	-10.93600000	-25.10800000
H41	-73.02833000	-11.04376200	-25.03374400
C42	-71.22895200	-11.77992400	-24.04577300
H43	-71.42142000	-12.85221000	-24.19154600
H44	-70.14051900	-11.65331800	-24.13289100
C45	-71.60745100	-11.41180300	-22.60433000
O46	-72.37996100	-10.44585100	-22.41420500
O47	-71.07459600	-12.09386800	-21.65193100
C48	-73.16100000	-7.76700000	-16.52300000
H49	-73.04652500	-6.79651600	-16.02641400
C50	-71.83704400	-8.54141000	-16.53929900
H51	-71.07342700	-7.94571800	-17.05684500
H52	-71.47420100	-8.67341000	-15.50981100
C53	-71.93971600	-9.91260900	-17.22534800
H54	-72.64063000	-10.55531200	-16.67176900

H55	-72.36071800	-9.78171900	-18.23053100
C56	-70.57197800	-10.60108200	-17.35399400
H57	-69.87826000	-9.90849500	-17.84595300
H58	-70.15826900	-10.80768100	-16.35576400
C59	-70.63216600	-11.89294100	-18.17022200
H60	-71.17373200	-12.69009500	-17.65021900
H61	-71.09866200	-11.74043300	-19.14881800
N62	-69.24512200	-12.39195500	-18.46537200
H63	-68.75361200	-12.74626500	-17.64254100
H64	-69.23534200	-13.09865000	-19.23050200
H65	-68.66311400	-11.61675400	-18.91612300
C66	-71.18400000	-7.09500000	-20.63300000
H67	-72.14362700	-7.46629600	-21.00122300
H68	-70.70465000	-6.50928200	-21.42535800
C69	-70.24404200	-8.20493900	-20.24983800
O70	-69.16965600	-7.93880800	-19.64782300
N71	-70.60392300	-9.43758600	-20.57471800
H72	-69.88878300	-10.16042300	-20.52362000
H73	-71.42706600	-9.65885100	-21.16152300
C74	-67.38500000	-5.18800000	-22.72700000
H75	-67.85821000	-5.84604000	-21.99812800
C76	-67.98205100	-5.37831100	-24.12927600
H77	-69.06184800	-5.19906800	-24.15552700
H78	-67.52415800	-4.67590000	-24.84109500
C79	-67.69363300	-6.77870300	-24.62441700
O80	-66.64781000	-7.35742300	-24.35226600
O81	-68.66284900	-7.33315200	-25.34911300
C82	-64.66400000	-8.97600000	-26.19600000
H83	-64.10769000	-8.58382400	-27.05614500
C84	-65.94178000	-9.68426000	-26.67546300
H85	-66.58974000	-9.00070200	-27.23165200
H86	-65.67809500	-10.51517400	-27.34376100
C87	-66.74688300	-10.25522100	-25.52558100
O88	-66.05808800	-11.12533700	-24.80908200
O89	-67.91474900	-9.94673300	-25.28514100
C90	-68.56242600	-14.33744200	-21.85582400
H91	-69.37703600	-15.07174300	-21.83278100
H92	-67.65676600	-14.76507700	-21.42179200
O93	-68.91214400	-13.19082400	-21.07711400
H94	-69.77256000	-12.74534200	-21.47786900
O95	-68.05590100	-10.75177000	-20.15116200
P96	-67.19431800	-11.71179700	-21.01653500
O97	-67.27718800	-11.42489000	-22.49504200
O98	-66.30720000	-12.69329800	-20.34506000

O99	-65.52770500	-9.64937200	-20.76813900
P100	-64.94897000	-9.33723400	-19.36814100
O101	-66.15581600	-9.00083700	-18.45894300
O102	-63.83889700	-10.18454400	-18.81606800
O103	-64.15686500	-7.79762000	-19.56417700
P104	-64.99535700	-6.51320500	-19.98639400
O105	-64.17035200	-5.78696400	-21.10801200
O106	-66.42966700	-6.80907000	-20.33939200
O107	-64.89065900	-5.48684300	-18.73998800
C108	-65.71236500	-5.75709400	-17.58705600
H109	-65.30668200	-5.15026000	-16.77346900
H110	-65.68653400	-6.81811800	-17.32249700
Mg111	-67.33754700	-8.70056800	-20.17787000
H112	-66.74667100	-5.46393500	-17.79181700
H113	-59.25867400	-2.49878100	-15.93854400
H114	-57.98344600	-2.21524100	-17.13621000
H115	-73.94420300	-8.32336100	-15.99211600
H116	-73.52147000	-7.58089600	-17.54199400
H117	-71.33968100	-6.42529600	-19.78152300
H118	-71.62797900	-11.23365300	-26.11648300
H119	-71.70809800	-9.87622100	-24.97291000
H120	-66.31792600	-5.41059600	-22.72391400
H121	-67.52702000	-4.15336700	-22.39187600
H122	-64.01779500	-9.67644500	-25.65950500
H123	-64.90970300	-8.15122400	-25.52686600
H124	-63.80515200	-14.19432800	-18.57048500
H125	-64.06608800	-10.44773700	-14.83276100
H126	-61.25212100	-11.52401300	-21.23275700
H127	-61.08662500	-7.61793100	-18.39915100
H128	-59.82241900	-8.16516600	-17.26696400
H129	-68.35910300	-14.04624800	-22.89188600
O130	-67.88443400	-8.69973000	-22.21132700
H131	-67.82027700	-9.63460700	-22.51955400
H132	-67.19178700	-8.23476800	-22.72171000
H133	-66.53520900	-11.34254100	-23.95175300
H134	-68.42293800	-8.29645500	-25.47997300

dissociative IV-Pr

C1	-63.97900000	-10.52500002	-15.92199999
H2	-64.96776984	-10.37463780	-16.35825557
H3	-63.33075449	-9.76340800	-16.36188944
C4	-63.38844032	-11.90465052	-16.20624613
O5	-62.55975047	-12.42090145	-15.46021154
N6	-63.85148634	-12.51477053	-17.34915060
H7	-64.29383441	-11.87580276	-18.00858762

C8	-63.11800004	-13.63199994	-17.92900004
H9	-62.73877813	-14.26173837	-17.12325671
C10	-61.89832996	-13.25212810	-18.81687418
O11	-61.06333488	-14.10188580	-19.11363203
N12	-61.84606349	-11.95141354	-19.23072769
H13	-62.66156646	-11.34095631	-19.10560528
C14	-60.88000006	-11.46799996	-20.20299994
H15	-59.98040228	-12.08937134	-20.13244465
C16	-60.52292552	-9.98507533	-19.96509032
O17	-60.33976489	-9.20058348	-20.89869680
N18	-60.36895215	-9.61564274	-18.66312335
H19	-60.75585837	-10.24626089	-17.97137753
C20	-60.17199999	-8.21800001	-18.30200001
H21	-59.45783490	-7.77438766	-18.99850917
C22	-59.06099999	-2.38000001	-17.01000001
H23	-59.63560300	-1.49508664	-17.31098348
C24	-59.50568260	-3.62291957	-17.79681159
H25	-58.93742486	-4.49781441	-17.44916924
H26	-60.55838819	-3.83239537	-17.56719955
C27	-59.32090984	-3.47314029	-19.31681844
H28	-59.90705222	-2.60941948	-19.66607084
H29	-58.26913905	-3.22613415	-19.52112841
C30	-59.70525663	-4.71094361	-20.15286196
H31	-59.42922214	-4.52478121	-21.20118803
H32	-59.11225232	-5.57830064	-19.83090484
C33	-61.20137799	-5.05562844	-20.09580078
H34	-61.49093205	-5.36326657	-19.08585787
H35	-61.79187440	-4.15816385	-20.32173291
N36	-61.66410661	-6.11556644	-21.01262205
H37	-61.27102743	-7.03707619	-20.79544844
H38	-63.24608665	-6.02119217	-20.92274806
H39	-61.39163683	-5.90614743	-21.97282208
C40	-71.94100000	-10.93599999	-25.10799995
H41	-72.98348524	-11.26409701	-25.18339311
C42	-71.22737157	-11.61898621	-23.94002882
H43	-71.22004768	-12.71104515	-24.05891703
H44	-70.16944361	-11.31893913	-23.89379898
C45	-71.86366201	-11.28673952	-22.60247686
O46	-72.71766962	-10.42356654	-22.45731923
O47	-71.44524954	-11.97129192	-21.53162981
C48	-73.16099988	-7.76700014	-16.52300001
H49	-73.43158593	-6.70768383	-16.44649447
C50	-71.63044246	-7.94396462	-16.54931709
H51	-71.22044897	-7.38502062	-17.40112542

H52	-71.19440417	-7.48335049	-15.65117032
C53	-71.15806408	-9.40810780	-16.64764555
H54	-71.45132470	-9.95043073	-15.73548736
H55	-71.67751563	-9.89732722	-17.48310453
C56	-69.63844334	-9.53119642	-16.88304869
H57	-69.38259147	-9.01251286	-17.81195976
H58	-69.08570827	-9.01901933	-16.08374846
C59	-69.15854266	-10.98944387	-16.97569320
H60	-68.97218584	-11.41074522	-15.98239241
H61	-69.91208170	-11.61490810	-17.46617090
N62	-67.90742010	-11.11969408	-17.78914368
H63	-67.21113537	-10.32505720	-17.72573548
H64	-67.40684010	-11.99407537	-17.61332941
H65	-68.10968701	-11.09280363	-18.85789888
C66	-71.18400002	-7.09499992	-20.63300011
H67	-72.21241680	-7.42603353	-20.80096332
H68	-70.80153284	-6.65077586	-21.55881259
C69	-70.26148629	-8.21731469	-20.24613978
O70	-69.11341649	-7.95254342	-19.81930361
N71	-70.71627830	-9.46413368	-20.36136323
H72	-70.00696290	-10.20709325	-20.33901584
H73	-71.59461661	-9.66097609	-20.83466698
C74	-67.38499997	-5.18799996	-22.72700001
H75	-67.92227891	-5.88311329	-22.08057085
C76	-67.88183232	-5.26709824	-24.17801306
H77	-68.94713743	-5.03207798	-24.27292390
H78	-67.33770597	-4.55075080	-24.81067816
C79	-67.62151583	-6.65418527	-24.72238172
O80	-66.61227786	-7.28302105	-24.42272084
O81	-68.57242183	-7.13990699	-25.51455250
C82	-64.66400003	-8.97600006	-26.19599993
H83	-64.01849570	-8.66587114	-27.02727102
C84	-65.92142402	-9.67585016	-26.74297000
H85	-66.49626726	-9.00346825	-27.38740960
H86	-65.62533483	-10.54504240	-27.34560634
C87	-66.84926969	-10.17382040	-25.64689443
O88	-66.29168309	-11.07882752	-24.87531202
O89	-68.01005400	-9.77181519	-25.51142867
C90	-68.82993342	-14.31173311	-21.69463181
H91	-69.74000553	-14.89062237	-21.50268644
H92	-67.98403429	-14.74995502	-21.15814812
O93	-69.06390208	-12.98109386	-21.23199235
H94	-70.54454080	-12.40822069	-21.62377878
O95	-68.22925301	-10.76534232	-20.36194332

P96	-67.70438248	-11.93449656	-21.30561049
O97	-67.73485157	-11.41341947	-22.76041644
O98	-66.54760912	-12.71263442	-20.76513957
O99	-65.34875525	-9.34691193	-20.80406275
P100	-64.93846275	-9.23373395	-19.33564663
O101	-66.26152766	-9.15575133	-18.50085594
O102	-63.82414047	-10.08598423	-18.78845739
O103	-64.31076204	-7.62557379	-19.17756362
P104	-65.15007603	-6.41183696	-19.79249869
O105	-64.24637862	-5.71332499	-20.86866113
O106	-66.53293277	-6.79139973	-20.24078650
O107	-65.20040945	-5.30910499	-18.60695139
C108	-66.11575518	-5.55820216	-17.52942210
H109	-65.91315290	-4.79763674	-16.77071813
H110	-65.96006061	-6.55647185	-17.10737656
Mg111	-67.30381265	-8.79513584	-20.42521646
H112	-67.14819125	-5.47315622	-17.88144906
H113	-59.20379347	-2.51977937	-15.93216183
H114	-57.99946373	-2.16024303	-17.18160874
H115	-73.60980641	-8.29006686	-15.66904142
H116	-73.61964846	-8.16641061	-17.43562527
H117	-71.16175545	-6.31739714	-19.86393674
H118	-71.43180667	-11.16686739	-26.04973857
H119	-71.94268875	-9.85083744	-24.97591631
H120	-66.32578410	-5.43740061	-22.66350055
H121	-67.52848317	-4.17555937	-22.32976300
H122	-64.09639479	-9.65897135	-25.55715417
H123	-64.93305171	-8.09941324	-25.60724977
H124	-63.79768752	-14.22568108	-18.54989546
H125	-64.01332339	-10.38574463	-14.83707943
H126	-61.25842133	-11.53258228	-21.22960599
H127	-61.11448192	-7.65551488	-18.34067880
H128	-59.76751937	-8.16229183	-17.28696876
H129	-68.61404560	-14.32188882	-22.77128060
O130	-67.87265483	-8.75503289	-22.40312758
H131	-67.89042425	-9.72208750	-22.66319750
H132	-67.20003614	-8.34044880	-22.97902085
H133	-66.86319766	-11.29581863	-24.05193350
H134	-68.36558825	-8.11027064	-25.67349469

dissociative V-Re

C1	-63.97900013	-10.52500007	-15.92200018
H2	-64.95878189	-10.45709339	-16.39783793
H3	-63.42377228	-9.60135738	-16.08548553
C4	-63.16033929	-11.70248444	-16.42783835

O5	-61.99470819	-11.89626493	-16.06836902
N6	-63.80971374	-12.53135639	-17.29512843
H7	-64.66677217	-12.19283053	-17.75477821
C8	-63.11800136	-13.63199972	-17.92900142
H9	-62.71601058	-14.32924339	-17.18829480
C10	-61.90911601	-13.23817228	-18.81687903
O11	-61.01536183	-14.04600773	-19.05062005
N12	-61.92677237	-11.97060208	-19.32898068
H13	-62.75298770	-11.39914968	-19.18917763
C14	-60.88000009	-11.46799998	-20.20299982
H15	-60.00793235	-12.11462136	-20.05975506
C16	-60.49832201	-9.99940701	-19.93478604
O17	-60.15851906	-9.25489567	-20.86101092
N18	-60.50422584	-9.59431903	-18.64420973
H19	-60.90433239	-10.19908695	-17.93487264
C20	-60.17200005	-8.21800002	-18.30200005
H21	-59.20313902	-7.95681344	-18.74125736
C22	-59.06100004	-2.38000008	-17.01000003
H23	-59.38934930	-1.44401698	-17.47970914
C24	-59.46144219	-3.59566993	-17.85869101
H25	-59.16167819	-4.51885284	-17.34298835
H26	-60.55555564	-3.62948572	-17.92825185
C27	-58.84697287	-3.58312234	-19.27028243
H28	-59.12997055	-2.64649497	-19.77528863
H29	-57.75141393	-3.55896850	-19.18133499
C30	-59.24831572	-4.77482454	-20.16589738
H31	-58.70393905	-4.70411849	-21.11910980
H32	-58.93482615	-5.71836815	-19.69947161
C33	-60.75639245	-4.81303263	-20.44076546
H34	-61.30744818	-4.95066362	-19.50881326
H35	-61.08060262	-3.84422168	-20.84325375
N36	-61.25247757	-5.85061475	-21.37070893
H37	-61.05053312	-6.79690210	-21.03282212
H38	-62.82168786	-5.54907481	-21.39572478
H39	-60.80298561	-5.76982395	-22.28232591
C40	-71.94099837	-10.93599994	-25.10799849
H41	-72.92411660	-11.04917249	-24.63612413
C42	-70.83544661	-11.50767203	-24.21870799
H43	-70.96884702	-12.58190930	-24.04962790
H44	-69.85836504	-11.38697726	-24.70890140
C45	-70.75550591	-10.80550663	-22.86106041
O46	-71.07443523	-9.57371768	-22.81717626
O47	-70.38072199	-11.45829601	-21.85060152
C48	-73.16099993	-7.76700007	-16.52299994

H49	-73.23997884	-6.87223899	-15.89390022
C50	-71.76927258	-8.40039061	-16.42391633
H51	-71.00824421	-7.66927147	-16.72893469
H52	-71.54819540	-8.64857356	-15.37598330
C53	-71.62836904	-9.65880524	-17.29151180
H54	-72.31013305	-10.44119510	-16.92499247
H55	-71.95259271	-9.42496766	-18.31550446
C56	-70.18875576	-10.18610063	-17.34916941
H57	-69.52984672	-9.37302359	-17.67632763
H58	-69.84790988	-10.48133239	-16.34639991
C59	-70.04276949	-11.35620417	-18.32377889
H60	-70.45630112	-12.28747099	-17.92222948
H61	-70.54426476	-11.14689486	-19.27274234
N62	-68.61419145	-11.59661743	-18.67975829
H63	-68.03423170	-11.99624753	-17.93755525
H64	-68.53318544	-12.24006118	-19.52257080
H65	-68.11870444	-10.69949246	-18.90732039
C66	-71.18424602	-7.09542105	-20.63304202
H67	-71.74619393	-7.81289613	-21.23368295
H68	-71.17736038	-6.12100265	-21.12825886
C69	-69.76054683	-7.56021888	-20.41068487
O70	-68.87018654	-6.75487749	-20.03286551
N71	-69.53005303	-8.85749622	-20.58511857
H72	-68.56942601	-9.18682265	-20.47824194
H73	-70.16588162	-9.39515028	-21.18511542
C74	-67.38500002	-5.18800000	-22.72700015
H75	-67.74102751	-5.31075691	-21.70378968
C76	-68.45398192	-5.61723679	-23.74746349
H77	-69.34779502	-4.98802598	-23.68450959
H78	-68.06584338	-5.50580201	-24.77070928
C79	-68.87344463	-7.06961900	-23.59155142
O80	-68.09594468	-7.96554473	-23.28434775
O81	-70.16649196	-7.28131008	-23.82334751
C82	-67.37540900	-13.19530680	-21.61550294
H83	-67.02723710	-12.18059768	-21.84018852
H84	-67.46962799	-13.77045351	-22.54768331
O85	-68.61396250	-13.20381859	-20.89552122
H86	-69.28517163	-12.69148042	-21.41771878
O87	-67.02101353	-9.34143628	-19.22363151
P88	-65.76302342	-10.24157635	-19.55790173
O89	-65.44439569	-10.25369567	-21.03847420
O90	-65.87164226	-11.56208076	-18.81228232
O91	-64.40417770	-9.43866946	-18.84646579
P92	-64.19811894	-7.97835205	-18.21812969

O93	-65.55194795	-7.41363967	-17.79619607
O94	-63.02236512	-7.89218988	-17.30957826
O95	-63.83545506	-7.04884382	-19.59700299
P96	-64.42239584	-5.58009851	-19.88446269
O97	-63.81111315	-5.20288942	-21.27384391
O98	-65.91794705	-5.46251039	-19.73452263
O99	-63.66269751	-4.56525163	-18.87611468
C100	-63.98702735	-4.50741536	-17.47648388
H101	-63.50944657	-3.60155479	-17.09402232
H102	-63.59535116	-5.38733578	-16.95957206
O103	-67.96722847	-6.67328294	-17.47167442
H104	-67.08586999	-6.83522265	-17.06834905
Mg105	-66.95016536	-7.28879927	-19.41450493
H106	-65.06910795	-4.44032009	-17.33564713
H107	-68.04172794	-5.71217779	-17.58147701
H108	-59.51091160	-2.42840462	-16.01154836
H109	-57.97218782	-2.32222430	-16.88322891
H110	-73.94346155	-8.46797295	-16.20453152
H111	-73.38678260	-7.46898075	-17.55466389
H112	-71.66850214	-6.97259369	-19.65645140
H113	-71.97197205	-11.44683361	-26.07885886
H114	-71.78293696	-9.86877535	-25.28368192
H115	-66.46086321	-5.75602545	-22.83740141
H116	-67.14222328	-4.12919338	-22.87498843
H117	-63.83789973	-14.16924965	-18.55539409
H118	-64.11615049	-10.64784723	-14.83929904
H119	-61.16638725	-11.51847044	-21.25995939
H120	-60.94071831	-7.52438919	-18.65186578
H121	-60.12161158	-8.13405280	-17.21525115
H122	-66.62546827	-13.66951215	-20.97858927
O123	-66.15884752	-7.72982196	-21.35436138
H124	-65.79041631	-8.66443553	-21.38767068
H125	-66.80969253	-7.70445347	-22.08895334
H126	-70.41743848	-8.23118755	-23.58785359

dissociative V-TS

C1	-63.97900000	-10.52500000	-15.92200000
H2	-64.86776500	-10.40138500	-16.52682600
H3	-63.40176800	-9.60210700	-15.97403500
C4	-63.15051200	-11.69805100	-16.42531100
O5	-61.98796800	-11.89218900	-16.06252800
N6	-63.80212100	-12.52801500	-17.29150700
H7	-64.67411300	-12.20809000	-17.72202100
C8	-63.11800100	-13.63200000	-17.92900100
H9	-62.71047700	-14.32716600	-17.18971700

C10	-61.91513800	-13.22787000	-18.81917100
O11	-61.01838000	-14.03232900	-19.05299200
N12	-61.93296200	-11.95761700	-19.32590100
H13	-62.76006000	-11.37851800	-19.20321400
C14	-60.88000000	-11.46800000	-20.20300000
H15	-60.00244600	-12.10054100	-20.03459900
C16	-60.52969700	-9.98914700	-19.95651800
O17	-60.28429600	-9.23010600	-20.90065000
N18	-60.45176600	-9.60317500	-18.65979900
H19	-60.80544500	-10.23232900	-17.94605900
C20	-60.17200000	-8.21800000	-18.30200000
H21	-59.55057400	-7.78613200	-19.08710200
C22	-59.06100000	-2.38000000	-17.01000000
H23	-59.44347300	-1.45524400	-17.46026600
C24	-59.48096800	-3.60872500	-17.82848700
H25	-59.11414300	-4.52031600	-17.33574200
H26	-60.57568900	-3.68095800	-17.82452600
C27	-58.96437200	-3.57410000	-19.27642900
H28	-59.34782500	-2.66869100	-19.77128300
H29	-57.86982900	-3.47040900	-19.26106000
C30	-59.33209300	-4.80364400	-20.13000900
H31	-58.85880700	-4.70184500	-21.11800900
H32	-58.90946900	-5.71106700	-19.67862500
C33	-60.84490300	-4.97179400	-20.31931100
H34	-61.33403000	-5.20396000	-19.36990600
H35	-61.27594400	-4.02139900	-20.65930600
N36	-61.27969400	-6.00789600	-21.28150400
H37	-61.06527600	-6.95968600	-20.96527800
H38	-62.80278200	-5.72783800	-21.39887300
H39	-60.80707500	-5.89054600	-22.17769300
C40	-71.94099900	-10.93600000	-25.10799900
H41	-72.55213800	-11.65292300	-24.54597200
C42	-70.46569700	-11.05114000	-24.71779000
H43	-70.06961500	-12.05033800	-24.92205200
H44	-69.86917800	-10.33381600	-25.29888800
C45	-70.26814800	-10.73419200	-23.23461900
O46	-70.82631300	-9.64839400	-22.82315700
O47	-69.63106700	-11.53567800	-22.52511700
C48	-73.16100000	-7.76700000	-16.52300000
H49	-73.75254400	-6.90348800	-16.19531500
C50	-71.72548900	-7.70159100	-16.00591400
H51	-71.23774300	-6.78628700	-16.36649200
H52	-71.72219900	-7.63928000	-14.90839500
C53	-70.91755500	-8.91660900	-16.46295200

H54	-71.35216700	-9.83121900	-16.03099900
H55	-71.01961600	-9.01945100	-17.55253000
C56	-69.42686600	-8.82883100	-16.12503900
H57	-69.01782200	-7.88490100	-16.51057200
H58	-69.28112600	-8.81570200	-15.03438200
C59	-68.69075400	-10.00800700	-16.74476800
H60	-68.93052800	-10.95182900	-16.24344700
H61	-68.93673900	-10.11937700	-17.80114200
N62	-67.20730900	-9.85557400	-16.70769000
H63	-66.83890600	-9.84703600	-15.75477000
H64	-66.77978900	-10.65741400	-17.25886000
H65	-66.86843500	-8.98431800	-17.19378700
C66	-71.18424600	-7.09542100	-20.63304200
H67	-71.81655400	-7.76425800	-21.21883400
H68	-71.00583900	-6.17283800	-21.18969300
C69	-69.86327000	-7.75713600	-20.28671100
O70	-68.87532300	-7.06906100	-19.87413300
N71	-69.82833500	-9.07721400	-20.39393600
H72	-68.93066600	-9.52793500	-20.19085700
H73	-70.40561500	-9.50345800	-21.14689800
C74	-67.38500000	-5.18800000	-22.72700000
H75	-67.82676000	-5.24283300	-21.73148500
C76	-68.38229300	-5.64574600	-23.81076400
H77	-69.27130100	-5.00799800	-23.83215900
H78	-67.91037800	-5.57687800	-24.80108300
C79	-68.82240800	-7.09129000	-23.63776700
O80	-68.03480100	-8.01665000	-23.45528600
O81	-70.13341700	-7.27267900	-23.71182700
C82	-67.73509500	-13.37410100	-20.67516300
H83	-67.01552000	-13.40156000	-21.50297500
H84	-68.61549200	-13.97367600	-20.93998300
O85	-68.11639600	-12.03933900	-20.36244500
H86	-68.62871800	-11.69940100	-21.14128800
O87	-67.24224600	-9.57927200	-19.54241000
P88	-66.24578100	-10.74089400	-19.68409000
O89	-65.52856800	-10.95193700	-20.97476100
O90	-66.18645500	-11.65333800	-18.46872600
O91	-64.48963100	-9.49821400	-18.84630400
P92	-64.48036200	-8.09115500	-18.22526300
O93	-65.98131100	-7.62395800	-18.03898400
O94	-63.54997500	-7.75299900	-17.10027700
O95	-63.95137600	-7.08415100	-19.53255300
P96	-64.55876000	-5.70449700	-20.03564900
O97	-63.80749000	-5.38040100	-21.36724800

O98	-66.06690900	-5.75186900	-20.13558700
O99	-64.07403900	-4.53886900	-19.03430800
C100	-64.58073000	-4.48734500	-17.68096600
H101	-64.23537900	-3.53510200	-17.27139900
H102	-64.17442200	-5.32102300	-17.10092500
O103	-68.04407700	-5.85460800	-17.22754200
H104	-67.19799500	-6.33152200	-17.35526100
Mg105	-66.86821000	-7.62113800	-19.89962500
H106	-65.67402300	-4.51814300	-17.67428800
H107	-68.46223900	-5.91988600	-18.10334800
H108	-59.44516700	-2.43543600	-15.98508100
H109	-57.96825700	-2.29304500	-16.95388300
H110	-73.67102100	-8.67199100	-16.16855400
H111	-73.18381300	-7.78793300	-17.61972400
H112	-71.69910400	-6.83418000	-19.70025800
H113	-72.09152000	-11.13593500	-26.17664600
H114	-72.31617400	-9.93377000	-24.88473400
H115	-66.48036200	-5.79559500	-22.73120900
H116	-67.08998200	-4.14849200	-22.90723300
H117	-63.84149800	-14.17004200	-18.54978800
H118	-64.25039700	-10.71412800	-14.87361300
H119	-61.15762300	-11.54462200	-21.26029300
H120	-61.09948600	-7.64446800	-18.18367400
H121	-59.62629500	-8.19487400	-17.35328600
H122	-67.26422600	-13.79093000	-19.78168900
O123	-65.99575300	-8.23270800	-21.69682500
H124	-65.73662300	-9.18637600	-21.64201100
H125	-66.61090000	-8.14148900	-22.46221000
H126	-70.37104000	-8.24238700	-23.48483100

dissociative V-Pr

C1	-63.97900009	-10.52500000	-15.92200005
H2	-64.83818637	-10.32300726	-16.55402465
H3	-63.35164153	-9.63437616	-15.91674194
C4	-63.19753715	-11.72795681	-16.41820279
O5	-62.06885408	-11.98931037	-15.99714779
N6	-63.83648127	-12.52617307	-17.32677713
H7	-64.67928579	-12.20234367	-17.80780710
C8	-63.11800134	-13.63199984	-17.92900121
H9	-62.67793490	-14.27136503	-17.15983341
C10	-61.94521691	-13.22488978	-18.85966457
O11	-61.10118714	-14.06407929	-19.17223091
N12	-61.92203808	-11.93577442	-19.30099902
H13	-62.69064998	-11.29052135	-19.10384181
C14	-60.87999997	-11.46800000	-20.20299992

H15	-60.00939553	-12.11846646	-20.06744736
C16	-60.48508604	-9.99619049	-19.96418164
O17	-60.20941867	-9.25630040	-20.91394082
N18	-60.38636504	-9.61275446	-18.66522248
H19	-60.83991247	-10.20834074	-17.98069463
C20	-60.17200002	-8.21800001	-18.30200004
H21	-59.74201266	-7.71560301	-19.16884403
C22	-59.06100001	-2.38000001	-17.01000001
H23	-59.50844345	-1.45351948	-17.39201837
C24	-59.51053780	-3.59531813	-17.83676029
H25	-59.07616373	-4.50954254	-17.40825276
H26	-60.59845841	-3.70737880	-17.74766306
C27	-59.11313993	-3.49470703	-19.31935068
H28	-59.56660719	-2.58959275	-19.75241285
H29	-58.02512057	-3.34656260	-19.38288399
C30	-59.49333174	-4.71087660	-20.18826893
H31	-59.05509129	-4.57408503	-21.18861072
H32	-59.03691744	-5.62098815	-19.77627203
C33	-61.00852955	-4.91392933	-20.34241057
H34	-61.46728916	-5.19452040	-19.39038620
H35	-61.47308358	-3.96492013	-20.64168015
N36	-61.42732564	-5.93372832	-21.32750302
H37	-61.21389398	-6.88504363	-21.01367198
H38	-63.02038781	-5.73320194	-21.46964324
H39	-60.93612127	-5.80299114	-22.21150123
C40	-71.94099872	-10.93600003	-25.10799876
H41	-72.66360676	-11.22785940	-24.33781514
C42	-70.60137297	-11.65452181	-24.90988883
H43	-70.70792412	-12.74217689	-24.97252522
H44	-69.89425902	-11.37062376	-25.70219780
C45	-69.94206341	-11.30809614	-23.58517646
O46	-70.12752784	-10.23589370	-23.01230913
O47	-69.16589121	-12.26751861	-23.12577544
C48	-73.16099987	-7.76700007	-16.52300001
H49	-73.69346065	-6.87144852	-16.18063387
C50	-71.67935317	-7.72979505	-16.12020214
H51	-71.20271243	-6.83827622	-16.54908438
H52	-71.59458392	-7.62564836	-15.02917243
C53	-70.92042930	-8.97946772	-16.58500318
H54	-71.35373431	-9.86818429	-16.09960511
H55	-71.08925726	-9.11035614	-17.66293251
C56	-69.40137979	-8.94043391	-16.33942698
H57	-68.97359165	-8.02459915	-16.76929490
H58	-69.19050507	-8.91431615	-15.25980917

C59	-68.75475832	-10.16701884	-16.98351883
H60	-69.08808186	-11.09389992	-16.50204764
H61	-69.01701943	-10.22342600	-18.03903814
N62	-67.26343031	-10.17949056	-16.96160378
H63	-66.89017058	-10.28248106	-16.01667568
H64	-66.90802375	-10.98708251	-17.59632361
H65	-66.80461357	-9.33104042	-17.39328743
C66	-71.18424596	-7.09542103	-20.63304198
H67	-72.09563052	-7.66828683	-20.44037071
H68	-71.17725840	-6.77099908	-21.67907359
C69	-69.92803642	-7.91311216	-20.37829419
O70	-68.81388956	-7.35607693	-20.23263327
N71	-70.07874514	-9.23965841	-20.31118186
H72	-69.20058618	-9.79255002	-20.28380527
H73	-70.90647651	-9.66058463	-20.70678518
C74	-67.38500002	-5.18800002	-22.72700001
H75	-67.87475382	-5.35355196	-21.76671612
C76	-68.22160202	-5.75316591	-23.89178001
H77	-69.18865994	-5.24745642	-23.97335312
H78	-67.68597722	-5.60399464	-24.83871656
C79	-68.45159795	-7.24252171	-23.74370751
O80	-67.56244618	-8.07505815	-23.66893094
O81	-69.75233778	-7.58064722	-23.70218818
C82	-67.78510216	-13.71365795	-20.69377914
H83	-66.91131266	-13.95647757	-21.31057901
H84	-68.66802503	-14.21483567	-21.10319672
O85	-68.04535232	-12.30518237	-20.71059889
H86	-68.71865692	-12.03675007	-22.24686216
O87	-67.55392181	-9.98576086	-19.90607819
P88	-66.77460306	-11.33130021	-20.09877129
O89	-65.70972248	-11.26764358	-21.16910108
O90	-66.42640773	-11.96698303	-18.73570005
O91	-63.58297005	-9.46032191	-18.74275228
P92	-64.19543412	-8.19681156	-18.20203329
O93	-65.79624750	-8.21164533	-18.27253415
O94	-63.67866791	-7.54576149	-16.94683757
O95	-63.83035117	-7.00382014	-19.45099246
P96	-64.68183001	-5.83033376	-20.05052289
O97	-64.03722436	-5.48835008	-21.44558579
O98	-66.16921333	-6.12684697	-20.12210675
O99	-64.38339686	-4.49852571	-19.19139699
C100	-64.58816157	-4.49774556	-17.75854564
H101	-64.23511307	-3.52357210	-17.41055146
H102	-64.02213703	-5.30998513	-17.29052128

O103	-67.54363058	-6.18342398	-17.43688808
H104	-66.79064356	-6.81350864	-17.49908399
Mg105	-66.78112190	-8.10985699	-20.03577289
H106	-65.64977846	-4.61704298	-17.52289404
H107	-67.56819756	-5.83874185	-18.34584907
H108	-59.35561236	-2.48437766	-15.95932992
H109	-57.97034417	-2.25904770	-17.04199331
H110	-73.66882760	-8.64075692	-16.09447137
H111	-73.27108004	-7.82308003	-17.61339105
H112	-71.16911738	-6.20491615	-19.99888755
H113	-72.36757337	-11.18483302	-26.08562514
H114	-71.81335835	-9.85123869	-25.05271500
H115	-66.40058279	-5.65412354	-22.68369834
H116	-67.24446652	-4.10978331	-22.86615251
H117	-63.82624841	-14.22465366	-18.51635956
H118	-64.30834315	-10.72157245	-14.89092806
H119	-61.18807403	-11.53152299	-21.25304055
H120	-61.11471322	-7.73932027	-18.00968207
H121	-59.46754194	-8.15387431	-17.46406905
H122	-67.60702765	-14.04710978	-19.66747911
O123	-65.67583466	-8.64914829	-21.67823113
H124	-65.55684455	-9.64771819	-21.59328942
H125	-66.14627250	-8.48515313	-22.51864098
H126	-69.82058123	-8.55615149	-23.53486277

associative I-Re

C1	-63.97900000	-10.52500000	-15.92200000
H2	-64.95191195	-10.38585181	-16.41924459
H3	-63.38153037	-9.63359809	-16.07164120
C4	-63.21091807	-11.72656351	-16.42667283
O5	-62.07503693	-11.97724685	-16.01607323
N6	-63.83607496	-12.51268666	-17.35058520
H7	-64.73430412	-12.25091824	-17.76639760
C8	-63.11800000	-13.63200000	-17.92900000
H9	-62.70609001	-14.27647903	-17.16310077
C10	-61.94316761	-13.25705206	-18.87033420
O11	-61.14411045	-14.12004121	-19.22673620
N12	-61.87765766	-11.95634631	-19.26834905
H13	-62.58942752	-11.29518751	-18.96809592
C14	-60.88000000	-11.46800000	-20.20300000
H15	-60.00547713	-12.12468408	-20.13173075
C16	-60.46930722	-9.99470848	-19.94014030
O17	-60.32827713	-9.20289576	-20.87740096
N18	-60.22393391	-9.64058407	-18.64695917
H19	-60.64703859	-10.24510549	-17.94987648

C20	-60.17200000	-8.21800000	-18.30200000
H21	-59.34611113	-7.74334179	-18.83936916
C22	-59.06100000	-2.38000000	-17.01000000
H23	-59.47317572	-1.46912631	-17.46219949
C24	-59.39859033	-3.61868003	-17.85355903
H25	-59.01286337	-4.51876648	-17.35415713
H26	-60.48930709	-3.73438117	-17.88750445
C27	-58.83379495	-3.55566822	-19.28437371
H28	-59.20567258	-2.64395532	-19.77683536
H29	-57.74157137	-3.44437059	-19.22905939
C30	-59.16483752	-4.77497403	-20.17200052
H31	-58.63530968	-4.67158484	-21.13038991
H32	-58.78188330	-5.69211095	-19.70476435
C33	-60.66926292	-4.91770593	-20.43506016
H34	-61.20820333	-5.08211352	-19.49984874
H35	-61.05605111	-3.97726070	-20.85006420
N36	-61.09977729	-5.99689947	-21.35091464
H37	-60.86463203	-6.93638609	-21.00629148
H38	-62.62952734	-5.80547759	-21.41529562
H39	-60.65610013	-5.90532506	-22.26434804
C40	-71.94100000	-10.93600000	-25.10800000
H41	-71.18995677	-11.72520143	-25.19450496
C42	-71.84521680	-10.26361257	-23.72736707
H43	-70.86528743	-9.78324294	-23.60061180
H44	-72.58444850	-9.45842027	-23.62271455
C45	-72.02824360	-11.22046949	-22.55790349
O46	-72.04817782	-10.58384011	-21.37476533
O47	-72.13521209	-12.42965755	-22.66938094
C48	-73.16100000	-7.76700000	-16.52300000
H49	-72.89931867	-7.05845099	-15.72833610
C50	-72.35148451	-9.06623849	-16.39761755
H51	-71.27873668	-8.83134444	-16.42307916
H52	-72.54188624	-9.51128104	-15.40957343
C53	-72.66336851	-10.11093751	-17.48375912
H54	-73.74978870	-10.28976835	-17.51457933
H55	-72.38685409	-9.71174679	-18.46819741
C56	-71.94176387	-11.44598967	-17.23607922
H57	-70.85391192	-11.29072977	-17.24003315
H58	-72.19397133	-11.79700114	-16.22486580
C59	-72.28444278	-12.58029271	-18.21228418
H60	-71.96812466	-13.53466937	-17.76584052
H61	-73.37229248	-12.63570228	-18.35321144
N62	-71.65649029	-12.40687252	-19.53927867
H63	-71.98958639	-11.28709105	-20.58048463

H64	-70.63059524	-12.48768211	-19.43882197
H65	-71.92457892	-13.18236519	-20.14823315
C66	-71.18400000	-7.09500000	-20.63300000
H67	-71.91773073	-7.82958916	-20.99433276
H68	-70.96681937	-6.37705052	-21.42187657
C69	-69.89510191	-7.75833935	-20.19273061
O70	-68.95379106	-7.03972516	-19.76999181
N71	-69.83305191	-9.08281541	-20.25997036
H72	-68.96933563	-9.55475027	-19.94809062
H73	-70.58427820	-9.61187971	-20.69617950
C74	-67.38500000	-5.18800000	-22.72700000
H75	-67.92443279	-5.84548266	-22.04143234
C76	-67.58460918	-5.60859996	-24.18664686
H77	-68.63701782	-5.59817520	-24.48687894
H78	-67.04588794	-4.92337301	-24.85632918
C79	-67.01268834	-6.99341677	-24.38521214
O80	-65.94631598	-7.33168528	-23.88048815
O81	-67.76794078	-7.80087623	-25.11945702
C82	-64.66400000	-8.97600000	-26.19600000
H83	-63.92903123	-8.93657669	-27.00905492
C84	-64.61582628	-10.35511804	-25.51882010
H85	-64.81172289	-11.14020679	-26.26343539
H86	-63.62626089	-10.55205341	-25.09750349
C87	-65.65390105	-10.52987803	-24.41779606
O88	-65.18048716	-11.11108652	-23.34266294
O89	-66.83340891	-10.18931183	-24.55147137
C90	-68.57771002	-13.88472584	-20.58152677
H91	-69.54272526	-14.11248071	-21.05054249
H92	-68.04382428	-14.83393800	-20.41847685
O93	-68.83408671	-13.21036251	-19.35485306
H94	-67.97233159	-12.81499196	-19.07052660
O95	-67.37479327	-9.70995798	-19.18113525
P96	-66.46436788	-10.79203903	-19.82675781
O97	-66.79167255	-10.92500669	-21.32751342
O98	-66.31366928	-12.06078050	-19.00809224
O99	-64.90217459	-10.08349765	-19.88587396
P100	-64.23714025	-8.92572176	-19.01675980
O101	-65.28917400	-8.20049127	-18.18191239
O102	-62.94964933	-9.36894091	-18.41599784
O103	-63.87312277	-7.76890010	-20.20508015
P104	-64.31631510	-6.23357385	-20.05473175
O105	-63.66710954	-5.55288456	-21.30154892
O106	-65.80016179	-6.02955664	-19.87808096
O107	-63.47222573	-5.61773296	-18.81307689

C108	-64.02977553	-5.41075939	-17.51045762
H109	-63.28210316	-4.84730442	-16.94698334
H110	-64.22827693	-6.36732215	-17.02410881
O111	-67.42696128	-6.98506159	-17.36980896
H112	-66.59757582	-7.44086724	-17.11067918
Mg113	-67.03756280	-7.71796892	-19.41158733
H114	-64.95026683	-4.82161403	-17.58034429
H115	-67.19369553	-6.04491027	-17.43130769
H116	-59.47217683	-2.46605688	-15.99749750
H117	-57.97586773	-2.24314356	-16.91954486
H118	-74.23944955	-7.96178808	-16.45754437
H119	-72.97194057	-7.27270509	-17.48419579
H120	-71.60687846	-6.55095492	-19.78769433
H121	-71.77712401	-10.20269179	-25.89512088
H122	-72.92424716	-11.39570978	-25.24468807
H123	-66.32834210	-5.21820216	-22.45802781
H124	-67.74907125	-4.16494254	-22.57185900
H125	-64.44457571	-8.18159246	-25.48172641
H126	-65.65140675	-8.78349413	-26.62568713
H127	-63.84008109	-14.21530908	-18.53008718
H128	-64.16235526	-10.65438208	-14.84568456
H129	-61.23712951	-11.49322262	-21.23908639
H130	-61.11539869	-7.72035028	-18.54737799
H131	-59.99010566	-8.13115031	-17.22724516
H132	-67.99375662	-13.26207032	-21.26824913
O133	-66.88257463	-8.21748710	-21.45031317
H134	-66.92950547	-9.19872907	-21.61520247
H135	-66.30629858	-7.84099869	-22.14221133
H136	-65.85643059	-11.12012297	-22.56670640
H137	-67.37887984	-8.72763299	-25.05652080

associative I-TS

C1	-63.97900000	-10.52500000	-15.92200000
H2	-64.96374800	-10.42253400	-16.38160700
H3	-63.39970300	-9.62710200	-16.13449800
C4	-63.19407700	-11.72705100	-16.40732700
O5	-62.06071200	-11.97201500	-15.99008000
N6	-63.81950400	-12.52174400	-17.32283900
H7	-64.69511400	-12.23426400	-17.75360000
C8	-63.11800000	-13.63200000	-17.92900000
H9	-62.70536600	-14.29303900	-17.16211200
C10	-61.92399800	-13.26084000	-18.85240800
O11	-61.12297900	-14.12777100	-19.19426800
N12	-61.86254000	-11.96348100	-19.25670700
H13	-62.59230700	-11.30524200	-18.98389200

C14	-60.88000000	-11.46800000	-20.20300000
H15	-60.00440300	-12.12536400	-20.15747400
C16	-60.45908500	-10.00120600	-19.93991100
O17	-60.24448700	-9.23088700	-20.88102700
N18	-60.28210000	-9.63449300	-18.63935200
H19	-60.75044600	-10.21845400	-17.95512500
C20	-60.17200000	-8.21800000	-18.30200000
H21	-59.44663600	-7.75282500	-18.97249500
C22	-59.06100000	-2.38000000	-17.01000000
H23	-59.50873300	-1.45866600	-17.40319400
C24	-59.45674600	-3.59622700	-17.86185500
H25	-59.02820500	-4.50688000	-17.41976800
H26	-60.54641100	-3.72029500	-17.81634800
C27	-59.00249800	-3.48804300	-19.32804100
H28	-59.43476800	-2.57828500	-19.77217000
H29	-57.91272300	-3.34531400	-19.34892700
C30	-59.35619700	-4.69891300	-20.21727900
H31	-58.88591300	-4.56450700	-21.20224800
H32	-58.92110200	-5.61267800	-19.79002700
C33	-60.86692800	-4.88781600	-20.40396200
H34	-61.35367800	-5.08498100	-19.44666400
H35	-61.30743200	-3.95814900	-20.78760600
N36	-61.29617400	-5.97091500	-21.31652000
H37	-60.97393800	-6.89760300	-21.01317800
H38	-62.84259200	-5.89004300	-21.28825700
H39	-60.92681000	-5.82863400	-22.25612800
C40	-71.94100000	-10.93600000	-25.10800000
H41	-71.03638200	-11.54767800	-25.17010300
C42	-72.05420500	-10.27795900	-23.73190900
H43	-71.19485700	-9.61910000	-23.54944300
H44	-72.94085300	-9.63224700	-23.66828500
C45	-72.11801200	-11.26740800	-22.57429100
O46	-72.21344200	-10.65343900	-21.38379800
O47	-72.08026600	-12.47794700	-22.70608600
C48	-73.16100000	-7.76700000	-16.52300000
H49	-72.86361700	-6.96350400	-15.83887600
C50	-72.15221300	-8.92356200	-16.49616200
H51	-71.15283200	-8.54476400	-16.74958500
H52	-72.07854000	-9.30947100	-15.46867400
C53	-72.50240000	-10.08425700	-17.44135400
H54	-73.53477600	-10.41470500	-17.24558400
H55	-72.48270100	-9.73123900	-18.48045900
C56	-71.55379000	-11.28111000	-17.27380800
H57	-70.51671200	-10.96864500	-17.45659700

H58	-71.59308500	-11.60987900	-16.22519000
C59	-71.86176500	-12.49991700	-18.15517300
H60	-71.37327900	-13.38154500	-17.71362500
H61	-72.94219700	-12.69952200	-18.15695800
N62	-71.41330300	-12.32038500	-19.55171800
H63	-72.01123200	-11.34659000	-20.58816000
H64	-70.37395000	-12.20980800	-19.55993400
H65	-71.58683600	-13.17845300	-20.07846200
C66	-71.18400000	-7.09500000	-20.63300000
H67	-71.97282500	-7.75003700	-21.01175200
H68	-70.84461600	-6.43241800	-21.43649800
C69	-70.00284700	-7.85694200	-20.09787300
O70	-69.05555100	-7.21241600	-19.58171000
N71	-70.03552700	-9.17897300	-20.19402500
H72	-69.23172900	-9.73073800	-19.86073800
H73	-70.79723300	-9.64183300	-20.68513000
C74	-67.38500000	-5.18800000	-22.72700000
H75	-67.77084200	-5.78375200	-21.89640400
C76	-67.79725100	-5.78195800	-24.07561100
H77	-68.88338200	-5.88250800	-24.17367900
H78	-67.46854000	-5.13525800	-24.90257800
C79	-67.15586100	-7.13754700	-24.28414100
O80	-66.07984800	-7.43813500	-23.77780400
O81	-67.85655300	-7.95684800	-25.05989700
C82	-64.66400000	-8.97600000	-26.19600000
H83	-63.90189600	-8.86967400	-26.97702700
C84	-64.47745500	-10.30509100	-25.45331900
H85	-64.52238500	-11.14126700	-26.16605800
H86	-63.50114200	-10.35437800	-24.96356500
C87	-65.55661800	-10.55995000	-24.41221200
O88	-65.09300500	-11.09367100	-23.30618200
O89	-66.74950900	-10.31964400	-24.61389300
C90	-68.60417700	-13.26262300	-20.85214900
H91	-68.96884700	-12.77737800	-21.77077400
H92	-69.28782700	-14.10314400	-20.62584600
O93	-68.53580700	-12.37197400	-19.76401600
H94	-67.21334400	-12.71793100	-18.98146100
O95	-67.58799900	-9.93366100	-19.15296700
P96	-66.78269800	-11.00613800	-19.91282100
O97	-66.89596100	-11.04958400	-21.43526200
O98	-66.29175700	-12.26569900	-19.01946600
O99	-65.14473200	-10.31033100	-19.87875900
P100	-64.44832900	-9.15723400	-19.06143400
O101	-65.45364400	-8.44751400	-18.14951600

O102	-63.11221800	-9.55681200	-18.52861400
O103	-64.15244600	-7.96180000	-20.23852800
P104	-64.50850000	-6.42049400	-19.97400600
O105	-63.88972600	-5.68755800	-21.21003200
O106	-65.97367800	-6.17563600	-19.71033200
O107	-63.56234600	-5.89323200	-18.76495500
C108	-63.92468600	-5.89662900	-17.38010000
H109	-63.22478000	-5.22055900	-16.88247000
H110	-63.84554300	-6.90113500	-16.96207700
O111	-67.45364500	-7.04378700	-17.25712600
H112	-66.58992400	-7.44643400	-17.02955900
Mg113	-67.18346200	-7.93128800	-19.26683600
H114	-64.94342600	-5.52076300	-17.24800600
H115	-67.25160600	-6.12370700	-17.49532900
H116	-59.39414300	-2.49710500	-15.97249200
H117	-57.97264100	-2.24036700	-16.99840800
H118	-74.16267700	-8.10574700	-16.22817100
H119	-73.24310500	-7.33437600	-17.52832100
H120	-71.58848900	-6.45880900	-19.83880200
H121	-71.89825500	-10.17458800	-25.89445500
H122	-72.79613000	-11.59162300	-25.30256500
H123	-66.29748800	-5.15286500	-22.63302600
H124	-67.77565900	-4.16867300	-22.62221100
H125	-64.58352600	-8.13303600	-25.50744900
H126	-65.64713900	-8.93218600	-26.67464300
H127	-63.83354100	-14.20110800	-18.52967900
H128	-64.10030400	-10.61140200	-14.83466400
H129	-61.25842200	-11.48381300	-21.23180500
H130	-61.14110500	-7.71257100	-18.39850000
H131	-59.81660800	-8.12560200	-17.27141600
H132	-67.62413300	-13.70640700	-21.09762500
O133	-67.04313100	-8.30633500	-21.35307300
H134	-67.05192500	-9.26778900	-21.59700100
H135	-66.44898400	-7.87619000	-21.99688100
H136	-65.81470500	-11.16055200	-22.58632100
H137	-67.41345900	-8.85848100	-25.03438700

associative I-Pr

C1	-63.97900000	-10.52500000	-15.92200000
H2	-65.04860079	-10.61529731	-16.07872307
H3	-63.60451228	-9.69002240	-16.52765717
C4	-63.21438421	-11.77107527	-16.33807491
O5	-62.10826357	-12.05588657	-15.88426705
N6	-63.82416487	-12.54007080	-17.29484991
H7	-64.67563846	-12.22035331	-17.74356312

C8	-63.11800000	-13.63200000	-17.92900000
H9	-62.66102081	-14.27421243	-17.15649707
C10	-61.94792856	-13.22993198	-18.86159683
O11	-61.14286900	-14.08607888	-19.21782500
N12	-61.89648633	-11.92870543	-19.27050777
H13	-62.64257853	-11.28372557	-19.02632905
C14	-60.88000000	-11.46800000	-20.20300000
H15	-60.09048311	-12.22511583	-20.21544138
C16	-60.26041582	-10.08701977	-19.89843792
O17	-59.67405147	-9.48744268	-20.79754357
N18	-60.36675516	-9.62810550	-18.62095738
H19	-61.02053349	-10.12012907	-18.02599498
C20	-60.17200000	-8.21800000	-18.30200000
H21	-59.68222256	-7.75184901	-19.16027521
C22	-59.06100000	-2.38000000	-17.01000000
H23	-59.54522390	-1.39828667	-17.08539411
C24	-59.96053237	-3.48905014	-17.58930157
H25	-59.46889597	-4.46351732	-17.46287974
H26	-60.88818145	-3.53778723	-17.00312252
C27	-60.29180139	-3.27811594	-19.07768382
H28	-60.76625485	-2.29224330	-19.20091322
H29	-59.35245557	-3.23443241	-19.64595029
C30	-61.19923389	-4.34389175	-19.73248896
H31	-61.30008412	-4.08891141	-20.79764732
H32	-60.72331784	-5.33171788	-19.69787489
C33	-62.59367887	-4.41182948	-19.09857731
H34	-62.60968141	-5.02237721	-18.19360745
H35	-62.93784027	-3.40923166	-18.82071517
N36	-63.65778619	-4.97611942	-19.98507999
H37	-63.68937998	-6.03284072	-20.11618976
H38	-64.59288859	-4.81181746	-19.47221316
H39	-63.67081973	-4.53391570	-20.90596550
C40	-71.94100000	-10.93600000	-25.10800000
H41	-71.01998787	-10.41672312	-24.83077641
C42	-73.01180045	-10.82590922	-24.01898268
H43	-73.23866951	-9.77914780	-23.79639116
H44	-73.93714038	-11.30576623	-24.36190588
C45	-72.58564963	-11.53930858	-22.74404661
O46	-72.44764775	-10.71150373	-21.69882593
O47	-72.38235156	-12.74100405	-22.68617291
C48	-73.16100000	-7.76700000	-16.52300000
H49	-72.85021119	-7.00041181	-15.80393891
C50	-72.14963718	-8.92266190	-16.56854044
H51	-71.15603750	-8.53184361	-16.82117472

H52	-72.06083400	-9.35660111	-15.56155011
C53	-72.50904483	-10.04382627	-17.56075738
H54	-73.54279550	-10.37890963	-17.37939553
H55	-72.48750543	-9.65333954	-18.58718709
C56	-71.56041767	-11.24823413	-17.43604234
H57	-70.52291736	-10.92466532	-17.59605101
H58	-71.60484625	-11.61668161	-16.40120607
C59	-71.86046973	-12.43938437	-18.35864696
H60	-71.38905903	-13.33962989	-17.93608023
H61	-72.94088879	-12.63188842	-18.39317036
N62	-71.39753865	-12.22179711	-19.74866229
H63	-72.10862567	-11.24467819	-20.87323605
H64	-70.38581673	-12.06979746	-19.73915518
H65	-71.54763201	-13.06489181	-20.30615136
C66	-71.18400000	-7.09500000	-20.63300000
H67	-71.89316349	-7.51279531	-21.34725396
H68	-70.62258889	-6.28165322	-21.12185555
C69	-70.20629265	-8.09419872	-20.10411546
O70	-69.48321353	-7.80086766	-19.12617398
N71	-70.13471616	-9.26143265	-20.73669677
H72	-69.45408574	-9.94313911	-20.38899898
H73	-70.80090389	-9.51847798	-21.45380790
C74	-67.38500000	-5.18800000	-22.72700000
H75	-67.09213508	-5.60072615	-21.75827648
C76	-68.28944512	-6.16972357	-23.46929567
H77	-69.18499507	-6.41256630	-22.88856505
H78	-68.64895638	-5.74066973	-24.41643827
C79	-67.57824666	-7.46791270	-23.80413356
O80	-66.36801098	-7.59652232	-23.80588307
O81	-68.43579554	-8.44844059	-24.12415732
C82	-64.66400000	-8.97600000	-26.19600000
H83	-63.87399758	-8.79983998	-26.93541016
C84	-64.62793475	-10.43671914	-25.71668460
H85	-64.80899952	-11.11171720	-26.56294762
H86	-63.65626706	-10.68481168	-25.28132965
C87	-65.71642904	-10.67077552	-24.68708658
O88	-65.24766978	-11.01485983	-23.50440616
O89	-66.90893493	-10.53135694	-24.95732518
C90	-67.20383223	-13.82795574	-21.09625019
H91	-67.18938780	-13.42787676	-22.11484981
H92	-67.83417508	-14.71955586	-21.05865862
O93	-67.77438724	-12.88088979	-20.18464225
H94	-65.53647163	-10.62395015	-19.03169961
O95	-68.12795241	-10.55289387	-19.28770238

P96	-67.16770230	-11.37471450	-20.15707743
O97	-66.99482865	-10.83453008	-21.56775681
O98	-65.76524333	-11.55618219	-19.38225421
O99	-64.56420516	-7.49334117	-20.14165387
P100	-64.42114015	-8.23294584	-18.79606177
O101	-65.67311634	-9.13635397	-18.50504231
O102	-63.12687369	-8.93429634	-18.49015544
O103	-64.56542923	-6.97525909	-17.64720755
P104	-65.85408499	-5.98051415	-17.51984725
O105	-65.70698830	-4.75509170	-18.39182011
O106	-67.13228422	-6.81144077	-17.64029002
O107	-65.68128640	-5.46175428	-15.98723158
C108	-65.70734017	-6.40212704	-14.91882533
H109	-65.47640693	-5.84989830	-14.00342379
H110	-64.95812503	-7.18808130	-15.06639968
O111	-67.98595521	-9.10839203	-16.67693500
H112	-67.18487605	-9.65377045	-16.59178072
Mg113	-67.60771684	-8.53867639	-18.76547843
H114	-66.70113658	-6.85636830	-14.81449058
H115	-67.67731672	-8.19162058	-16.49751793
H116	-58.83995376	-2.56446997	-15.95265165
H117	-58.10626178	-2.32304056	-17.54771959
H118	-74.15928937	-8.11859863	-16.23031337
H119	-73.25609072	-7.28169676	-17.50288290
H120	-71.73017524	-6.63657348	-19.79768255
H121	-72.30206652	-10.49015200	-26.05121114
H122	-71.69706902	-11.98598094	-25.30588223
H123	-66.47545247	-4.99542503	-23.30443425
H124	-67.90122285	-4.23571106	-22.55786000
H125	-64.53105591	-8.28591635	-25.35999733
H126	-65.63075966	-8.75447227	-26.65776552
H127	-63.81687495	-14.23038858	-18.49965883
H128	-63.76562921	-10.32962651	-14.86496430
H129	-61.27498813	-11.39070366	-21.22294765
H130	-61.14505026	-7.75496715	-18.11651140
H131	-59.52968715	-8.10583742	-17.41936551
H132	-66.18434058	-14.09039901	-20.79139792
O133	-67.10412995	-8.08957412	-20.78253069
H134	-67.07501255	-8.93834471	-21.28065296
H135	-66.14928160	-7.79551564	-20.73565683
H136	-65.96896171	-11.01222495	-22.79774771
H137	-67.90538527	-9.25949710	-24.35213086

associative II Re

C1	-63.97899994	-10.52500002	-15.92200042

H2	-64.94928609	-10.41141650	-16.40546331
H3	-63.37958249	-9.64400678	-16.16525750
C4	-63.24360791	-11.78338319	-16.34334700
O5	-62.18626009	-12.13896892	-15.82731494
N6	-63.84219952	-12.51377552	-17.34494158
H7	-64.55182852	-12.03384346	-17.89346551
C8	-63.11800066	-13.63199919	-17.92900064
H9	-62.71260371	-14.25791549	-17.13281723
C10	-61.91380798	-13.23445497	-18.81887370
O11	-61.03363544	-14.04907707	-19.06921251
N12	-61.91459998	-11.94849713	-19.29809083
H13	-62.75986288	-11.39360011	-19.19782598
C14	-60.87999991	-11.46800016	-20.20299966
H15	-60.06149575	-12.19289267	-20.15915856
C16	-60.32869744	-10.06812762	-19.90755864
O17	-59.69931910	-9.46698428	-20.77477691
N18	-60.56777623	-9.56696017	-18.66658792
H19	-61.06564643	-10.14297145	-17.99994281
C20	-60.17200018	-8.21800002	-18.30200007
H21	-59.75867095	-7.75755814	-19.20047688
C22	-59.06100017	-2.38000006	-17.01000014
H23	-59.35375175	-1.39149914	-17.38568730
C24	-59.92326793	-3.49292090	-17.63858408
H25	-59.62978891	-4.46293366	-17.21387514
H26	-60.96938638	-3.33953703	-17.34405336
C27	-59.81235824	-3.55998754	-19.17435480
H28	-60.12855722	-2.59668571	-19.60247271
H29	-58.75482641	-3.67903121	-19.44760199
C30	-60.60670167	-4.69789099	-19.86051607
H31	-60.36006464	-4.69447971	-20.93183082
H32	-60.28057906	-5.66756621	-19.46960206
C33	-62.11554553	-4.53708985	-19.68358966
H34	-62.43052388	-4.68688783	-18.65104220
H35	-62.43465585	-3.53346663	-19.97949809
N36	-62.97261377	-5.47233294	-20.49931623
H37	-63.08969675	-6.43847580	-20.13550237
H38	-63.94643648	-5.03676246	-20.48020361
H39	-62.66532640	-5.55517128	-21.47264405
C40	-71.94099989	-10.93599991	-25.10799968
H41	-71.03620584	-11.50388175	-25.34494644
C42	-71.76078320	-10.13732612	-23.82009163
H43	-70.94966323	-9.40415391	-23.92357087
H44	-72.65855300	-9.54925437	-23.58660580
C45	-71.44611256	-10.99682287	-22.60267056

O46	-71.29705679	-10.25546901	-21.49949097
O47	-71.33695254	-12.21113131	-22.62692375
C48	-73.16099976	-7.76700040	-16.52300024
H49	-73.11818772	-7.03146888	-15.71087664
C50	-72.01974883	-8.78900591	-16.40566963
H51	-71.05611543	-8.26263658	-16.41006398
H52	-72.08887425	-9.28775863	-15.42742556
C53	-72.01789440	-9.86395346	-17.50791942
H54	-73.00041874	-10.36083118	-17.53280161
H55	-71.88865727	-9.39130351	-18.49077759
C56	-70.92584862	-10.92364200	-17.28469317
H57	-69.93424889	-10.45018073	-17.30783481
H58	-71.04021802	-11.33072782	-16.26978189
C59	-70.93379486	-12.11497517	-18.25370666
H60	-70.35402681	-12.93437398	-17.80277978
H61	-71.95985911	-12.48442497	-18.38140858
N62	-70.39635907	-11.78912829	-19.59235459
H63	-70.42060587	-12.62654842	-20.17772558
H64	-71.02839841	-10.85667319	-20.66207002
H65	-69.40118721	-11.52393865	-19.52280367
C66	-71.18399936	-7.09500024	-20.63299999
H67	-71.62868296	-7.95475984	-21.13580937
H68	-71.09373975	-6.26257799	-21.34088404
C69	-69.79701782	-7.40589782	-20.09128888
O70	-69.29630277	-6.66646205	-19.20695791
N71	-69.14291095	-8.44004398	-20.61110705
H72	-68.16187209	-8.61146325	-20.37554756
H73	-69.61270006	-9.07251231	-21.24853873
C74	-67.38499994	-5.18800039	-22.72699974
H75	-67.32358487	-5.19815260	-21.63435689
C76	-67.55941519	-6.59728415	-23.29732405
H77	-68.48382146	-7.07014078	-22.95417954
H78	-67.59102343	-6.55732727	-24.39509025
C79	-66.37668353	-7.45216390	-22.89355758
O80	-65.20903680	-6.97045702	-23.07364769
O81	-66.53594797	-8.59914341	-22.37276920
C82	-64.66399994	-8.97600011	-26.19599955
H83	-64.67951969	-8.99380550	-27.29238059
C84	-63.34808312	-9.58415914	-25.65388280
H85	-63.27732561	-10.63786413	-25.94612864
H86	-62.48075046	-9.04636442	-26.04836931
C87	-63.37114711	-9.47300270	-24.13853306
O88	-62.76128010	-8.52816696	-23.55204546
O89	-64.17027036	-10.23320133	-23.49011138

C90	-67.10442585	-11.80186340	-21.08445938
H91	-67.97848700	-12.29204148	-21.52980345
H92	-66.31186517	-12.55687405	-20.97317435
O93	-67.50638312	-11.26515743	-19.82839498
H94	-66.71781309	-10.81260852	-19.43847018
O95	-66.47237134	-7.96497176	-19.23172877
P96	-65.06898765	-8.62365143	-19.15577373
O97	-64.12911590	-8.16793205	-20.31688374
O98	-65.10657062	-10.13576063	-18.97150219
O99	-64.29938934	-7.96191360	-17.82672586
P100	-64.74455092	-6.71060156	-16.88274079
O101	-66.24083960	-6.85322764	-16.57423086
O102	-63.75316621	-6.49772346	-15.80087971
O103	-64.61957530	-5.46268255	-17.99589402
P104	-65.63752660	-4.33153801	-18.61563898
O105	-65.15045651	-4.02350217	-20.00574358
O106	-67.07838656	-4.78823548	-18.40743805
O107	-65.34242888	-3.02960179	-17.69405632
C108	-65.56872151	-3.10407130	-16.28377758
H109	-65.35481982	-2.11053329	-15.88116068
H110	-64.90181724	-3.83922891	-15.81920942
O111	-68.78880546	-6.67046023	-16.39260326
H112	-67.96410615	-6.63636610	-15.85747927
Mg113	-67.54336487	-6.69756497	-18.16140793
H114	-66.61129975	-3.36507069	-16.06896823
H115	-69.18130764	-5.78333961	-16.35762713
H116	-59.17042583	-2.37052972	-15.91981778
H117	-57.99769733	-2.52307514	-17.24065354
H118	-74.14258776	-8.25665072	-16.47903579
H119	-73.10824381	-7.21766472	-17.47160250
H120	-71.82310415	-6.76396089	-19.81010004
H121	-72.16010647	-10.26782360	-25.94839449
H122	-72.76182394	-11.65421651	-25.01342558
H123	-66.46116569	-4.74069962	-23.10027105
H124	-68.22503203	-4.54140808	-23.01260972
H125	-64.77001332	-7.93827889	-25.86230090
H126	-65.52136109	-9.54463321	-25.82384877
H127	-63.80812540	-14.23366851	-18.53004218
H128	-64.09884455	-10.54406386	-14.83341241
H129	-61.23353327	-11.43801823	-21.24066673
H130	-61.03708001	-7.65012030	-17.94028414
H131	-59.40344657	-8.22131440	-17.51811220
H132	-66.75597025	-11.02365482	-21.77281943
Mg133	-64.40554798	-8.64871464	-22.19875341

associative II-TS

C1	-63.97900000	-10.52500000	-15.92200000
H2	-64.98378400	-10.46833500	-16.34405500
H3	-63.44191200	-9.61004500	-16.16803200
C4	-63.17603200	-11.70890200	-16.42565600
O5	-62.02271800	-11.91931100	-16.04549200
N6	-63.81686400	-12.52611400	-17.30922100
H7	-64.69970900	-12.23462700	-17.72358700
C8	-63.11800000	-13.63200000	-17.92900000
H9	-62.70026100	-14.29937400	-17.16998900
C10	-61.92575100	-13.25069200	-18.84860200
O11	-61.09601800	-14.10288500	-19.15650900
N12	-61.89476000	-11.96442200	-19.28959200
H13	-62.63581200	-11.31158200	-19.03560300
C14	-60.88000000	-11.46800000	-20.20300000
H15	-59.99590100	-12.10688600	-20.10133400
C16	-60.50572900	-9.99093200	-19.94100600
O17	-60.33936200	-9.20182200	-20.87336200
N18	-60.30571700	-9.63139000	-18.63987600
H19	-60.71819900	-10.23824200	-17.93858200
C20	-60.17200000	-8.21800000	-18.30200000
H21	-59.43519100	-7.76413700	-18.96816500
C22	-59.06100000	-2.38000000	-17.01000000
H23	-59.43411500	-1.41088100	-17.36441300
C24	-59.63088600	-3.53530600	-17.84927600
H25	-59.26350500	-4.49115400	-17.44966300
H26	-60.72234400	-3.55652200	-17.73288800
C27	-59.26602000	-3.44198300	-19.34143700
H28	-59.67018600	-2.50480200	-19.75377100
H29	-58.17324600	-3.36364200	-19.43046200
C30	-59.73952200	-4.62421900	-20.21294200
H31	-59.31448300	-4.51011500	-21.22074500
H32	-59.33478800	-5.56405000	-19.81420500
C33	-61.26525100	-4.73480700	-20.33014900
H34	-61.72108400	-4.92559700	-19.35551400
H35	-61.67884600	-3.78205100	-20.68526900
N36	-61.77183200	-5.78884700	-21.24031700
H37	-61.48989200	-6.72697300	-20.93669000
H38	-63.30432300	-5.66324700	-21.22735400
H39	-61.39362100	-5.67193900	-22.18058900
C40	-71.94100000	-10.93600000	-25.10800000
H41	-71.11815100	-11.65123900	-25.19043200
C42	-71.93406500	-10.26462700	-23.73088600
H43	-70.99952200	-9.70521200	-23.57824800

H44	-72.74261000	-9.52763300	-23.63827100
C45	-72.05677700	-11.23880100	-22.57060600
O46	-72.27433500	-10.61151900	-21.39454000
O47	-71.95942500	-12.44886700	-22.66434400
C48	-73.16100000	-7.76700000	-16.52300000
H49	-72.85343700	-7.00899000	-15.79257100
C50	-72.36177800	-9.06527400	-16.35165000
H51	-71.29011900	-8.84789200	-16.45725300
H52	-72.49772000	-9.44183800	-15.32669100
C53	-72.74207100	-10.16892100	-17.35037700
H54	-73.81985300	-10.38097900	-17.27213900
H55	-72.56743800	-9.80837200	-18.37054000
C56	-71.95211900	-11.46523800	-17.11846100
H57	-70.87460700	-11.24868300	-17.12103600
H58	-72.18530600	-11.84826400	-16.11424300
C59	-72.21549900	-12.59083000	-18.12737800
H60	-71.81803700	-13.52930300	-17.70995900
H61	-73.29665200	-12.73421000	-18.26120500
N62	-71.61264300	-12.30831600	-19.44501600
H63	-71.68309500	-13.13408400	-20.04162100
H64	-72.15767700	-11.28567200	-20.61036000
H65	-70.59516900	-12.15156600	-19.32334600
C66	-71.18399900	-7.09500000	-20.63300000
H67	-72.15696200	-7.59418400	-20.61484900
H68	-70.84303100	-7.06601400	-21.67387700
C69	-70.15280700	-7.85106300	-19.84661300
O70	-69.21475400	-7.22055200	-19.28030200
N71	-70.29040000	-9.16779600	-19.80973600
H72	-69.48877900	-9.72712900	-19.49056100
H73	-70.97281700	-9.60171600	-20.42819100
C74	-67.38500000	-5.18800000	-22.72700000
H75	-67.42421300	-5.15459900	-21.63497000
C76	-68.29003900	-6.29368400	-23.27657900
H77	-69.33320400	-6.14498600	-22.97995100
H78	-68.27960100	-6.27669600	-24.37656500
C79	-67.85790100	-7.69945200	-22.87906400
O80	-66.61527200	-7.95629700	-22.77211300
O81	-68.72299800	-8.61825700	-22.72346500
C82	-64.66400000	-8.97600000	-26.19600000
H83	-64.12762000	-8.77667500	-27.13223900
C84	-65.27595100	-10.38795800	-26.19164400
H85	-65.98053500	-10.51879300	-27.01850000
H86	-64.48195200	-11.13859100	-26.27517600
C87	-65.99277200	-10.55460900	-24.86703400

O88	-65.29771300	-10.62748700	-23.80949600
O89	-67.26518200	-10.46503700	-24.81126300
C90	-68.68168600	-13.31938000	-20.24206900
H91	-69.15816700	-13.02647400	-21.19082300
H92	-69.26891400	-14.15470800	-19.81675100
O93	-68.61264200	-12.24968300	-19.32491700
H94	-67.27423400	-12.42401600	-18.60531500
O95	-67.76241500	-9.73367700	-19.11893100
P96	-66.94389900	-10.86054700	-19.78073300
O97	-67.13037100	-11.16986100	-21.25795600
O98	-66.35351200	-11.96051700	-18.75404800
O99	-65.40394900	-10.03274900	-19.96027600
P100	-64.59627500	-9.05795100	-19.00782700
O101	-65.54100400	-8.43094300	-17.96210500
O102	-63.26548000	-9.57741600	-18.58491100
O103	-64.28289000	-7.75878800	-20.04388000
P104	-64.87450000	-6.29015900	-19.83408600
O105	-64.35411400	-5.49325500	-21.06492600
O106	-66.36456400	-6.26569700	-19.56842800
O107	-64.04730600	-5.61099500	-18.61206100
C108	-64.50170000	-5.57896600	-17.25752200
H109	-63.85176500	-4.87345200	-16.73393800
H110	-64.42843000	-6.56944800	-16.80364800
O111	-67.62203800	-7.56423600	-16.63746000
H112	-66.73866500	-7.96618300	-16.48950100
Mg113	-67.38575600	-7.84261700	-18.73256300
H114	-65.53569500	-5.21999300	-17.21030500
H115	-67.57945000	-6.65695000	-16.29935700
H116	-59.34059400	-2.48025300	-15.95504300
H117	-57.96555000	-2.35332300	-17.06500400
H118	-74.23694500	-7.94060200	-16.39164000
H119	-73.01547100	-7.34251600	-17.52404400
H120	-71.27286700	-6.07258500	-20.25995900
H121	-71.82455700	-10.18519200	-25.89683600
H122	-72.87606400	-11.48049400	-25.28085900
H123	-66.34511600	-5.36838000	-23.00747100
H124	-67.69341900	-4.20904000	-23.11595500
H125	-63.96726400	-8.86968700	-25.36040500
H126	-65.44594400	-8.21717900	-26.08127000
H127	-63.83641500	-14.19835600	-18.52884700
H128	-64.05269400	-10.60360000	-14.82956700
H129	-61.21231100	-11.51114300	-21.24654100
H130	-61.12978100	-7.69031800	-18.40605100
H131	-59.82652600	-8.13182400	-17.26774400

H132	-67.68778400	-13.72134000	-20.49931800
Mg133	-67.01482600	-9.99033900	-22.80305800

associative II-Pr

C1	-63.97900000	-10.52500000	-15.92200000
H2	-64.86917178	-10.34010570	-16.51635189
H3	-63.32039234	-9.65895184	-16.01367081
C4	-63.20464613	-11.75249338	-16.36604402
O5	-62.11309989	-12.05454875	-15.88671315
N6	-63.81795613	-12.52296629	-17.31830549
H7	-64.63476601	-12.16229032	-17.79775981
C8	-63.11800000	-13.63200000	-17.92900000
H9	-62.66344434	-14.25570870	-17.15320921
C10	-61.96144035	-13.25434787	-18.89720695
O11	-61.20934433	-14.13726159	-19.30648223
N12	-61.86535082	-11.94565983	-19.24792780
H13	-62.55341612	-11.25831935	-18.93256925
C14	-60.88000000	-11.46800000	-20.20300000
H15	-60.03356474	-12.16367506	-20.18906469
C16	-60.38040251	-10.02734339	-19.93241831
O17	-60.06603500	-9.29942510	-20.87177437
N18	-60.24965700	-9.64331168	-18.62350384
H19	-60.78504764	-10.19768258	-17.96511408
C20	-60.17200000	-8.21800000	-18.30200000
H21	-59.53731840	-7.73698104	-19.04832222
C22	-59.06100000	-2.38000000	-17.01000000
H23	-59.51767935	-1.42289042	-17.29233395
C24	-59.70767037	-3.54881768	-17.77016146
H25	-59.25708539	-4.49505631	-17.43883197
H26	-60.76954620	-3.60613784	-17.49784658
C27	-59.56391228	-3.43024403	-19.29763469
H28	-60.02590694	-2.48766402	-19.62993478
H29	-58.49626691	-3.34635157	-19.54563530
C30	-60.16384946	-4.59769353	-20.10829128
H31	-59.90866334	-4.45674151	-21.16860876
H32	-59.69934287	-5.54466775	-19.80431665
C33	-61.68864875	-4.70738576	-19.97821665
H34	-61.97640161	-4.95654309	-18.95251726
H35	-62.14871200	-3.73604793	-20.20258553
N36	-62.34890278	-5.70424332	-20.84783602
H37	-62.08917815	-6.66445953	-20.61169173
H38	-63.81047323	-5.54785753	-20.58609865
H39	-62.10921880	-5.56786915	-21.82941887
C40	-71.94100000	-10.93600000	-25.10800000
H41	-70.90602857	-10.63444742	-24.91443497

C42	-72.83881669	-10.64131767	-23.90215963
H43	-72.82432709	-9.57913985	-23.63860771
H44	-73.88282666	-10.89008110	-24.14160103
C45	-72.46078088	-11.46527535	-22.67979430
O46	-72.56687457	-10.77293129	-21.53507464
O47	-72.11390490	-12.63345102	-22.72944489
C48	-73.16100000	-7.76700000	-16.52300000
H49	-72.81220949	-6.94832119	-15.88304083
C50	-72.10687400	-8.88323510	-16.61309123
H51	-71.16115045	-8.46720609	-16.98460237
H52	-71.89562362	-9.25339105	-15.59913570
C53	-72.51366254	-10.07513559	-17.50040147
H54	-73.50143093	-10.44595523	-17.18388903
H55	-72.62708683	-9.74225203	-18.54078197
C56	-71.49723973	-11.22846389	-17.42332607
H57	-70.50510223	-10.87570963	-17.73652313
H58	-71.39349587	-11.52478376	-16.36972358
C59	-71.85567962	-12.49542800	-18.21777755
H60	-71.30839045	-13.34871648	-17.78886295
H61	-72.92488854	-12.71804053	-18.10656396
N62	-71.57075367	-12.38057970	-19.66696783
H63	-71.82518706	-13.24606592	-20.14552696
H64	-72.26591419	-11.37682316	-20.73747367
H65	-70.56115051	-12.28778458	-19.81985512
C66	-71.18399900	-7.09500000	-20.63300000
H67	-72.01188684	-7.59273365	-21.14702735
H68	-70.66885240	-6.43831133	-21.33935422
C69	-70.18818917	-8.06785233	-20.06014410
O70	-69.31653636	-7.66435045	-19.25665822
N71	-70.26570012	-9.33032107	-20.47113765
H72	-69.54426884	-9.98124588	-20.14656965
H73	-71.00859432	-9.64757215	-21.08328265
C74	-67.38500000	-5.18800000	-22.72700000
H75	-66.52591892	-4.80574560	-22.16811264
C76	-67.95496943	-6.41534793	-22.03965874
H77	-68.26557542	-6.18878302	-21.01694761
H78	-68.84310899	-6.78052226	-22.57699578
C79	-66.98627333	-7.58004467	-21.99800976
O80	-66.05853542	-7.68872808	-22.81845234
O81	-67.18646337	-8.50430537	-21.10751683
C82	-64.66400000	-8.97600000	-26.19600000
H83	-64.52518727	-8.93821011	-27.28384342
C84	-65.11010684	-10.38066255	-25.74340032
H85	-66.04138973	-10.67327581	-26.23924377

H86	-64.33369443	-11.11229958	-25.99865978
C87	-65.31959331	-10.38509625	-24.23442854
O88	-64.29816826	-10.23537122	-23.48786901
O89	-66.49009853	-10.43054941	-23.74850846
C90	-68.16981013	-13.04932455	-21.89892110
H91	-67.61419620	-12.37995902	-22.56008946
H92	-69.14176322	-13.29536925	-22.33381177
O93	-68.45519874	-12.39779774	-20.64126833
H94	-67.20949371	-12.40342680	-17.88074924
O95	-68.07620318	-10.46389477	-19.14381092
P96	-67.28631706	-11.55978983	-19.86338197
O97	-66.13862707	-11.27203811	-20.78799438
O98	-66.73556395	-12.58691094	-18.70830185
O99	-64.29587950	-8.77197758	-20.60303679
P100	-64.25388899	-8.84376259	-19.07888122
O101	-65.62296337	-9.19479276	-18.44126265
O102	-63.05803813	-9.49203657	-18.44414633
O103	-64.08265800	-7.15803162	-18.60092170
P104	-65.16563663	-6.03721765	-18.89901646
O105	-64.82149572	-5.33930034	-20.25054719
O106	-66.60010344	-6.50378654	-18.76687194
O107	-64.82218521	-4.89836582	-17.79515510
C108	-64.96135054	-5.21648631	-16.40955724
H109	-64.59967346	-4.35025640	-15.84989238
H110	-64.36334365	-6.09625824	-16.14845544
O111	-67.55856837	-8.29306128	-16.86205927
H112	-66.65523450	-8.68106029	-16.91368943
Mg113	-67.40989409	-8.47745421	-19.06584940
H114	-66.01276160	-5.39291189	-16.15261743
H115	-67.39691454	-7.33489334	-16.94161587
H116	-59.17662769	-2.49849977	-15.92628982
H117	-57.98726055	-2.31142869	-17.22635226
H118	-74.10475980	-8.14136362	-16.10517483
H119	-73.38212273	-7.34522411	-17.51183165
H120	-71.58028414	-6.46607006	-19.83005660
H121	-72.29303934	-10.39473023	-25.99277312
H122	-71.94121522	-12.00714007	-25.32909750
H123	-67.04663365	-5.42957753	-23.73920688
H124	-68.13701636	-4.39122224	-22.79200544
H125	-63.72023868	-8.70885170	-25.71201652
H126	-65.41061849	-8.22513797	-25.91523582
H127	-63.83208442	-14.23968170	-18.48766132
H128	-64.24596625	-10.65009366	-14.86449379
H129	-61.27877326	-11.44787285	-21.22334288

H130	-61.17176309	-7.76659816	-18.30897073
H131	-59.72567918	-8.09255366	-17.30996594
H132	-67.59671928	-13.96715560	-21.72700183
Mg133	-65.52288226	-9.65240765	-21.92444377

associative III- Re

C1	-63.97899999	-10.52499998	-15.92200015
H2	-64.86906942	-10.26824223	-16.50309559
H3	-63.37175690	-9.62858268	-15.77940325
C4	-63.14431651	-11.60788476	-16.55135530
O5	-61.92830426	-11.72758037	-16.37953569
N6	-63.81785618	-12.49777256	-17.35570111
H7	-64.80585924	-12.36425751	-17.53016557
C8	-63.11800015	-13.63199977	-17.92900015
H9	-62.71337432	-14.29278623	-17.15635247
C10	-61.90130895	-13.22819270	-18.80301327
O11	-60.96127559	-13.99632529	-18.96085285
N12	-61.96640201	-11.99594726	-19.39842221
H13	-62.80774501	-11.44166988	-19.29334711
C14	-60.88000098	-11.46799944	-20.20300024
H15	-59.99819194	-12.08018037	-19.98103924
C16	-60.57423354	-9.99860045	-19.94413785
O17	-60.37281223	-9.22806665	-20.91311082
N18	-60.51752241	-9.58920104	-18.67269030
H19	-60.78126961	-10.24238948	-17.93539025
C20	-60.17200067	-8.21800008	-18.30200007
H21	-59.92815912	-7.65509502	-19.19947307
C22	-59.06100093	-2.38000047	-17.01000084
H23	-59.64311788	-1.55993425	-16.57145067
C24	-59.96113263	-3.33669190	-17.80004065
H25	-59.35776952	-4.16998506	-18.18828583
H26	-60.69171808	-3.78528360	-17.11552946
C27	-60.68646775	-2.66495438	-18.97753877
H28	-61.26285592	-1.80352366	-18.60132622
H29	-59.93944460	-2.23964985	-19.66272023
C30	-61.60153195	-3.61933198	-19.77059441
H31	-61.89594804	-3.15815230	-20.72385563
H32	-61.04125032	-4.52352822	-20.03950311
C33	-62.85797022	-4.07453844	-19.02502685
H34	-63.31456249	-4.92507127	-19.53050411
H35	-62.64256439	-4.37861768	-17.99946571
N36	-63.91402556	-3.00726007	-18.96215068
H37	-64.60149023	-3.10924930	-19.76617399
H38	-64.48177728	-3.07309917	-18.04696282
H39	-63.50367268	-2.07213303	-18.98615678

C40	-71.94099987	-10.93599995	-25.10799971
H41	-71.71282557	-12.00585845	-25.13378949
C42	-70.93492059	-10.19888981	-24.21717437
H43	-69.93336223	-10.24613792	-24.66980595
H44	-71.17709676	-9.13273441	-24.14359532
C45	-70.79356920	-10.75936519	-22.79022950
O46	-71.03679783	-11.95667239	-22.55085808
O47	-70.38204031	-9.90551491	-21.90749553
C48	-73.16099969	-7.76700027	-16.52300036
H49	-73.20477926	-6.81324766	-15.98547367
C50	-72.02025693	-8.64689681	-15.98230019
H51	-71.06798480	-8.10378484	-16.04347174
H52	-72.20106416	-8.82384476	-14.91359502
C53	-71.86591230	-10.01744200	-16.68041307
H54	-71.37906748	-10.71559523	-15.98522596
H55	-72.86359250	-10.43776917	-16.88298226
C56	-71.03937510	-9.99250469	-17.98108855
H57	-71.44591086	-9.25358159	-18.68171396
H58	-70.01449402	-9.67614745	-17.74904432
C59	-71.02714498	-11.36710227	-18.65963151
H60	-70.73976642	-12.14816816	-17.94649128
H61	-72.02369935	-11.61388990	-19.04058486
N62	-70.08653007	-11.44234974	-19.81692795
H63	-70.29002606	-12.25592477	-20.41312929
H64	-70.22321440	-10.65810964	-20.56936656
H65	-69.07689880	-11.47724261	-19.52107675
C66	-71.18399890	-7.09500023	-20.63299958
H67	-71.35714398	-8.00511875	-21.20981358
H68	-71.39365372	-6.21977062	-21.25999320
C69	-69.75562682	-7.00133637	-20.14328979
O70	-69.43435615	-6.13785252	-19.28801918
N71	-68.88488658	-7.85175723	-20.68542094
H72	-69.20974814	-8.55980801	-21.34842704
H73	-67.90569788	-7.78600823	-20.39542985
C74	-67.38499892	-5.18800070	-22.72699986
H75	-66.45578312	-4.89573889	-23.22538888
C76	-67.83440896	-4.09658369	-21.73829436
H77	-67.96169209	-3.14992794	-22.27822493
H78	-68.78740516	-4.36025771	-21.27208331
C79	-66.77947049	-3.89096310	-20.65259588
O80	-65.66365928	-3.41235682	-21.03357584
O81	-67.02884886	-4.25102348	-19.46696472
C82	-64.66399977	-8.97599948	-26.19599906
H83	-64.87986089	-8.93966603	-27.27073994

C84	-64.37587098	-7.57781029	-25.63866744
H85	-63.50579252	-7.11493737	-26.11486617
H86	-65.23632029	-6.92014068	-25.82385090
C87	-64.15014827	-7.63310841	-24.13587309
O88	-64.90336895	-8.39847643	-23.44619036
O89	-63.22091324	-6.96718274	-23.58968750
C90	-66.68485432	-11.83232668	-20.47399713
H91	-67.42266742	-11.88926530	-21.28670162
H92	-66.12076908	-12.77597454	-20.46544353
O93	-67.34008017	-11.64322164	-19.21614389
H94	-66.99249446	-10.78342206	-18.83633295
O95	-66.42390072	-7.06138814	-19.36952387
P96	-65.54991674	-8.30897053	-18.98581994
O97	-64.77725254	-8.81007966	-20.21489052
O98	-66.39884649	-9.36480804	-18.27210509
O99	-64.35657386	-7.73845569	-18.00871469
P100	-64.40121299	-7.11839162	-16.44388708
O101	-65.64870877	-7.60504038	-15.74949578
O102	-63.01352907	-7.31659442	-15.92675841
O103	-64.61700448	-5.50496835	-16.80941319
P104	-65.71652559	-4.43206991	-16.22330377
O105	-65.20695731	-3.08162555	-16.66424259
O106	-67.11314161	-4.89460067	-16.63654958
O107	-65.60012263	-4.49037966	-14.61381363
C108	-66.50155816	-5.26229641	-13.80836125
H109	-66.31002039	-4.96370672	-12.77334651
H110	-66.30760323	-6.32856151	-13.94148985
O111	-68.03811117	-7.51626086	-17.02355861
H112	-67.68649275	-8.31357542	-17.49080628
Mg113	-67.63772385	-5.83852744	-18.30492473
H114	-67.54004901	-5.03861612	-14.07124589
H115	-67.31821267	-7.40391343	-16.34607423
H116	-58.55340663	-2.90106504	-16.19019677
H117	-58.28991935	-1.93394785	-17.65194986
H118	-74.13254664	-8.26513357	-16.40887962
H119	-73.02798775	-7.53630468	-17.58723145
H120	-71.86073975	-7.04150899	-19.77451521
H121	-71.92014241	-10.54444911	-26.13198549
H122	-72.96252922	-10.82814756	-24.72417296
H123	-67.21786651	-6.14562392	-22.23092875
H124	-68.14888013	-5.34076164	-23.49978638
H125	-65.51886508	-9.41840315	-25.67960544
H126	-63.80305589	-9.64209826	-26.05536768
H127	-63.81963993	-14.20439356	-18.54418186

H128	-64.30621457	-10.86284628	-14.92921498
H129	-61.08907073	-11.56024891	-21.27333989
H130	-60.99829220	-7.75138278	-17.75250964
H131	-59.29402515	-8.23096152	-17.64629976
H132	-65.99805348	-11.00598310	-20.68515815
Mg133	-63.86739031	-7.82456683	-21.71310438
O134	-62.27823046	-7.24149641	-20.44202066
H135	-61.50022844	-7.83038015	-20.55548818
H136	-62.66542815	-7.43604075	-19.56454775
O137	-62.72508854	-9.61577631	-22.17526716
H138	-61.75059032	-9.50278728	-22.13342481
H139	-62.99243061	-9.80952543	-23.08693804
O140	-64.74695530	-6.05765175	-21.13742982
H141	-65.45347109	-6.23561933	-20.45954332
H142	-64.84131039	-5.12612054	-21.41573822

associative III-TS

C1	-63.97900000	-10.52500000	-15.92200000
H2	-64.93049000	-10.38589600	-16.43178000
H3	-63.39436600	-9.61081800	-15.97480200
C4	-63.16140100	-11.67360900	-16.46104200
O5	-61.98438100	-11.87244600	-16.15158000
N6	-63.81733400	-12.51322700	-17.32503900
H7	-64.73924600	-12.24769100	-17.65381200
C8	-63.11800000	-13.63200000	-17.92900000
H9	-62.71175800	-14.29852500	-17.16340600
C10	-61.91234200	-13.23855100	-18.82234300
O11	-61.04524800	-14.06394700	-19.08635200
N12	-61.90498400	-11.95634000	-19.29767100
H13	-62.69549800	-11.35474100	-19.09130300
C14	-60.88000100	-11.46800000	-20.20300000
H15	-60.02742200	-12.15068600	-20.11064500
C16	-60.42843800	-10.02454900	-19.91755300
O17	-60.20927800	-9.25021100	-20.86767100
N18	-60.29593000	-9.64023900	-18.63178000
H19	-60.70450000	-10.24650000	-17.92518700
C20	-60.17200100	-8.21800000	-18.30200000
H21	-59.48127800	-7.75542600	-19.00808100
C22	-59.06100100	-2.38000000	-17.01000100
H23	-59.89625700	-2.54512800	-16.31914300
C24	-59.20779900	-3.24643200	-18.27673500
H25	-58.33908100	-3.07520100	-18.92978600
H26	-59.16806800	-4.30746200	-17.99209600
C27	-60.49453900	-2.96620500	-19.07445400
H28	-61.37957700	-3.21142400	-18.47218200

H29	-60.55217200	-1.88676000	-19.27299000
C30	-60.59701200	-3.69667100	-20.43266200
H31	-61.42527900	-3.24766000	-20.99454400
H32	-59.68893900	-3.50392600	-21.01848600
C33	-60.83290000	-5.22395000	-20.34374200
H34	-60.05069200	-5.80502100	-20.83843600
H35	-60.87980000	-5.55183200	-19.30322800
N36	-62.14442000	-5.61931800	-20.94252800
H37	-62.38877800	-6.60042000	-20.76620200
H38	-62.94583100	-5.04507300	-20.49248200
H39	-62.22691100	-5.50955300	-21.95812400
C40	-71.94100000	-10.93600000	-25.10800000
H41	-72.88136200	-11.47397700	-24.93641400
C42	-70.79649600	-11.55332100	-24.29274800
H43	-70.65701500	-12.60434400	-24.56238300
H44	-69.86282800	-11.02273700	-24.52392800
C45	-71.01464100	-11.47487000	-22.77341800
O46	-71.11229100	-12.56109100	-22.14310000
O47	-71.06929200	-10.30772200	-22.24522200
C48	-73.16100000	-7.76700000	-16.52300000
H49	-73.24768800	-6.78840100	-16.03727300
C50	-72.04623400	-8.60321800	-15.87627800
H51	-71.09264100	-8.06239500	-15.94124400
H52	-72.26546400	-8.70825000	-14.80520100
C53	-71.86846900	-10.01122500	-16.47649700
H54	-71.25842400	-10.61545500	-15.79132600
H55	-72.85058400	-10.50623700	-16.53148500
C56	-71.20439700	-10.02928500	-17.86233300
H57	-71.74747000	-9.36646900	-18.54916000
H58	-70.18208800	-9.63692300	-17.78345300
C59	-71.16983800	-11.43211900	-18.47389600
H60	-70.63775200	-12.13140200	-17.82086200
H61	-72.18913000	-11.81070700	-18.61089600
N62	-70.49311100	-11.44739200	-19.79566800
H63	-70.80459800	-12.20842700	-20.45548600
H64	-70.75198200	-10.64930800	-20.41423000
H65	-69.41458700	-11.49536100	-19.75990100
C66	-71.18399900	-7.09500000	-20.63300000
H67	-71.81791800	-7.86517000	-21.08200800
H68	-71.47872900	-6.11845200	-21.03513200
C69	-69.72387700	-7.31624900	-20.93147200
O70	-68.87216000	-6.72164500	-20.23281100
N71	-69.41548100	-8.09704600	-21.97169000
H72	-70.08325400	-8.79979600	-22.32995000

H73	-68.42986700	-8.24879400	-22.16224200
C74	-67.38499900	-5.18800100	-22.72700000
H75	-67.59656400	-6.00430700	-23.42668200
C76	-65.88648600	-4.98134900	-22.54851000
H77	-65.40984800	-4.59111900	-23.45486400
H78	-65.68221700	-4.27103500	-21.73805600
C79	-65.22702200	-6.30168100	-22.19401500
O80	-64.08274400	-6.56957000	-22.59520700
O81	-65.97373400	-7.11768400	-21.51025000
C82	-64.66400000	-8.97600000	-26.19599900
H83	-64.67450300	-8.46898100	-27.16822100
C84	-63.78177600	-10.23413900	-26.24073700
H85	-64.11544700	-10.92820800	-27.01782200
H86	-62.74868600	-9.94373600	-26.47595200
C87	-63.76597800	-10.97690800	-24.90298100
O88	-63.97816800	-12.21439400	-24.89011800
O89	-63.55213300	-10.25565700	-23.86532500
C90	-67.55838000	-12.73072900	-20.85896300
H91	-68.48099500	-13.17802100	-21.26173800
H92	-67.05714100	-13.49105900	-20.23416500
O93	-67.85571300	-11.57218600	-20.13059500
H94	-67.97280300	-10.02427800	-20.63955200
O95	-67.39198700	-9.29469400	-20.27352800
P96	-66.31305000	-10.23286200	-19.42503400
O97	-65.24109400	-10.95265200	-20.23632800
O98	-66.63585600	-10.53087600	-17.98641900
O99	-65.46503200	-8.59763900	-19.23231600
P100	-64.32261600	-8.10159100	-18.19509400
O101	-64.91695400	-7.81439000	-16.84269800
O102	-63.09979700	-8.96671600	-18.42645900
O103	-63.90150800	-6.62032400	-18.86065900
P104	-64.75203600	-5.21266500	-18.75288000
O105	-64.06817600	-4.29799900	-19.74914800
O106	-66.23236000	-5.53996600	-18.90043100
O107	-64.46212300	-4.58832200	-17.29511800
C108	-65.31619000	-4.82809600	-16.16396700
H109	-65.03960000	-4.08026400	-15.41544000
H110	-65.15409100	-5.83776300	-15.78335600
O111	-67.59640700	-7.85056000	-17.64342100
H112	-67.50686800	-8.83302600	-17.62715700
Mg113	-66.98326800	-7.23408500	-19.67941600
H114	-66.36567600	-4.69905800	-16.44434500
H115	-66.76799900	-7.60234100	-17.16270000
H116	-58.13235700	-2.61467100	-16.47746500

H117	-59.04461900	-1.31266400	-17.26324700
H118	-74.13235600	-8.27112100	-16.43834200
H119	-72.97336400	-7.58694700	-17.58801700
H120	-71.32592400	-7.06284200	-19.55042500
H121	-71.72509000	-10.97064800	-26.18245800
H122	-72.09557000	-9.89040500	-24.82079800
H123	-67.87020000	-5.41310600	-21.78034900
H124	-67.85565500	-4.28154500	-23.13426500
H125	-64.29052200	-8.26807200	-25.44915700
H126	-65.70203500	-9.23040700	-25.94744200
H127	-63.82577900	-14.19749400	-18.54250200
H128	-64.17931800	-10.72671700	-14.85983100
H129	-61.20560800	-11.49056600	-21.24766600
H130	-61.15511900	-7.74074100	-18.34378500
H131	-59.77054200	-8.12709600	-17.28886000
H132	-66.90264200	-12.52481800	-21.72179500
Mg133	-64.30407500	-10.53631000	-21.97060500
O134	-62.95820500	-9.33680600	-21.08725100
H135	-62.02403700	-9.21718200	-21.35538000
H136	-63.01497600	-9.18885000	-20.09684800
O137	-64.06573900	-12.53966900	-22.32566700
H138	-64.78990200	-13.09444200	-21.99856200
H139	-64.02274600	-12.59920900	-23.34613400
O140	-65.81989100	-9.37855500	-22.76236700
H141	-65.87831100	-8.55069300	-22.18228800
H142	-65.30454700	-9.08918000	-23.53804400

associative III-Pr

C1	-63.97900000	-10.52500000	-15.92200000
H2	-65.01089706	-10.53550629	-16.28249177
H3	-63.52096041	-9.60682194	-16.31236985
C4	-63.17527887	-11.71989103	-16.37551591
O5	-62.02948270	-11.94989992	-15.98162764
N6	-63.80903956	-12.53533957	-17.28010965
H7	-64.70273248	-12.22628340	-17.65458733
C8	-63.11800000	-13.63200000	-17.92900000
H9	-62.42858560	-14.08065256	-17.20649220
C10	-62.25687589	-13.21631656	-19.15439272
O11	-61.90294514	-14.05811583	-19.99037803
N12	-61.85174070	-11.92075696	-19.20673806
H13	-62.43555567	-11.18980364	-18.78928778
C14	-60.88000100	-11.46800000	-20.20300000
H15	-59.98762523	-12.10223839	-20.14604540
C16	-60.50711232	-9.98008958	-19.94617212
O17	-60.44021693	-9.16182435	-20.87005751

N18	-60.21001445	-9.63850068	-18.66213306
H19	-60.53533208	-10.27560749	-17.94219434
C20	-60.17200100	-8.21800000	-18.30200000
H21	-59.40627630	-7.71428767	-18.88642499
C22	-59.06100100	-2.38000000	-17.01000100
H23	-59.88432873	-2.59294617	-16.31763689
C24	-59.16251519	-3.25424722	-18.27219920
H25	-58.30427139	-3.04860564	-18.92945597
H26	-59.07472142	-4.30942534	-17.97789002
C27	-60.46977295	-3.03500772	-19.05366018
H28	-61.33003469	-3.24571424	-18.40394976
H29	-60.54632788	-1.97312454	-19.32607136
C30	-60.63648306	-3.87075138	-20.34049548
H31	-61.60278823	-3.61275067	-20.78577548
H32	-59.84822570	-3.61921066	-21.06602952
C33	-60.62899602	-5.37718460	-20.05829584
H34	-59.61775090	-5.77425673	-19.94836994
H35	-61.19203466	-5.59619157	-19.14989279
N36	-61.31807603	-6.17178948	-21.13391843
H37	-61.26419516	-7.19025370	-20.94148881
H38	-62.34522128	-5.83123594	-21.15150311
H39	-60.89821620	-6.01313199	-22.05252363
C40	-71.94100000	-10.93600000	-25.10800000
H41	-72.88259593	-11.46054620	-24.89652478
C42	-70.78660655	-11.54925432	-24.30396632
H43	-70.65757288	-12.60975181	-24.53901752
H44	-69.84686623	-11.04231598	-24.56596963
C45	-70.98388441	-11.39804397	-22.79421462
O46	-70.83117845	-12.46054331	-22.09614637
O47	-71.27404194	-10.26892780	-22.32115027
C48	-73.16100000	-7.76700000	-16.52300000
H49	-73.28360095	-7.12275995	-15.64480783
C50	-73.44980193	-9.23277709	-16.17429997
H51	-72.76259734	-9.56771281	-15.38464601
H52	-74.46013856	-9.30406309	-15.74873039
C53	-73.35797092	-10.19250520	-17.37305827
H54	-73.80122987	-11.15924816	-17.09411378
H55	-73.97885131	-9.79913114	-18.19225588
C56	-71.93160019	-10.44171539	-17.89234901
H57	-71.42447076	-9.49173555	-18.10421331
H58	-71.33820425	-10.94346994	-17.11518480
C59	-71.95655271	-11.28922681	-19.16442328
H60	-72.49840000	-12.22745205	-18.99802062
H61	-72.45544964	-10.75731095	-19.98031680

N62	-70.61981989	-11.64880231	-19.69421555
H63	-70.73590211	-12.08092143	-20.75318839
H64	-70.00043302	-10.84296879	-19.84698069
H65	-70.08185691	-12.29380611	-19.10653043
C66	-71.18399900	-7.09500000	-20.63300000
H67	-71.85506638	-7.81923060	-21.09542237
H68	-71.42944756	-6.09365973	-20.99926295
C69	-69.73807177	-7.38284296	-20.94381215
O70	-68.83862236	-6.90141321	-20.21710820
N71	-69.49572632	-8.09904132	-22.04425961
H72	-70.20590844	-8.73887439	-22.41627087
H73	-68.51822984	-8.19949131	-22.31574834
C74	-67.38499900	-5.18800100	-22.72700000
H75	-68.17418739	-5.81481880	-23.14235306
C76	-65.99160780	-5.69023609	-23.09838947
H77	-65.83015260	-5.65548208	-24.18514139
H78	-65.21226305	-5.06514327	-22.64851670
C79	-65.70420249	-7.11980824	-22.65324100
O80	-64.59994446	-7.63362713	-22.89582272
O81	-66.67667473	-7.74170293	-22.03914556
C82	-64.66400000	-8.97600000	-26.19599900
H83	-64.51346354	-8.44271468	-27.14250448
C84	-63.78636322	-10.24369769	-26.15800622
H85	-64.08031821	-10.91936042	-26.97573890
H86	-62.73698775	-9.99211882	-26.34554063
C87	-63.80199379	-11.09439348	-24.87189231
O88	-62.79852515	-11.79495622	-24.62077785
O89	-64.86326182	-11.07427128	-24.14096170
C90	-68.04535636	-14.00134081	-20.49602140
H91	-68.66702713	-13.56165251	-21.28527234
H92	-68.53602074	-14.90189879	-20.11506018
O93	-67.91449594	-13.10436129	-19.38178902
H94	-67.73909839	-10.67082399	-21.33129705
O95	-68.03863565	-10.76929816	-20.39383178
P96	-67.00674724	-11.74047198	-19.56056968
O97	-65.82809752	-12.08989781	-20.47077785
O98	-66.80527054	-11.17425992	-18.18779986
O99	-65.32402898	-8.64184370	-19.84770605
P100	-64.25221848	-8.37056495	-18.77204431
O101	-64.82443438	-8.04213573	-17.39886263
O102	-63.09289360	-9.35638704	-18.91493180
O103	-63.52995904	-6.88506596	-19.28229366
P104	-64.33526822	-5.52369059	-19.62640235
O105	-63.63252909	-4.92497912	-20.83419753

O106	-65.84358913	-5.76580180	-19.68337386
O107	-64.01049530	-4.52563911	-18.38258277
C108	-64.66374810	-4.76257375	-17.12261676
H109	-64.18090865	-4.10045754	-16.39787298
H110	-64.55890651	-5.80809087	-16.81619042
O111	-67.39921883	-8.32927333	-18.03797070
H112	-67.33175256	-9.30968996	-18.14544150
Mg113	-66.88612161	-7.46392698	-19.91690081
H114	-65.72682480	-4.51036618	-17.19822375
H115	-66.51259109	-8.14854080	-17.59696072
H116	-58.11970598	-2.55848152	-16.47712879
H117	-59.10730651	-1.31328308	-17.26304471
H118	-73.84683306	-7.40570546	-17.29990355
H119	-72.13735876	-7.62615848	-16.88793918
H120	-71.32816608	-7.08978753	-19.54200816
H121	-71.75382676	-10.99082472	-26.17963746
H122	-72.07721876	-9.88614973	-24.82406817
H123	-67.50940247	-5.13500727	-21.64420991
H124	-67.52891052	-4.16564983	-23.12229985
H125	-64.42154576	-8.30192173	-25.36831387
H126	-65.72654472	-9.23202672	-26.11694710
H127	-63.83041736	-14.38907385	-18.26364134
H128	-63.96073397	-10.47572996	-14.83386947
H129	-61.27056968	-11.51578495	-21.22377850
H130	-61.15958410	-7.76599545	-18.47798166
H131	-59.92820038	-8.13721322	-17.24093843
H132	-67.06193119	-14.26154737	-20.89708912
Mg133	-64.85407132	-11.35960390	-22.06271871
O134	-63.54640763	-9.88539925	-21.65625548
H135	-63.92484043	-9.04206258	-22.02249323
H136	-63.39054513	-9.70742570	-20.69089737
O137	-63.58157678	-12.94740718	-22.35248984
H138	-62.93898395	-13.31088606	-21.71095013
H139	-63.11021756	-12.65463019	-23.19476592
O140	-66.64451024	-10.25357254	-22.57622348
H141	-66.52068838	-9.27804461	-22.31086077
H142	-66.38785743	-10.31124831	-23.52416207
